# Liver Protein Expression in NASH Mice on a High-Fat Diet: Response to Multi-Mineral Intervention

**DOI:** 10.3389/fnut.2022.859292

**Published:** 2022-05-11

**Authors:** James Varani, Shannon D. McClintock, Randall N. Knibbs, Isabelle Harber, Dania Zeidan, Mohamed Ali H. Jawad-Makki, Muhammad N. Aslam

**Affiliations:** Department of Pathology, The University of Michigan Medical School, Ann Arbor, MI, United States

**Keywords:** aquamin, fibrosis, liver injury, liver cancer, mouse model, NAFLD, NASH, proteomics

## Abstract

Male MS-NASH mice were maintained on a high-fat diet for 16 weeks with and without red algae-derived minerals. Obeticholic acid (OCA) was used as a comparator in the same strain and diet. C57BL/6 mice maintained on a standard (low-fat) rodent chow diet were used as a control. At the end of the in-life portion of the study, body weight, liver weight, liver enzyme levels and liver histology were assessed. Samples obtained from individual livers were subjected to Tandem Mass Tag labeling / mass spectroscopy for protein profile determination. As compared to mice maintained on the low-fat diet, all high-fat-fed mice had increased whole-body and liver weight, increased liver enzyme (aminotransferases) levels and widespread steatosis / ballooning hepatocyte degeneration. Histological evidence for liver inflammation and collagen deposition was also present, but changes were to a lesser extent. A moderate reduction in ballooning degeneration and collagen deposition was observed with mineral supplementation. Control mice on the high-fat diet alone demonstrated multiple protein changes associated with dysregulated fat and carbohydrate metabolism, lipotoxicity and oxidative stress. Cholesterol metabolism and bile acid formation were especially sensitive to diet. In mice receiving multi-mineral supplementation along with the high-fat diet, there was reduced liver toxicity as evidenced by a decrease in levels of several cytochrome P450 enzymes and other oxidant-generating moieties. Additionally, elevated expression of several keratins was also detected in mineral-supplemented mice. The protein changes observed with mineral supplementation were not seen with OCA. Our previous studies have shown that mice maintained on a high-fat diet for up to 18 months develop end-stage liver injury including hepatocellular carcinoma. Mineral-supplemented mice were substantially protected against tumor formation and other end-state consequences of high-fat feeding. The present study identifies early (16-week) protein changes occurring in the livers of the high-fat diet-fed mice, and how the expression of these proteins is influenced by mineral supplementation. These findings help elucidate early protein changes that contribute to end-stage liver injury and potential mechanisms by which dietary minerals may mitigate such damage.

## Introduction

Non-alcoholic fatty liver disease (NAFLD) is rapidly becoming the most common cause of liver injury in Western society and throughout the world (1–3). It is estimated that up to 25% of adults worldwide have some degree of hepatic fat accumulation (4). When fat accumulation is the extent of damage, it is well-tolerated by most people. However, in a subset of individuals, disease progression leads to liver inflammation and hepatocyte injury. Cycles of injury and repair result in measurable collagen deposition in the liver parenchyma, leading eventually to detectable fibrotic changes and, in some cases, to cirrhosis. When steatosis is followed by the tissue damage, this state is referred to as non-alcoholic steatohepatitis (NASH) (5). Formation of liver tumors, including hepatocellular carcinoma, is another common and most devastating late-stage consequence. Why some individuals with extensive steatosis progress to more serious disease including liver cancer and others do not, remains a critical unanswered question.

The high content of saturated fat and sugar in the typical Western diet is thought to underlie fat accumulation (6–8). Experimental studies in rodents have shown that providing a high-fat or high-fat and -sugar diet leads rapidly to fatty liver infiltration. While some strains are more vulnerable to developing the NAFLD phenotype than others in response to a high-fat diet (9), fat accumulation can be seen as early as after 8 weeks of feeding in some strains (10–12). With animals maintained for longer periods (20–25 weeks), evidence of inflammatory changes, ballooning hepatocyte degeneration and collagen deposition may also be seen. Even after 25 weeks, however, these changes are generally mild. Some studies have reported widespread liver damage (beyond steatosis) at earlier time-points but, typically, liver toxins such as carbon tetrachloride, thioacetamide or alcohol are administered along with the high-fat diet (13, 14). Animals with severe genetic anomalies (15) or animals maintained on a toxic, nutrient-restricted diet such as the methionine- and choline-deficient diet (16) may develop severe liver anomalies more rapidly. When a high-fat or high-fat and -sugar diet alone is utilized in “genetically normal” strains, overt liver damage (beyond steatosis) is not routinely observed before 36 weeks of age (17) and a year or more is required for terminal damage – including hepatocellular carcinoma – to be widely manifested (18, 19).

In our own previous studies (20, 21), C57BL/6 mice were maintained on a high-fat Western-style diet (HFWD) for up to 18 months. The long feeding period allowed for the development of extensive liver damage in a majority of animals, especially males. In addition to widespread steatosis, HFWD-fed mice demonstrated extensive perivascular and parenchymal liver inflammation, ballooning hepatocyte injury and collagen deposition. Large fibrotic nodules and areas of infarct were observed in many of the animals. Liver tumors including hepatic adenomas and hepatocellular carcinomas were identified grossly and histologically in many of the animals. Of most interest, our studies (20, 21) demonstrated that providing an adequate level of calcium (estimated to be 20–25 mg/day consumed) along with multiple other trace elements in a mineral supplement (Aquamin®) dramatically reduced tumor formation (21). Inflammation, hepatocyte injury and collagen deposition were also reduced while steatosis, itself, was largely unaffected.

While an extended feeding period is essential to see the full spectrum of liver disease develop in rodents, long-term studies are not optimal for identifying the early events that lead to steatosis and disease progression in high-fat-fed mice. Likewise, long-term studies are not ideal for understanding how dietary minerals (or other interventions) might mitigate disease progression. As a way to help understand the early events that drive liver disease progression and its prevention, cohorts of mice (MS-NASH strain) were placed on a high-fat diet with fructose added to the drinking water (high-fat diet) with and without mineral supplementation (Aquamin®) for a period of 16 weeks. An additional cohort of high-fat diet mice was treated with a comparator, obeticholic acid (OCA), a drug that acts as a Farnesoid X Receptor (FXR) agonist, that has been shown to reduce liver damage in both humans (22–25) and mice (26). C57BL/6 mice fed a low-fat rodent chow diet served as an additional control. At the end of the in-life portion of the study, whole-body weight and live weight were obtained from each animal along with liver enzyme levels and metabolic parameters. Livers were assessed histologically for steatosis and for other changes (hepatocyte degeneration, inflammation, and collagen deposition) indicative of more serious damage. Finally, liver tissue samples were evaluated using a tandem mass tag (TMT) mass spectrometry-based proteomic approach for protein expression levels in individual animals. Findings from these relatively short-term studies (especially the proteomic analyses) allow us to identify early liver changes that reflect diet differences (i.e., that distinguish low-fat and high-fat feeding) and how the effects of high-fat feeding may be modified in response to intervention. Findings from this study are described here.

## Materials and Methods

### Experimental Diets and Interventions

Diet D12079B (Research Diets Inc., New Brunswick, NJ) with 5% fructose added to the drinking water was used as the high-fat Western diet for this study. This diet was designed to provide 500 gm/kg of carbohydrates, 198 gm/kg of proteins and 210 gm/kg of fat. The percentage of calories from carbohydrates, proteins and fat in this diet was 43, 17, and 40%, respectively. This diet also contained 39 gm/kg of a standard mineral mix (S10001A) containing calcium carbonate and calcium phosphate and other essential trace elements. Diet 5,008 (Purina) was used as the low-fat control diet. The caloric distribution from this low-fat diet was approximately 57, 27, and 16% from carbohydrates, proteins and fat, respectively. Diet 5,008 was supplemented with standard minerals (2.5%) as part of the diet formula. High-fat diet alone was considered as the control for comparison with the low-fat diet or with Aquamin® and OCA as interventions in the high-fat diet.

Aquamin® is a product rich in calcium, magnesium, and trace elements, obtained from the skeletal remains of red marine algae (27) (Marigot Ltd, Cork, Ireland). Aquamin® contains calcium and magnesium in an approximately 14:1 ratio, along with measurable levels of 72 additional trace minerals including minerals with calcimimetic activity [for example, magnesium, strontium, and trace elements from the lanthanide family (28–30)], and essentially all of the minerals concentrated from ocean water in the algal fronds. Mineral composition was established by an independent laboratory (Advanced Laboratories; Salt Lake City, Utah) using Inductively Coupled Plasma Optical Emission Spectrometry *(ICP-OES*). Supplementary Table 1 provides a complete list of elements detected in Aquamin® and their relative amounts. Aquamin® is sold as a dietary supplement (GRAS 000028) and is used in various products for human consumption in Europe, Asia, Australia, and North America. A single batch of Aquamin®-Soluble (citrate-malate salt) was used for this study. Aquamin®-Soluble was added to the drinking water (18 mg/mL) throughout the entire in-life portion of the study. We have used Aquamin® in previously conducted long-term mouse studies (21, 31, 32).

Obeticholic acid (OCA) was prepared as a 3 mg/mL suspension in 0.5% methylcellulose. Animals received 0.25 mL OCA (approximately 30 mg/kg) by oral gavage five times per week beginning on week-8 of high-fat feeding.

### Mouse Model and In-Life Study Protocol

The study was conducted in a mouse model - MS-NASH mouse (formally known as FATZO [(33, 34)]) by Crown Bioscience, Inc. Three cohorts of male MS-NASH mice (9 animals per group) were maintained on the high-fat diet, beginning at 6–7 weeks of age (at weaning) and after 1 week of acclimation. One group was kept as control (high-fat diet alone) while the other groups received Aquamin® or OCA as interventions along with the high-fat Western diet. Since mineral supplementation (Aquamin®) is proposed as a preventive agent, it was included along with high-fat feeding. OCA is envisioned as a therapeutic agent for existing disease, and animals were started on OCA at week-8 (16 weeks of age). A low-fat diet control group consisted of male C57BL/6 (Envigo) mice (nine animals per group) maintained on Diet 5,008. Food and water were provided *ad libitum*. The animal environment was maintained at 70–74°F with a 12-h light-dark cycle. During the in-life portion of the study, health-checks were done daily. At termination (after 16 weeks on diet), animals were euthanized by carbon dioxide asphyxiation. The entire in-life portion of the study and termination (euthanasia and tissue harvesting) was carried out by Crown Bio, Inc. (at their Lafayette, LA facility) under the approved Standard Operating Procedures at the testing site. All procedures involving live animals were approved by the Institutional Animal Care and Use Committee (IACUC). Crown Bio, Inc., is an AAALAC-accredited institution.

At euthanasia, blood was obtained by cardiac puncture and used to assess liver enzyme (alanine aminotransferase [ALT] and aspartate aminotransferase [AST]) levels as a measure of injury and triglyceride (TG) levels as an indicator of metabolic function. Livers were removed and weighed. After gross examination, a piece of tissue from the left lobe was immediately frozen in liquid nitrogen and used for proteomic analysis. Another tissue piece from the left lobe was fixed in 10% buffered formalin and used for histology.

### AST, ALT and Triglyceride Assessment

Serum levels of AST, ALT and triglycerides were assessed using a Beckman chemical analyzer. All blood chemistry measurements were made following a standard operating procedure at Crown Bioscience.

### Histology and Assessment Scoring

Formalin-fixed liver tissue samples were processed for histology and 3–4 μm-thick sections were stained with hematoxylin and eosin (H&E) and picrosirius red (PSR), independently. H&E-stained tissue sections were evaluated under light microscopy for evidence of steatosis, ballooning hepatocyte degeneration and inflammation using the Kleiner scoring system (35). Kleiner et al have described the standardized histological scoring assessments of these NASH-related parameters in liver sections (35). These scoring methods have previously been applied to the histological scoring of mouse models of steatohepatitis by others (36, 37) and by us (20). Under this scoring system, a score of (0–3) was given for steatosis and inflammation separately, and a score of (0–2) for ballooning degeneration of hepatocytes. From these evaluations, a NAFLD activity score (NAS) was calculated by adding these individual scores. Finally, PSR-stained liver sections were used to assess collagen deposition and scored using a scale from 0–4 as defined by Kleiner et al. (35).

After microscopic evaluation, slides were digitized using the Aperio AT2 whole slide scanner (Leica Biosystems) at 40x with a resolution of 0.5 μm per pixel with a 20x objective. The scanned images were housed on a server and accessed using Leica Aperio eSlide Manager (Version 12.3.2.5030), a digital pathology management software. These digitized histological sections were viewed and analyzed using Aperio ImageScope (Version 12.3.3.5048), a slide viewing software by Leica.

### Proteomic Assessment

Proteomic experiments were carried out in the Proteomics Resource Facility (PRF), a core laboratory in the Department of Pathology at the University of Michigan. For protein mass spectrometry analysis, we employed liver tissue from five mice from each group and assessed each separately. A cryopreserved tissue piece from the left lobe (of each mouse liver) was weighed and homogenized in Radioimmuno-Precipitation Assay (RIPA) - lysis and extraction buffer (Pierce, # 89901; ThermoFisher Scientific) for protein isolation, as described in our previous publications (38–40). Fifty micrograms of sample protein from each liver were digested separately with trypsin and individual samples labeled with one of 11 isobaric mass tags following the manufacturer's protocol. Tandem Mass Tag (TMT) 11plex Isobaric Label Reagent Set (ThermoFisher Scientific) was utilized for this application. After labeling, equal amounts of sample (peptide) from each liver sample were mixed together. In order to achieve in-depth characterization of the proteome, the labeled peptides were fractionated using 2D-LC (basic pH reverse-phase separation followed by acidic pH reverse-phase) and analyzed on a high-resolution, tribrid mass spectrometer (Orbitrap Fusion Tribrid, ThermoFisher Scientific) using conditions optimized at the PRF. MultiNotch MS3 analysis (41) was used to accurately quantify identified proteins/peptides. Data analysis was performed using Proteome Discoverer (v 2.4, ThermoFisher Scientific). MS2 spectra were searched against UniProt mouse protein database (17,011 sequences as reviewed entries; downloaded on 05/06/2020) using the following search parameters: MS1 and MS2 tolerance were set to 10 ppm and 0.6 Da, respectively; carbamidomethylation of cysteines (57.02146 Da) and TMT labeling of lysine and N-termini of peptides (229.16293 Da) were considered static modifications; oxidation of methionine (15.9949 Da) and deamidation of asparagine and glutamine (0.98401 Da) were considered variable. Identified proteins and peptides were filtered to retain only those that passed ≤ 2% false discovery rate (FDR) threshold of detection. Quantitation was performed using high-quality MS3 spectra (Average signal-to-noise ratio of 10 and <40% isolation interference). Differential protein expression in each treatment cohort was normalized to the high-fat alone cohort. Protein names retrieved from UniProt.org, and Reactome V78 (reactome.org) was used for pathway enrichment analyses (42) for species *Mus musculus*. STRING database-v11 (string-db.org) was used to detect protein-protein interactions and additional enrichment analyses provide information related to cellular components, molecular functions, and biological processes by Gene Ontology (GO) annotation. It also offered WikiPathways and Kyoto Encyclopedia of Genes and Genomes (KEGG) databases to curate pathways. Only proteins with a ≤ 2% FDR confidence of detection were included in the analyses. The individual differential protein expression was established by calculating the abundance ratio of normalized abundances of each intervention sample to the high-fat alone samples. For the group analysis, individual data points were merged by groups to obtain means and standard deviations. The initial analysis was an unbiased search of differentially-expressed proteins and significantly altered proteins. Subsequently, we searched the database, specifically, for changes in the expression of keratins and other differentiation-related proteins. Mass spectrometry-based proteomics data were placed in a data repository known as ProteomeXchange Consortium via the PRIDE partner repository with the dataset identifier PXD030954.

### Statistical Analysis

Group means and standard deviations were obtained for discrete gross and histochemical features as well as for individual biochemical values (ALT, AST and TG) and individual protein values obtained in the proteomic analysis. Data generated in this way were analyzed by analysis of variance (ANOVA) followed by unpaired *t*-test (two-tailed) for comparison using GraphPad Prism, version 9. Pathways enrichment data reflect Reactome-generated *p*-values based on the number of entities identified in a given pathway as compared to total proteins responsible for that pathway. The significance (*p*-value) is calculated by the overrepresentation analysis (hypergeometric distribution). For STRING enrichment analysis, the whole genome statistical background was assumed and FDR stringency was ≤ 1%. Data were considered significant at *p* < 0.05.

## Results

### Body Weight, Liver Weight Comparisons, Liver Enzyme Levels, and Triglycerides

**Figure 1:**
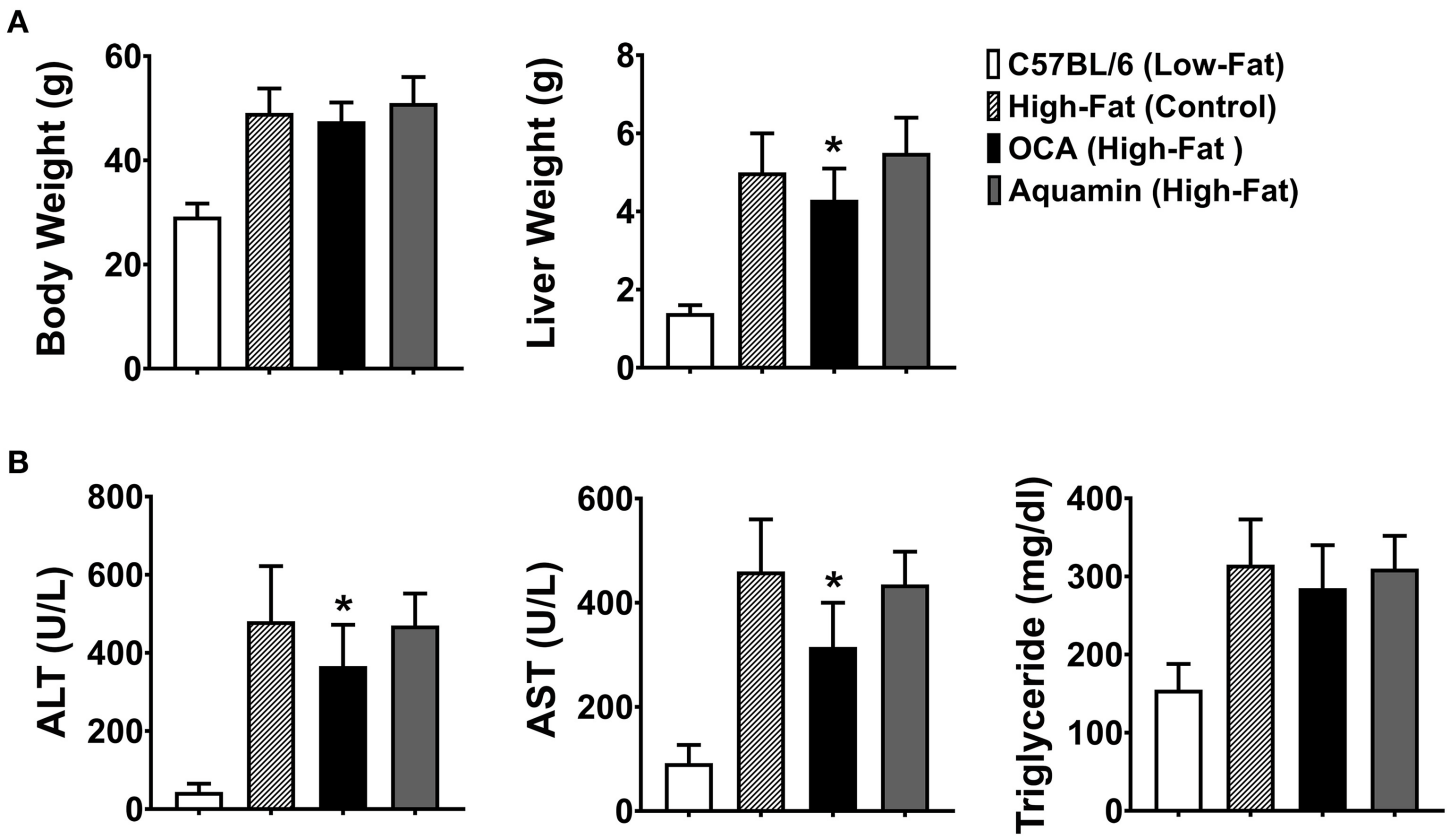
Whole-body weight, liver weight and biochemical markers. **(A)** Whole-body weight and liver weight were obtained for each animal at euthanasia. **(B)** Blood was obtained by cardiac puncture at euthanasia and assessed for ALT, AST, and triglyceride levels. Values shown are means and standard deviations based on nine animals per group. Statistical significance was assessed by ANOVA followed by a two-tailed *t*-test. Asterisks (*) indicate statistical difference from high-fat control at *p* < 0.05. With all five parameters, low-fat control values were statistically different from high-fat control at *p* < 0.05.

Animal weights and liver weights were assessed at euthanasia (Figure 1A). As expected, there were large differences between mice fed the low-fat diet and those on the high-fat diet irrespective of intervention. Inclusion of Aquamin® along with high-fat feeding had no effect on either total body weight or liver weight, but there was a small reduction in both parameters in the cohort of mice receiving OCA as compared to control mice on the high-fat diet. Liver enzyme (ALT and AST) levels and triglyceride levels are shown in Figure 1B. Levels of both ALT and AST increased substantially with high-fat feeding as did triglyceride levels. Both liver enzymes were also increased in mice receiving either Aquamin® or OCA along with high-fat feeding. With OCA, there was a modest reduction compared to levels seen in untreated mice on the same diet. Differences between the interventions and control (high-fat diet-fed mice) were not statistically different for either ALT or AST but all three high-fat groups were different from the low-fat control group. Serum triglyceride levels (evidence of metabolic dysfunction) were significantly elevated with high-fat feeding but neither Aquamin® nor OCA had a significant effect on this parameter.

### Histological Features

**Figure 2:**
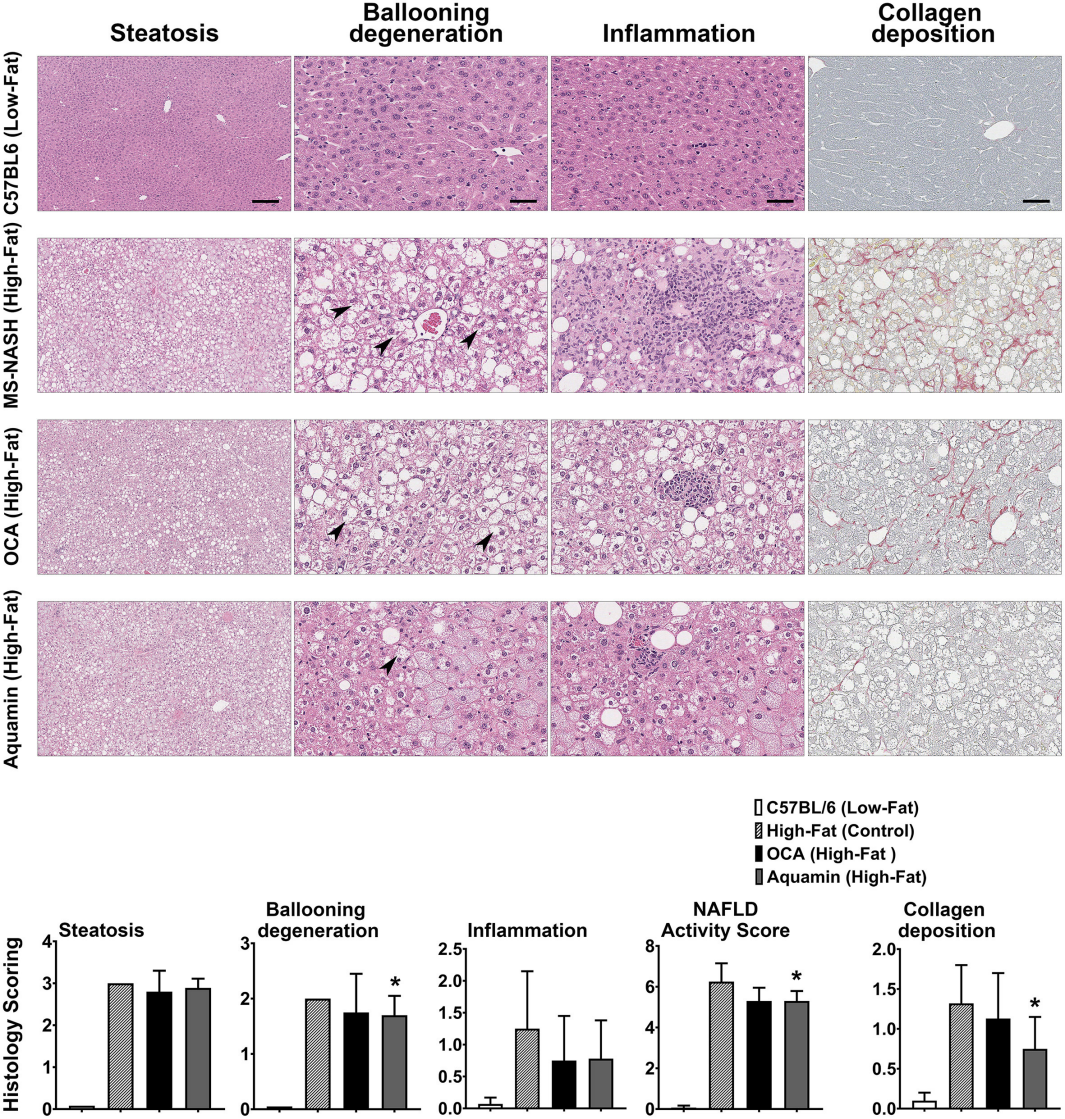
Histological features. H&E-stained liver sections from all four groups were evaluated for steatosis, ballooning hepatocyte degeneration and inflammation by two trained individuals who did not know the treatment group from which the section was obtained. Arrows draw attention to the distended hepatocytes with clear cytoplasm and non-displaced nuclei representing ballooning degeneration. Inflammatory foci are visible in inflammation panels (of high-fat mice). The scoring of each parameter was conducted as described in the Materials and Methods Section and followed the Kleiner system (35). The NAFLD activity score (NAS) is a summation of the three individual parameters (steatosis, ballooning hepatocyte degeneration and inflammation), calculated in each mouse. Picrosirius red (PSR)-stained liver sections from all four groups were evaluated for collagen deposition. Values shown are means and standard deviations based on nine animals per group. Statistical significance was assessed by ANOVA followed by a two-tailed *t*-test. Asterisks (*) indicate statistical difference from high-fat control at *p* < 0.05. For steatosis panels, scale bar = 200 μm, for ballooning degeneration, inflammation and collagen deposition (PSR) panels, scale bar = 50 μm.

Sections of formalin-fixed liver tissue were stained with hematoxylin and eosin and evaluated for steatosis, ballooning hepatocyte degeneration and inflammation. Additional sections were stained with PSR and assessed for collagen deposition. Data and histological representations of all four groups are shown in Figure 2. Steatosis was virtually undetectable in mice on the low-fat control diet but widespread in every animal on the high-fat diet after 16 weeks of feeding. Neither Aquamin® nor OCA reduced steatosis significantly compared to the high-fat control, but both interventions reduced ballooning degeneration and inflammation to some extent. When scores from the three parameters (steatosis, ballooning degeneration and inflammation) were added together (as in citation 35) to give a NAFLD activity score, both Aquamin® and OCA were different from high-fat alone. However, the degree of elevation was small to begin with (especially with inflammation) and the overall significance of the modest reduction is difficult to gauge. PSR staining demonstrated collagen deposition within the liver parenchyma of mice on the high-fat diet while sections from low-fat control mice did not show staining except around vascular elements and in relation to the capsule. Some reduction in the collagen deposition score was noted with both interventions. With Aquamin®, the reduction in collagen deposition reached a level of statistical significance. However, as with inflammation, the overall PSR staining score was low (<1.5 out of 4) after 16 weeks on the high-fat diet.

### Proteomic Analysis: Effects of Low-Fat Diet, Aquamin® and OCA Compared to High-Fat Alone

**Figure 3:**
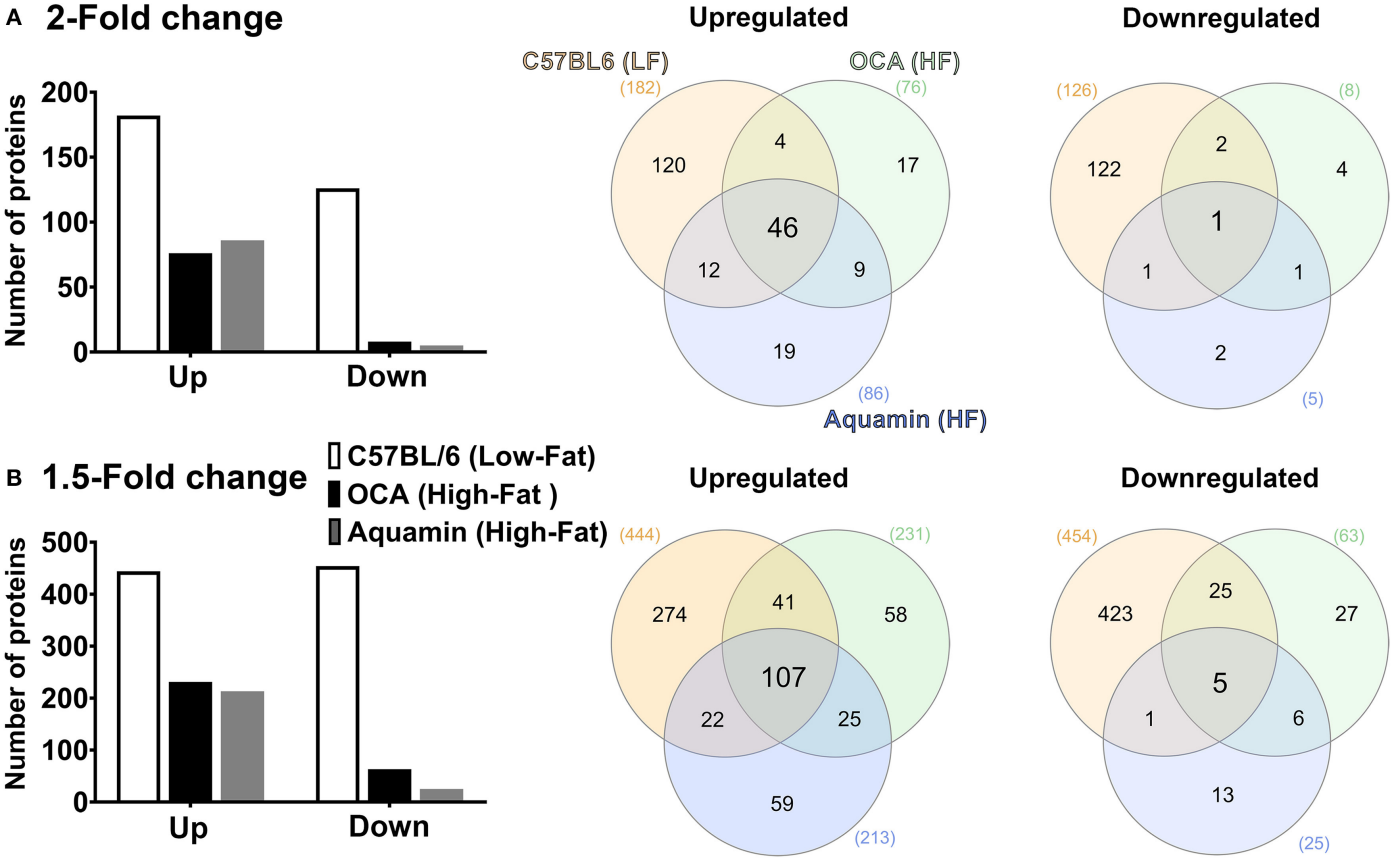
Proteomic analysis. Lysates were prepared from liver tissue of individual mice (five per group) and subjected to Tandem Mass Tag (TMT) labeling / mass spectroscopy as described in the Materials and Methods Section. Data from individual animals were merged by treatment group for analysis. Left panels: Abundance ratios of all proteins were calculated by comparing the normalized abundance of each group to the high-fat control group. The upper panel indicates the number of proteins that were increased or decreased by 2-fold **(A)** or greater (with ≤ 2% FDR) and the lower panel indicates the number of proteins that were increased or decreased by 1.5-fold **(B)** or greater (with ≤ 2% FDR). Right panels: Venn diagrams showing the overlap in the number of proteins altered (increased or decreased) by an average of 2-fold **(A)** or greater (upper panel) or 1.5-fold **(B)** or greater (lower panel) among the three treatment groups relative to the high-fat control and unique to each group.

The number of upregulated and downregulated proteins in C57BL/6 mice on the low-fat diet (relative to high-fat control mice) and the number of up- and downregulated proteins with Aquamin® or OCA in comparison to the high-fat control mice is presented as Figure 3A (2-fold cutoff) and Figure 3B (1.5-fold cutoff). It can be seen from the bar graphs that the low-fat control diet was responsible for a substantial number of protein changes. With the 2-fold cutoff and ≤ 2% FDR, 182 proteins were upregulated, and 126 proteins were downregulated with the low-fat control diet. With Aquamin® and OCA, a total of 86 and 76 proteins, respectively, were upregulated to the same extent, while only five and eight, respectively, were downregulated. When the less stringent cut-off (1.5-fold) was used, more proteins were altered with each intervention but the trends were the same.

Venn diagrams (Figures 3A,B) show the overlap between protein alterations with low-fat feeding and the other two interventions. Among the proteins elevated with either Aquamin® or OCA, ~50% (at either cut-off) were in common with proteins upregulated in the low-fat control diet. In contrast, the number of downregulated proteins with either intervention that overlapped with proteins downregulated with low-fat feeding was much lower on a percentage basis. This was seen with either cutoff but was more extreme with a 2-fold cutoff. Overall, 55 upregulated proteins were common between the two interventions (OCA and Aquamin®), while only two downregulated proteins were common between these two groups. Supplementary Tables 2, 3 provide lists of upregulated and downregulated proteins common at 2-fold among three groups (low-fat, OCA and Aquamin® on the high-fat diet) across five mice per group.

### Proteomic Analysis: Individual Protein Changes in Response to Aquamin®

**Figure 4:**
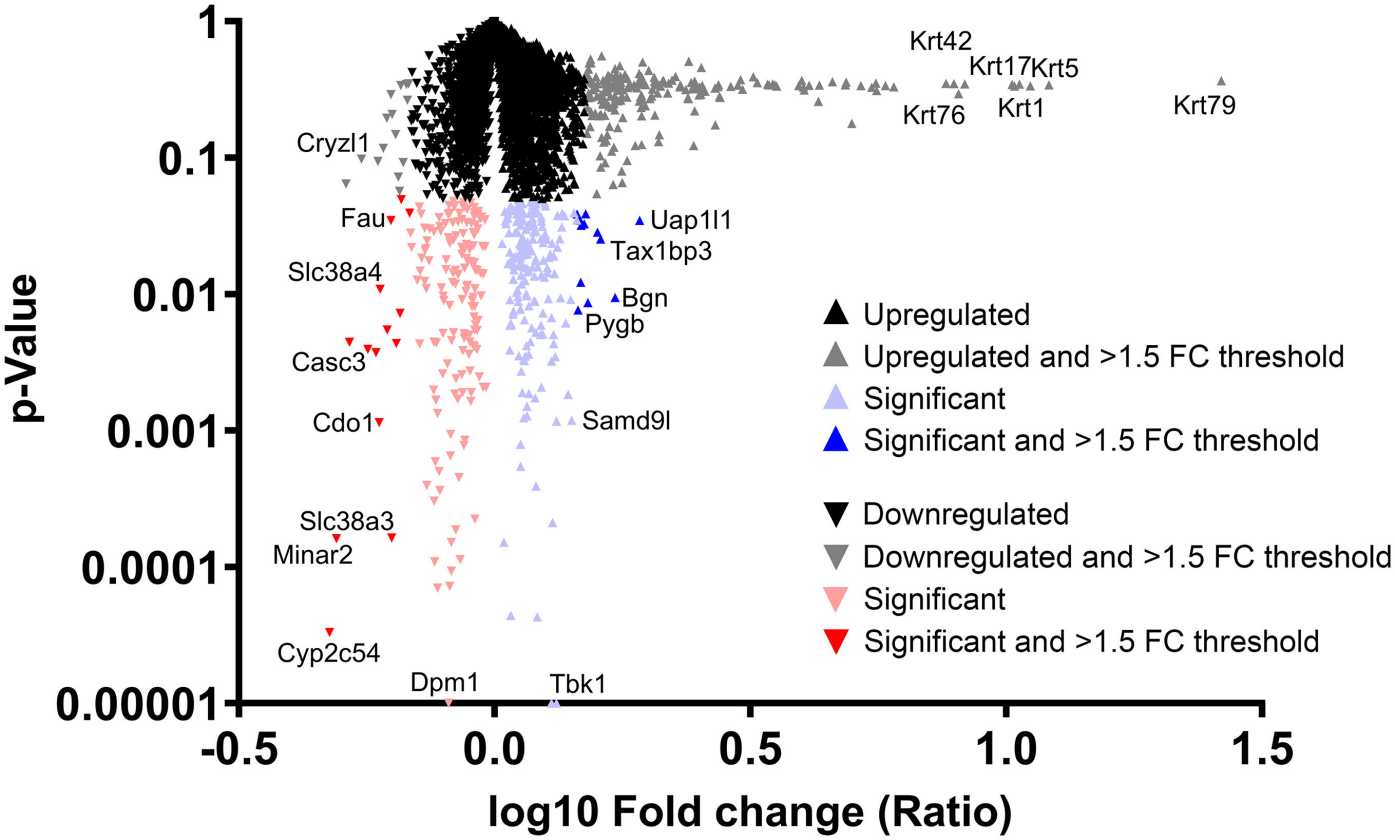
Distribution of proteins upregulated or downregulated in response to Aquamin® compared to high-fat control. Value for each protein (group average) in the high-fat control was set to 1.0 and values in the Aquamin® group were compared to this. The x-axis shows the fold-change (log10) of individual proteins and the y-axis reflects individual protein *p*-values (*N* = 5 individual liver samples per group). The mass of proteins shown in black represent proteins that were <1.5-fold different from control (up or down) and not statistically significant. Proteins depicted with gray were different from control by 1.5-fold or greater but not statistically different. Red triangles represent down-regulated proteins and blue triangles represent up-regulated proteins that were statistically significant as compared to control. Bold color (red or blue) represents proteins that were both statistically different from control and different from control by 1.5-fold or greater. Certain individual proteins are identified by UniProt protein (gene) symbol.

Figure 4 shows the fold-change distribution of individual proteins responsive to Aquamin® in the presence of a high-fat diet. Upregulated proteins that met the criterion of statistical significance at *p* < 0.05 are shown in blue while downregulated proteins are shown in red. The bold colors (blue and red) indicate proteins that were statistically different and also at least 1.5-fold up- or downregulated.

Table 1A identifies the upregulated proteins that met the dual criteria (statistical difference from the high-fat control at *p* < 0.05 and at least 1.5-fold increase) in response to Aquamin®. Supplementary Table 4 provides a comprehensive list of upregulated proteins that met the criterion of statistical difference from control, irrespective of fold-change. While only a small number of proteins (12 in total) met both criteria, a total of 183 upregulated proteins were statistically increased. Reactome pathway analysis was utilized as a way to help delineate how altered protein expression in response to Aquamin® influences biological function. As can be seen from the altered pathways, the upregulated proteins (for example, Apolipoprotein A-I [Apoa1]) impacted *lipid metabolism* and *trafficking of lipids* (Table 1B). Apoa1 was significantly upregulated with Aquamin® and it did not change in OCA and low-fat groups. Apoa1 is a major component of high-density lipoprotein (HDL) and may have a protective role against liver cancer (43). Supplementary Table 5 lists top pathways affected by the altered (significantly upregulated) proteins presented in Supplementary Table 4.

**Table 1A:** Significantly upregulated proteins with Aquamin in high-fat mice.

		**MS-NASH**		**C57BL6**
**Proteins**	**Genes**	**Aquamin**	**OCA**	**(Low-Fat)**
UDP-N-acetylhexosamine pyrophosphorylase-like protein 1	Uap1l1	1.9 ± 0.8*	0.9 ± 0.4	1.1 ± 0.2
Biglycan	Bgn	1.7 ± 0.5*	1.5 ± 0.6	0.8 ± 0.3
Tax1-binding protein 3	Tax1bp3	1.6 ± 0.5*	1.6 ± 1.1	1.1 ± 0.5
Succinyl-CoA:3-ketoacid coenzyme A transferase 1, mitochondrial	Oxct1	1.6 ± 0.3*	1.4 ± 0.2*	1.2 ± 0.4
Glycogen phosphorylase, brain form	Pygb	1.5 ± 0.3*	0.9 ± 0.2	0.6 ± 0.1
Acyl-coenzyme A thioesterase 9, mitochondrial	Acot9	1.5 ± 0.4*	1.3 ± 0.6	0.8 ± 0.6
CD166 antigen	Alcam	1.5 ± 0.4*	1.3 ± 0.5	1.2 ± 0.2*
Erythrocyte band seven integral membrane protein	Stom	1.5 ± 0.4*	1.2 ± 0.5	0.9 ± 0.5
RNA-binding protein 3	Rbm3	1.5 ± 0.3*	1.4 ± 0.4*	1.2 ± 0.3
Angiotensinogen	Agt	1.5 ± 0.4*	1.1 ± 0.3	1.5 ± 0.4*
Apolipoprotein A-I	Apoa1	1.5 ± 0.3*	1.0 ± 0.3	0.8 ± 0.2
Heparin cofactor 2	Serpind1	1.5 ± 0.4*	1.2 ± 0.6	1.1 ± 0.4

These values (in this table) represent average (± standard deviation) fold-change of abundance ratios for each altered (upregulated) protein compared to the high-fat control group (MS-NASH mice on a high-fat diet) with a 1.5-fold change threshold in response to Aquamin intervention and are significant with a p-value <0.05 (*). For each upregulated protein with Aquamin, corresponding values from the other two groups are shown for comparison. These liver samples (from five mice in each group) were individually assessed by TMT-based differential proteomic expression and data were merged to get averages. Protein FDR Confidence for all proteins was ≤ 1%. Asterisks indicate statistical significance as compared to High-fat control at p <0.05. FDR, False Discovery Rate. These data are also presented in Figure 4.

**Table 1B:** Top pathways associated with upregulated proteins (listed in Table 1A) altered with Aquamin.

**Pathway name**	**Entities *p*-value**	**Mapped entities**
HDL clearance	0.004	Apoa1
Scavenging by Class B Receptors	0.005	Apoa1
Utilization of Ketone Bodies	0.005	Oxct1
RHO GTPases Activate Rhotekin and Rhophilins	0.008	Tax1bp3
HDL assembly	0.009	Apoa1
Post-translational protein phosphorylation	0.010	Apoa1;Serpind1
Regulation of IGF transport and uptake by IGFBPs	0.011	Apoa1;Serpind1
HDL remodeling	0.012	Apoa1
Chylomicron remodeling	0.013	Apoa1
Chylomicron assembly	0.013	Apoa1
Scavenging by Class A Receptors	0.013	Apoa1
Ketone body metabolism	0.013	Oxct1
Dermatan sulfate biosynthesis	0.014	Bgn
Heme signaling	0.014	Apoa1
CS/DS degradation	0.018	Bgn
Metabolism of Angiotensinogen to Angiotensins	0.021	Agt
ABC transporters in lipid homeostasis	0.022	Apoa1
Plasma lipoprotein assembly	0.024	Apoa1
Chondroitin sulfate biosynthesis	0.026	Bgn
Common Pathway of Fibrin Clot Formation	0.027	Serpind1
Intrinsic Pathway of Fibrin Clot Formation	0.030	Serpind1
A tetrasaccharide linker sequence is required for GAG synthesis	0.034	Bgn
Plasma lipoprotein remodeling	0.035	Apoa1
Plasma lipoprotein clearance	0.039	Apoa1

*The pathways listed here are altered by the significantly upregulated proteins with the intervention “Aquamin” presented in Table 1A. Reactome (v78) was used to generate the pathway analysis report for species Mus musculus. The significance (p-value) is calculated by the overrepresentation analysis (hypergeometric distribution). IGF, Insulin-like Growth Factor; IGFBPs, Insulin-like growth factor binding proteins*.

Aquamin®-responsive proteins that met the dual criteria of 1.5-fold decrease and statistical significance are shown in Table 1C and those that were statistically-different (and downregulated) from high-fat control, regardless of fold change, are shown in Supplementary Table 6. A total of 14 proteins met both criteria while a total of 130 were statistically different from the high-fat control, independent of fold-change. As with upregulated proteins, Reactome pathway analysis was used to help identify potential mechanisms of action (Table 1D). Proteins whose expression was reduced by Aquamin® impact, primarily, *amino acid metabolism*, although *purine and pyrimidine metabolism* also appear to be targets. Among the downregulated proteins were multiple cytochrome P450 family members (10 in all) including three (i.e., 2C54, 2C50, 2C29) that were among the most highly downregulated (Supplementary Table 6). Cytochrome P450 family members are highly-expressed in the liver and serve to metabolize and detoxify numerous chemicals, including fats (44). During detoxification, cytochrome P450 enzymes can cause oxidative stress and may play a role in cellular injury, which can lead to NASH (45). Supplementary Table 7 lists top pathways affected by all of the significantly downregulated proteins presented in Supplementary Table 6. *Amino acid metabolism, bile acid and bile salt metabolism* and *recycling of bile acids and salts* are among the significantly downregulated pathways identified.

**Table 1C:** Significantly downregulated proteins with Aquamin in high-fat mice.

		**MS-NASH**		**C57BL6**
**Proteins**	**Genes**	**Aquamin**	**OCA**	**(Low-Fat)**
Cytochrome P450 2C54	Cyp2c54	0.48 ± 0.14*	0.61 ± 0.19*	2.72 ± 0.49
Major intrinsically disordered NOTCH2-binding receptor 1-like homolog^#^	Minar2	0.49 ± 0.12*	0.45 ± 0.18*	1.06 ± 0.23
Alpha-1-antitrypsin 1–5	Serpina1e	0.52 ± 0.27*	0.70 ± 0.35	37.05 ± 11.87
Protein CASC3	Casc3	0.57 ± 0.13*	0.55 ± 0.16*	0.60 ± 0.22*
Major urinary protein 2	Mup2	0.59 ± 0.23*	0.44 ± 0.19*	10.40 ± 5.30
Cysteine dioxygenase type 1	Cdo1	0.59 ± 0.18*	0.54 ± 0.11*	1.94 ± 0.66
Sodium-coupled neutral amino acid transporter 4	Slc38a4	0.60 ± 0.27*	0.62 ± 0.27*	1.30 ± 0.37
Ferritin light chain 1	Ftl1	0.62 ± 0.23*	0.74 ± 0.27	1.18 ± 0.80
40S ribosomal protein S30	Fau	0.63 ± 0.33*	0.71 ± 0.26*	0.80 ± 0.42
Sodium-coupled neutral amino acid transporter 3	Slc38a3	0.63 ± 0.13*	0.71 ± 0.18*	2.60 ± 1.10
2-oxo-4-hydroxy-4-carboxy-5-ureidoimidazoline decarboxylase	Urad	0.64 ± 0.20*	0.94 ± 0.20	3.19 ± 2.07
Cytochrome P450 2C50	Cyp2c50	0.65 ± 0.22*	0.79 ± 0.40	4.82 ± 2.53
5'-nucleotidase	Nt5e	0.66 ± 0.28*	0.67 ± 0.19*	1.33 ± 0.30
Ileal sodium/bile acid cotransporter	Slc10a2	0.67 ± 0.23*	0.77 ± 0.22	0.54 ± 0.27*

These values (in this table) represent average (± standard deviation) fold-change of abundance ratios for each altered (downregulated) protein compared to the high-fat control group (MS-NASH mice on a high-fat) with a 1.5-fold change threshold in response to Aquamin intervention and are significant with a p-value <0.05 (*). For each downregulated protein with Aquamin, corresponding values from the other two groups are shown for comparison. These liver samples (from five mice in each group) were individually assessed by TMT-based differential proteomic expression and data were merged to get averages. Protein FDR Confidence for all proteins was ≤ 1% except for 1 downregulated protein^#^ ( ≤ 2%). Asterisks indicate statistical significance as compared to High-fat control at p < 0.05. FDR, False Discovery Rate. These data are also presented in Figure 4.

**Table 1D:** Top pathways associated with downregulated proteins (listed in Table 1C) altered with Aquamin.

**Pathway name**	**Entities *p*-value**	**Mapped entities**
Amino acid transport across the plasma membrane	0.001	Slc38a3;Slc38a4
Transport of inorganic cations/anions and amino acids/oligopeptides	0.011	Slc38a3;Slc38a4
Pyrimidine catabolism	0.017	Nt5e
Degradation of cysteine and homocysteine	0.018	Cdo1
Recycling of bile acids and salts	0.026	Slc10a2
Purine catabolism	0.027	Nt5e
Sulfur amino acid metabolism	0.035	Cdo1
Nicotinate metabolism	0.047	Nt5e
SLC-mediated transmembrane transport	0.050	Slc38a3;Slc38a4
Nucleotide catabolism	0.050	Nt5e

*The pathways listed here are altered by the significantly downregulated proteins with the intervention “Aquamin” presented in Table 1C. Reactome (v78) was used to generate the pathway analysis report for species Mus musculus. The significance (p-value) is calculated by the overrepresentation analysis (hypergeometric distribution)*.

Tables 1A,C also show for comparison how the Aquamin®-sensitive proteins responded to low-fat feeding and to OCA. It can be seen that while there was overlap between Aquamin® and OCA (as shown in Figure 3) in many of the highly upregulated proteins, the response to OCA was not as robust as the response to Aquamin®. Fold-change was smaller and only two proteins were statistically different from high-fat control, although both interventions were introduced in the presence of the same diet and the same mouse strain. In low-fat mice, only one protein met the same criteria (Table 1A). In regard to downregulated moieties, of the 14 Aquamin®-sensitive proteins that met the dual criteria, nine were also responsive to OCA by the same criteria. In contrast, there was little overlap between Aquamin®-responsiveness and response to the low-fat diet. In fact, many of the protein changes were in the opposite direction (Table 1C).

Supplementary Tables 8, 9 present proteomic enrichment data curated by the STRING database with 183 significantly upregulated proteins and 130 significantly downregulated proteins, respectively, in response to Aquamin®. These datasheets provide information related to biological processes, molecular functions and cellular components along with additional pathways curated by KEGG and Wiki (as part of STRING enrichment analysis). Among these, inflammation-related pathways were significantly downregulated with Aquamin® (Supplementary Table 9).

**Figure 5:**
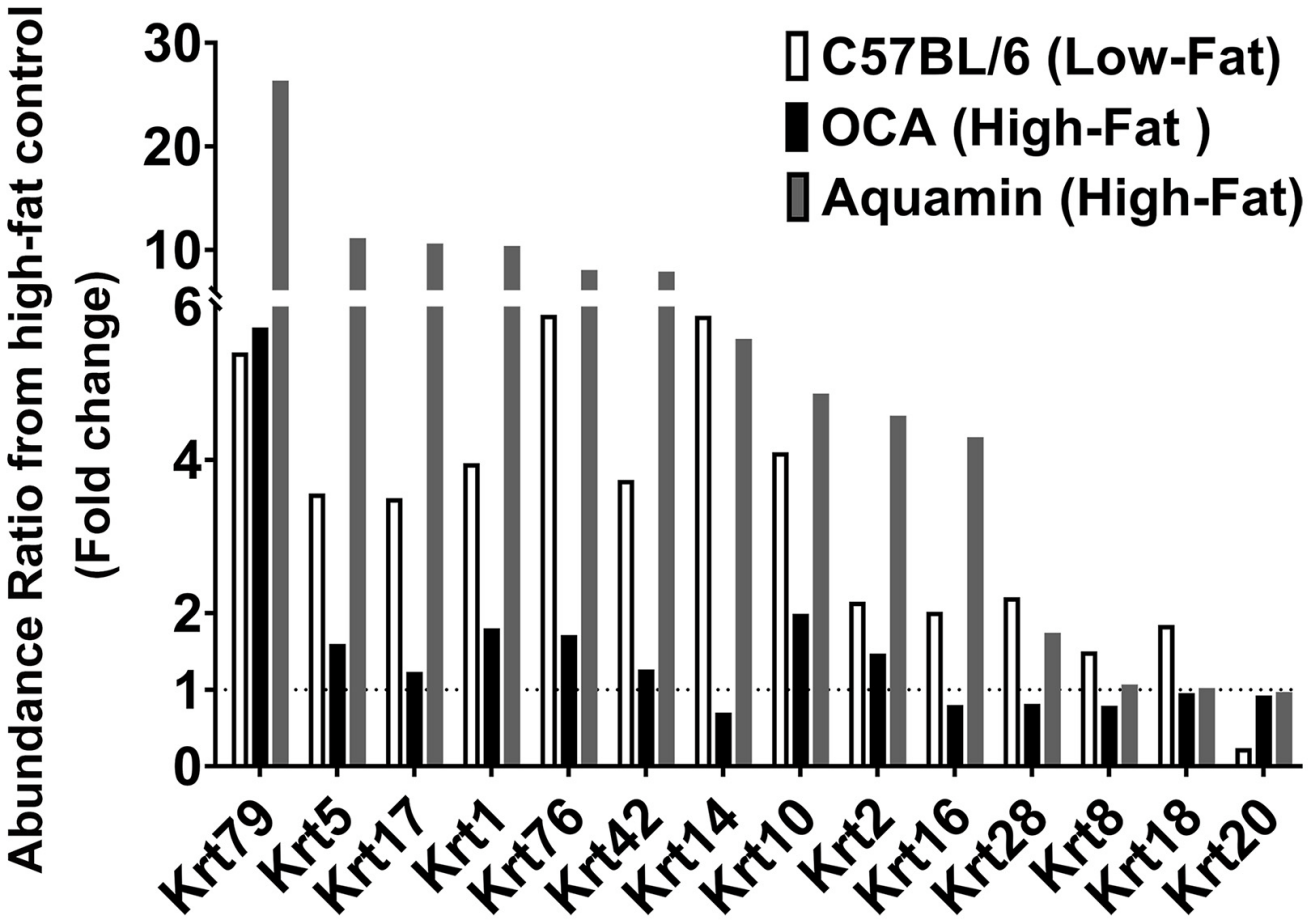
Keratins upregulated in response to Aquamin®. Values represent average fold-change from high-fat control based on *n* = 5 liver samples per treatment group. Corresponding values for response to a low-fat diet and response to OCA are shown for comparison.

After analyzing data based on statistical significance, the proteomic database was searched for the most highly up- and downregulated Aquamin®-responsive proteins based on fold-change independent of statistical significance. Table 2 presents a list of the most highly upregulated proteins (2-fold or greater) with Aquamin®. As a group, epithelial cell keratins were prominent in the protein list. Of the top 22 most highly upregulated proteins, 10 were keratins (Table 2). Also, nine of these keratins were common at a 2-fold-change with the low-fat group (Supplementary Table 2). Of interest, the major keratins found in the liver – i.e., keratin 8, and 18 (46) – were not altered (up or down) with Aquamin® (Figure 5). Keratins are structural components of epithelial cells and play key roles in differentiation, morphogenesis, barrier formation and tissue integrity. They are also important signaling intermediates (47). How each of the keratins identified here functions in the liver, specifically, is not known. The effects of low-fat feeding and the effects of treatment with OCA on these same proteins are included in Table 2 (complete protein list) and Figure 5 (keratins). As can be seen, some of the same keratins were increased with low-fat feeding but the degree of upregulation was less than that observed with Aquamin®. While the low-fat diet does not provide the same trace elements as does Aquamin®, D5008 chow contains some minerals as part of its formulation. Thus, it is not unreasonable to observe some change in the keratin expression profile in livers of mice on the low-fat diet. In contrast, with the exception of keratin 79, upregulation of individual keratins with OCA was almost non-existent.

**Table 2:** Upregulated proteins by an unbiased proteomic screening with Aquamin in high-fat mice.

		**MS-NASH**		**C57BL6**
**Proteins**	**Genes**	**Aquamin**	**OCA**	**Low-Fat**
Keratin, type II cytoskeletal 79	Krt79	26.34 ± 43.11	5.73 ± 7.97	5.40 ± 4.07
Nuclear transport factor 2	Nutf2	12.12 ± 24.59	10.29 ± 20.76	8.16 ± 16.12
Keratin, type II cytoskeletal 5	Krt5	11.14 ± 22.14	1.60 ± 2.45	3.56 ± 4.11
Keratin, type I cytoskeletal 17	Krt17	10.62 ± 21.47	1.23 ± 1.59	3.50 ± 2.69
Keratin, type II cytoskeletal 1	Krt1	10.38 ± 20.44	1.80 ± 2.51	3.96 ± 3.36
Fucose mutarotase	Fuom	10.28 ± 20.61	10.03 ± 19.94	8.72 ± 17.40
SH3 domain-containing protein 21^#^	Sh3d21	8.30 ± 16.34	5.43 ± 10.12	6.05 ± 10.62
Keratin, type II cytoskeletal 2 oral	Krt76	8.07 ± 14.08	1.71 ± 2.42	5.89 ± 7.01
Keratin, type I cytoskeletal 42	Krt42	7.91 ± 13.57	1.26 ± 1.63	3.74 ± 3.98
Glycine cleavage system H protein, mitochondrial	Gcsh	7.63 ± 14.93	9.05 ± 17.88	11.09 ± 21.41
Pigment epithelium-derived factor	Serpinf1	6.03 ± 10.85	5.58 ± 10.41	3.34 ± 5.88
Tubulin beta-5 chain	Tubb5	5.81 ± 10.51	5.06 ± 9.36	4.90 ± 9.25
Keratin, type I cytoskeletal 14	Krt14	5.58 ± 9.45	0.70 ± 0.43	5.88 ± 7.13
Transmembrane protein 14C	Tmem14c	5.56 ± 10.29	4.82 ± 8.80	5.97 ± 10.43
Ras-related protein R-Ras	Rras	5.46 ± 9.57	4.75 ± 8.44	6.32 ± 11.74
Ras-related protein Rap-1A^#^	Rap1a	5.20 ± 9.18	5.83 ± 10.73	5.66 ± 10.21
Protein PAT1 homolog 1	Patl1	4.99 ± 4.23	2.74 ± 2.40	3.09 ± 2.72
Keratin, type I cytoskeletal 10	Krt10	4.86 ± 8.83	1.99 ± 3.15	4.10 ± 6.43
Tubulin beta-4A chain	Tubb4a	4.85 ± 8.51	4.73 ± 8.30	3.76 ± 6.90
Keratin, type II cytoskeletal 2 epidermal	Krt2	4.58 ± 8.23	1.47 ± 1.86	2.15 ± 1.84
60S ribosomal protein L36	Rpl36	4.32 ± 7.31	4.95 ± 8.52	4.35 ± 8.31
Keratin, type I cytoskeletal 16	Krt16	4.30 ± 6.06	0.80 ± 0.58	2.02 ± 1.83
IgG receptor FcRn large subunit p51	Fcgrt	4.12 ± 6.83	5.74 ± 10.39	4.17 ± 6.07
U8 snoRNA-decapping enzyme	Nudt16	4.03 ± 5.49	3.02 ± 4.24	3.26 ± 4.15
Ig kappa chain V-V region MOPC 149	Igkv12-44	4.02 ± 4.71	2.06 ± 2.70	1.52 ± 2.10
Alpha-1-acid glycoprotein 1	Orm1	3.98 ± 6.98	2.93 ± 5.05	6.12 ± 9.25
Endophilin-B1	Sh3glb1	3.93 ± 6.42	3.47 ± 5.80	3.27 ± 5.18
Proteasome subunit beta type-3	Psmb3	3.56 ± 5.56	3.50 ± 5.41	3.27 ± 4.30
60S acidic ribosomal protein P1	Rplp1	3.53 ± 5.59	3.61 ± 5.97	4.84 ± 8.27
Coatomer subunit epsilon	Cope	3.50 ± 5.59	2.78 ± 4.10	3.27 ± 4.74
Copper transport protein ATOX1	Atox1	3.44 ± 5.40	2.84 ± 4.30	1.64 ± 2.28
Translation initiation factor eIF-2B subunit epsilon	Eif2b5	3.42 ± 4.73	1.63 ± 1.08	1.76 ± 1.80
Galectin-related protein	Lgalsl	3.26 ± 5.14	3.52 ± 5.77	2.86 ± 4.63
Putative RNA-binding protein Luc7-like 1	Luc7l	3.20 ± 4.78	2.56 ± 3.40	2.67 ± 3.04
5'-AMP-activated protein kinase subunit beta-1	Prkab1	3.05 ± 4.30	3.00 ± 3.74	1.92 ± 2.61
Splicing factor 3B subunit 4	Sf3b4	3.03 ± 4.20	3.57 ± 5.19	3.46 ± 4.27
2-hydroxyacyl-CoA lyase 2	Ilvbl	2.99 ± 4.38	2.82 ± 4.22	2.91 ± 4.43
Small nuclear ribonucleoprotein E	Snrpe	2.90 ± 4.12	2.22 ± 2.99	2.86 ± 3.61
Arylsulfatase A	Arsa	2.76 ± 3.37	1.06 ± 0.95	0.81 ± 0.40
Aldose reductase-related protein 2	Akr1b8	2.75 ± 3.39	2.25 ± 2.73	2.84 ± 3.75
Aldo-keto reductase family 1 member C18	Akr1c18	2.74 ± 3.86	2.31 ± 2.65	2.52 ± 2.51
H-2 class II histocompatibility antigen gamma chain	Cd74	2.73 ± 3.74	0.97 ± 0.77	0.47 ± 0.18
Ig gamma-2A chain C region secreted form	Ighg2a	2.70 ± 2.55	1.17 ± 0.49	0.72 ± 0.32
S-methylmethionine–homocysteine S-methyltransferase BHMT2	Bhmt2	2.70 ± 3.60	2.51 ± 3.26	2.01 ± 2.67
Glutaredoxin-1	Glrx	2.58 ± 3.23	2.85 ± 3.94	3.76 ± 5.34
LIM and senescent cell antigen-like-containing domain protein 1	Lims1	2.55 ± 3.39	1.55 ± 1.45	1.37 ± 1.22
Nuclear cap-binding protein subunit 1	Ncbp1	2.54 ± 3.24	2.41 ± 3.23	2.29 ± 2.99
NAD-dependent malic enzyme, mitochondrial	Me2	2.54 ± 3.24	1.95 ± 2.35	2.24 ± 2.73
Cytochrome b-245 heavy chain	Cybb	2.53 ± 3.03	1.82 ± 2.05	1.42 ± 1.62
Peptidyl-prolyl cis-trans isomerase NIMA-interacting 1	Pin1	2.52 ± 2.87	2.51 ± 2.90	2.43 ± 2.54
Signal transducer and activator of transcription 1	Stat1	2.51 ± 2.96	1.51 ± 1.49	0.66 ± 0.30
Protein quaking	Qki	2.49 ± 3.17	2.06 ± 2.46	1.99 ± 2.52
Cytochrome b-c1 complex subunit 10	Uqcr11	2.47 ± 3.24	2.45 ± 3.24	3.40 ± 4.36
Signal recognition particle 19 kDa protein	Srp19	2.46 ± 3.39	2.29 ± 3.07	3.08 ± 4.09
Fermitin family homolog 3	Fermt3	2.46 ± 2.45	1.51 ± 1.48	1.30 ± 1.15
AP-2 complex subunit sigma	Ap2s1	2.45 ± 3.06	2.05 ± 2.57	2.61 ± 3.59
Platelet-activating factor acetylhydrolase IB subunit gamma	Pafah1b3	2.45 ± 1.29	2.21 ± 1.34	1.80 ± 0.90
Neuroplastin	Nptn	2.44 ± 3.07	2.11 ± 2.67	2.33 ± 2.72
NADH-ubiquinone oxidoreductase chain 5	Mtnd5	2.42 ± 3.25	2.44 ± 3.18	2.55 ± 3.40
Guanylate-binding protein 1	Gbp1	2.40 ± 3.98	1.69 ± 2.15	0.58 ± 0.28
Carboxypeptidase B2	Cpb2	2.39 ± 2.63	2.29 ± 2.74	3.39 ± 4.66
Lysosomal acid lipase/cholesteryl ester hydrolase	Lipa	2.38 ± 2.64	1.68 ± 1.83	1.89 ± 2.16
[Protein ADP-ribosylarginine] hydrolase	Adprh	2.36 ± 2.84	2.05 ± 2.29	1.53 ± 1.69
cAMP-dependent protein kinase type I-alpha regulatory subunit	Prkar1a	2.31 ± 2.86	1.96 ± 2.45	1.76 ± 1.95
ADP-ribosylation factor 4	Arf4	2.31 ± 2.87	2.02 ± 2.64	1.84 ± 2.57
Tubulin beta-2A chain	Tubb2a	2.30 ± 2.80	2.26 ± 2.77	1.00 ± 1.29
COMM domain-containing protein 8	Commd8	2.26 ± 2.73	2.16 ± 1.99	2.63 ± 2.14
H-2 class II histocompatibility antigen, I-A beta chain	H2-Eb1	2.22 ± 2.52	1.26 ± 0.70	0.54 ± 0.33
Rho guanine nucleotide exchange factor 10-like protein	Arhgef10l	2.19 ± 2.68	1.90 ± 2.04	1.43 ± 1.52
TIP41-like protein	Tiprl	2.18 ± 2.36	2.36 ± 2.70	1.63 ± 1.88
Galactose-1-phosphate uridylyltransferase	Galt	2.14 ± 2.67	3.02 ± 4.19	2.84 ± 3.21
Acetyl-coenzyme A transporter 1	Slc33a1	2.13 ± 2.62	2.20 ± 2.51	2.04 ± 2.75
Nascent polypeptide-associated complex subunit alpha, muscle-specific	Naca	2.13 ± 2.58	2.15 ± 2.63	2.29 ± 2.60
Vascular non-inflammatory molecule 3	Vnn3	2.11 ± 2.42	1.75 ± 2.17	1.18 ± 1.38
Protein S100-A11	S100a11	2.10 ± 1.53	1.17 ± 1.17	0.51 ± 0.31
CD81 antigen	Cd81	2.10 ± 2.42	1.67 ± 2.05	2.36 ± 2.50
H-2 class II histocompatibility antigen, A-B alpha chain	H2-Aa	2.09 ± 1.00	1.33 ± 0.48	0.62 ± 0.13
Charged multivesicular body protein 1b-1	Chmp1b1	2.08 ± 2.17	1.81 ± 1.87	1.56 ± 1.35
STIP1 homology and U box-containing protein 1	Stub1	2.07 ± 2.40	2.22 ± 2.68	2.33 ± 2.80
Proteasome subunit beta type-8	Psmb8	2.06 ± 1.71	1.61 ± 1.06	1.10 ± 0.63
Acyl-CoA-binding protein	Dbi	2.05 ± 2.43	2.20 ± 3.02	2.05 ± 2.30
Serine hydrolase-like protein^#^	Serhl	2.05 ± 1.88	1.94 ± 1.62	2.10 ± 2.09
WD repeat-containing protein 18	Wdr18	2.03 ± 2.17	2.18 ± 2.66	2.22 ± 2.64
Small nuclear ribonucleoprotein Sm D1	Snrpd1	2.03 ± 2.00	2.05 ± 2.04	2.13 ± 1.84
Lysosomal acid phosphatase	Acp2	2.01 ± 1.91	1.45 ± 1.20	1.38 ± 1.26
Ancient ubiquitous protein 1	Aup1	2.00 ± 2.73	2.04 ± 2.78	2.45 ± 3.38

*These values represent average (± standard deviation) fold-change of abundance ratios for each altered protein compared to the high-fat control group (MS-NASH mice on a high-fat diet) with a 2-fold-change threshold in response to Aquamin intervention. For each upregulated protein with Aquamin, corresponding values from the other two groups are shown for comparison. The liver samples (from 5 mice in each group) were individually assessed by TMT-based differential proteomic expression and data were merged to get averages. Protein FDR Confidence for all proteins was ≤ 1% except for three upregulated proteins^#^ ( ≤ 2%). FDR, False Discovery Rate. These data are also presented in Figure 3A*.

In addition to keratins, three members of the tubulin family were also upregulated in response to Aquamin® (Table 2). Tubulins constitute another set of important structural proteins within cells, as they are the major constituents of the microtubule system of the cytoskeleton (48). Of interest, the tubulin proteins were responsive to both low-fat feeding and OCA treatment as well as to Aquamin® (Table 2). Browsing these upregulated (with 2-fold) proteins there are some interesting moieties worth mentioning. Arylsulfatase A (Arsa) was upregulated with Aquamin® with no change in the two other groups. Arsa is regarded as a lysosomal enzyme that is involved in lysophospholipid metabolism and improves insulin sensitivity (49). Another study also suggests its role as a component of the extracellular matrix (50). Another protein, signal transducer and activator of transcription 1 (Stat1) protein was upregulated (at 2.5-fold) with Aquamin® and it is considered an important negative regulator in liver fibrosis by inhibiting stellate cell proliferation through attenuation of TGF-beta signaling (51).

Reactome pathway analysis was utilized to help identify the various pathways influenced by the protein changes noted in Table 2 with Aquamin®. Not surprisingly, pathways related to differentiation were the most highly affected (Table 3). Among these were *keratinization, cornified envelope formation, hemidesmosome assembly, cell junction organization, gap junction formation* and *gap junction assembly*. In addition, pathways involved in intracellular trafficking of proteins as well as protein trafficking between the cytoplasm and plasma membrane were significantly influenced by the protein changes occurring in response to Aquamin®. Protein trafficking events are well-known to depend on cations in the environment (52, 53). Similarly, *plasma lipoproteins clearance* and *LDL clearance* pathways were also upregulated. Another pathway of interest was *hedgehog signaling in the off-state*. Signaling through the hedgehog pathway is associated with chronic liver injury (54, 55) and hedgehog signaling may have implications in hepatocellular carcinomas. Additionally, a pathway known as *regulation of PTEN stability and activity* was also upregulated. Phosphate and tensin homolog (PTEN) gene is considered a tumor suppressor and its loss can contribute to liver injury (56). All of the Aquamin®-responsive Reactome pathways that met the criterial of having an entities *p*-value of <0.05 are presented in Table 3.

**Table 3:** Top pathways associated with upregulated proteins altered (at 2-fold change) with Aquamin.

**Pathway name**	**Entities *p*-value**	**Mapped entities**
Formation of the cornified envelope	1.7 × 10^−7^	Krt2;Krt1;Krt79;Krt16;Krt5;Krt76;Krt17;Krt10;Krt14
Keratinization	3.8 × 10^−5^	Krt2;Krt1;Krt79;Krt16;Krt5;Krt76;Krt17;Krt10;Krt14
Developmental Biology	8.5 × 10^−4^	Krt1;Krt16;Rras;Krt76;Krt14;Tubb2a;Krt2;Krt79;
		Krt5;Tubb4a;Krt17;Krt10;Ap2s1
mRNA Splicing - Minor Pathway	0.002	Sf3b4;Ncbp1;Snrpe;Snrpd1
Hedgehog 'off' state	0.004	Prkar1a;Tubb4a;Psmb8;Psmb3;Tubb2a
SLBP independent Processing of Histone Pre-mRNAs	0.004	Ncbp1;Snrpe
Recycling pathway of L1	0.01	Tubb4a;Tubb2a;Ap2s1
SLBP Dependent Processing of Replication-Dependent Histone Pre-mRNAs	0.01	Ncbp1;Snrpe
Type I hemidesmosome assembly	0.01	Krt5;Krt14
The role of GTSE1 in G2/M progression after G2 checkpoint	0.01	Tubb4a;Psmb8;Psmb3;Tubb2a
Golgi-to-ER retrograde transport	0.01	Pafah1b3;Arf4;Tubb4a;Cope;Tubb2a
Microtubule-dependent trafficking of connexons from Golgi to the plasma membrane	0.01	Tubb4a;Tubb2a
Transport of connexons to the plasma membrane	0.01	Tubb4a;Tubb2a
Signaling by Hedgehog	0.01	Prkar1a;Tubb4a;Psmb8;Psmb3;Tubb2a
COPI-independent Golgi-to-ER retrograde traffic	0.01	Pafah1b3;Tubb4a;Tubb2a
Regulation of RUNX2 expression and activity	0.01	Stub1;Psmb8;Psmb3
COPI-mediated anterograde transport	0.01	Arf4;Tubb4a;Cope;Tubb2a
COPI-dependent Golgi-to-ER retrograde traffic	0.01	Arf4;Tubb4a;Cope;Tubb2a
LDL clearance	0.01	Lipa;Ap2s1
Transcriptional regulation by RUNX2	0.02	Stub1;Psmb8;Psmb3
RHO GTPases Activate NADPH Oxidases	0.02	Pin1;Cybb
MHC class II antigen presentation	0.02	Tubb4a;Cd74;Tubb2a;Ap2s1
Carboxyterminal post-translational modifications of tubulin	0.03	Tubb4a;Tubb2a
Sealing of the nuclear envelope (NE) by ESCRT-III	0.03	Tubb4a;Tubb2a
Regulation of PTEN stability and activity	0.03	Stub1;Psmb8;Psmb3
L1CAM interactions	0.03	Tubb4a;Tubb2a;Ap2s1
Cell junction organization	0.03	Lims1;Krt5;Krt14
G2/M Transition	0.03	Tubb5;Tubb4a;Psmb8;Psmb3;Tubb2a
Processing of Capped Intronless Pre-mRNA	0.03	Ncbp1;Snrpe
Mitotic G2-G2/M phases	0.03	Tubb5;Tubb4a;Psmb8;Psmb3;Tubb2a
Intra-Golgi and retrograde Golgi-to-ER traffic	0.03	Pafah1b3;Arf4;Tubb4a;Cope;Tubb2a
Plasma lipoprotein clearance	0.03	Lipa;Ap2s1
Gap junction assembly	0.04	Tubb4a;Tubb2a
Glycine degradation	0.04	Gcsh
Ion influx/efflux at host-pathogen interface	0.04	Atox1
Metabolism of RNA	0.04	Sf3b4;Ncbp1;Snrpe;Psmb8;Psmb3;Rplp1;
		Patl1;Rpl36;Snrpd1;Wdr18
Aggrephagy	0.04	Tubb4a;Tubb2a
ARMS-mediated activation	0.05	Rap1a
Galactose catabolism	0.05	Galt
PCP/CE pathway	0.05	Psmb8;Psmb3;Ap2s1
ER to Golgi Anterograde Transport	0.05	Arf4;Tubb4a;Cope;Tubb2a
Recruitment of NuMA to mitotic centrosomes	0.05	Tubb5;Tubb4a;Tubb2a
SRP-dependent cotranslational protein targeting to membrane	0.05	Srp19;Rplp1;Rpl36
CREB1 phosphorylation through the activation of Adenylate Cyclase	0.06	Prkar1a
PKA activation in glucagon signaling	0.06	Prkar1a
GDP-fucose biosynthesis	0.06	Fuom
Cell-Cell communication	0.06	Lims1;Krt5;Krt14
Gap junction trafficking	0.06	Tubb4a;Tubb2a
Gap junction trafficking and regulation	0.06	Tubb4a;Tubb2a

*The pathways listed here are altered by the upregulated proteins with the intervention “Aquamin” presented in Table 2. These proteins were selected by using an unbiased approach and 2-fold change threshold. Reactome (v78) was used to generate the pathway analysis report for species Mus musculus. The significance (p-value) is calculated by the overrepresentation analysis (hypergeometric distribution)*.

The database was also searched for proteins that were downregulated by 2-fold or greater with Aquamin®. A total of five individual proteins met this criterion. Of the proteins in the list, four of the five were also statistically significant and presented as part of Table 1C.

### Proteomic Changes in Response to Low-Fat Feeding and to OCA

While the response to Aquamin® was the focus of this study, we also assessed protein changes driven by low-fat feeding or by OCA in high-fat mice. Supplementary Tables 10, 11 show proteins upregulated and downregulated, respectively, by 2-fold or greater with low-fat feeding. Reactome pathways influenced by these protein changes are shown in Supplementary Tables 12, 13. A large majority of the protein changes observed with low-fat feeding (both up- and downregulated) are related to *fat and carbohydrate metabolism*. This is consistent with previous proteomic assessment of diet-influenced protein profile in livers of both rats (57) and mice (58).

Supplementary Tables 14, 15 show protein changes in mice on a high-fat diet treated with OCA. Supplementary Tables 16, 17 show pathways responsive to OCA. The most interesting (if not entirely unexpected) finding was the prominent downregulation of pathways related to *cholesterol metabolism* and *bile acid formation*. Supplementary Table 18 highlights all the pathways involved with upregulated proteins (55 in total at 2-fold-change) common between the two interventions (Aquamin® and OCA). There are some similarities between the two groups mainly due to the proteins of the tubulin family. This finding provides a sharp distinction between the likely mechanistic events driven by Aquamin® and those responsive to OCA. This finding helps validate the utility of the proteomic screening approach employed here.

## Discussion

A wide range of studies (10–12) has demonstrated the susceptibility of rodents to diet-induced NAFLD. Steatosis develops rapidly (within 4–8 weeks) in rats and mice placed on a high-fat or high-fat and -sugar diet. In contrast, end-stage liver disease – i.e., fibrosis/cirrhosis and tumor formation – does not manifest until much later. Tumor formation has been reported to occur as early as after 36 weeks of feeding, but typically 12 or more months' time is required for widespread neoplastic disease to be seen (10–12). In our own previous studies (20, 21), tumors were widespread in male C57BL/6 mice fed a high-fat diet after 12–18 months but almost non-existent in a cohort of mice euthanized at the 5-month time-point. Extensive inflammation, parenchymal necrosis and fibrotic nodules were also seen at the later time-points. Most importantly, our studies demonstrated that providing an adequate supply of dietary minerals along with high-fat feeding substantially reduced the incidence and severity of liver damage. A reduction in tumor formation was especially prominent.

Even though end-stage liver injury in rodents is fully manifested only after an extended feeding period, the cellular and molecular events that bring about damage undoubtedly begin to occur much earlier. It is also likely that interventions that mitigate long-term injury have effects that can be detected much earlier. With this in mind, the present study was carried out to identify hepatic alterations detectable in mice after 16 weeks on a high-fat and -sugar diet compared to control mice on a low-fat diet. As a major part of this study, mineral supplementation along with a different intervention (OCA) with, presumably, different mechanisms of action were also separately evaluated.

In comparison with low-fat-fed control mice, livers were substantially enlarged in high-fat mice. However, there was no gross evidence of more serious liver damage (i.e., fibrotic nodules or tumors) at the 16-week time-point. At the histological level, steatosis was widespread in virtually every mouse on the high-fat diet (not observed in low-fat controls). Ballooning hepatocyte degeneration was also prominent, but inflammation and collagen deposition were minimal. These effects were accompanied by an increase in serum triglycerides and an increase in serum ALT and AST levels. Consistent with previous findings in both rat (57) and mouse (58) models, proteomic assessment demonstrated substantial differences in the protein expression profile between control (low-fat) animals and those on the high-fat diet. The most dramatic differences were in proteins that regulate fat and carbohydrate metabolism. Increases in both lipid storage and lipid oxidation [breakdown] were seen with high-fat feeding, but moieties that regulate protein and nucleic acid turnover were also diet-sensitive. Proteins involved in cholesterol metabolism and bile acid formation were especially sensitive to the high-fat diet. Together, these findings demonstrate that extensive metabolic dysregulation occurs early in response to high-fat-feeding – in parallel with widespread steatosis but while other structural changes in the liver are minimal. The metabolic changes described here should be seen as a “normal” liver's attempt to cope with excessive fat and sugar consumption rather than as evidence of impending liver failure in the MS-NASH mouse model. Even the rise in ALT and AST levels noted here in MS-NASH mice (reaching 300–500 U/mL), while substantial, are not necessarily evidence of serious liver damage. Acute chemical-induced liver toxicity in mice is associated with ALT / AST levels in the range of 3,000–6,000 U/mL (13).

In contrast to the substantial differences distinguishing low-fat and high-fat feeding, mineral supplementation - along with the high-fat diet - had no effect on whole-body weight, gross liver weight or steatosis. Likewise, there was no improvement in serum triglycerides and no decrease in AST and ALT values as compared to what was seen in high-fat control mice. This is consistent with findings reported earlier that mineral supplementation does not prevent steatosis from developing (20, 21) and points to downstream events as targets of Aquamin®.

The protein profile observed in response to intervention with Aquamin® supports this view. While a select few proteins involved directly in fat metabolism / lipid transport were modulated with Aquamin®, the most striking Aquamin®-induced protein changes relate to decreased amino acid metabolism as well as a reduction in purine and pyrimidine catabolism. Also prominent was the increased expression of moieties that are part of the epithelial cell differentiation process. Reactome pathway analysis identified keratinization, cornified envelope formation, hemidesmosome formation, junction formation and gap junction assembly as significantly altered pathways. Proteins involved in microtubule assembly were also upregulated in response to Aquamin® and several pathways related to protein trafficking within the cell or between interior and cell surface were identified.

How the protein changes observed in response to Aquamin® in the current study and the reduction in liver tumor formation seen previously in our earlier long-term studies (20, 21) are related is not fully understood. In the long-term studies with C57BL/6 mice on the HFWD, formation of multiple neoplastic lesions including aberrant foci, both non-regenerative and regenerative hyperplastic nodules, hepatic adenomas and hepatocellular carcinomas were seen. The majority of the tumors occurred in male mice though females were not completely protected. Mice fed a healthy rodent chow diet had many fewer tumors, but even these were not completely protected after 18 months. Hepatic tumor formation in the context of a high-fat diet and steatosis has been extensively studied and multiple contributing mechanisms are thought to be involved (3, 12, 17, 18). Perhaps among them, the most important is lipotoxicity-driven oxidative stress. Oxidant burden, leading to a highly “mutagenic environment” concurrent with high cell turnover resulting from repeated cycles of injury and repair, is a fertile ground for tumor initiation and progression. The formation of toxic and carcinogenic bile acids (a manifestation of lipid over-feeding) may also contribute to tumor formation. The present study identified Aquamin®-sensitive protein changes that suggest reduced catabolism of fats (as well as reduced amino acid, purine and pyrimidine metabolism); all of which could be expected to lower oxidant burden. The reduction in multiple cytochrome P450 enzymes was supportive. The cytochrome P450 enzymes are strongly expressed in liver and rise as part of the organ's response to a toxic environment. Of interest, previous studies have demonstrated that strontium (59), selenium (60), and members of the lanthanide family of rare earth elements (61) – all components of Aquamin® – protect the liver against oxidative stress. While liver tumor formation, *per se*, was not addressed in these earlier studies, reduced oxidative stress and reduced tumor formation are, certainly, consistent.

Aquamin®-induced epithelial cell differentiation provides another potential mechanism to counteract cancer-promotion. A wide range of empirical information supports this view. Epidemiological studies have clearly demonstrated the inverse relationship between differentiation in epithelial cells and tumor incidence in many tissues including those of the gastrointestinal tract (62–66). Animal studies have confirmed this relationship (31, 32, 67) and cell culture studies have provided mechanistic insight into the inverse relationship between differentiation and reduced tumor formation (68, 69). In addition to a reduction in tumor incidence, the expression of differentiation features (assessed histologically) and improved prognosis are correlated with both pre-malignant and malignant epithelial cell tumors (70–74). Calcium, which is the quintessential “driver” of differentiation in epithelial cells (75), is the most abundant mineral in Aquamin®. Thus, epithelial cell differentiation induced by calcium in the multi-mineral product may underlie liver tumor suppression by Aquamin®. It is well-documented, furthermore, that several of the trace elements in the multi-mineral product can act like calcimimetic agonists, promoting a “left-shift” in the calcium dose-response curve. A higher affinity for the extracellular calcium-sensing receptor than calcium itself has been documented with several of the cationic trace elements in Aquamin® (30, 76–78). Similarly, extracellular calcium-sensing receptor has also been expressed in rat hepatocytes (79). Also consistent with this idea is the fact that keratin upregulation was also seen in low-fat mice, though the degree of induction was lower than that observed with Aquamin® itself. It is of interest that while keratin changes were observed in both the low-fat mice and in Aquamin®- supplemented animals (on a high-fat diet), many of the other protein changes observed with Aquamin® supplementation in the high-fat diet were not seen with low-fat feeding. In fact, several were in the “opposite” direction. This strongly suggests, we believe, that mineral supplementation is not just a “surrogate” for a low-fat, healthy diet. Our interpretation of this difference can be summarized as follows: A high-fat diet results in the generation of multiple different moieties (including lipid intermediates) that can be toxic in the liver. The presence of Aquamin could result in smaller amounts of toxic moieties being generated in the first place but, more likely, reflects enhanced detoxification. In contrast, such molecules are simply not generated (to the same extent) in the low-fat diet mice.

In both Aquamin®-treated animals and low-fat-fed mice, the strong keratin upregulation reflected a response in a subset of animals. Why only some animals demonstrated this protein signature is not known, but the finding raises an interesting question: Does the variability in response from animal-to-animal help explain why virtually every mouse fed a high-fat diet rapidly develops steatosis but only a subset progresses to more serious disease? This question cannot be addressed with the presently available data but we have recently initiated a large linear study in which liver biopsies are obtained from mice on a high-fat diet after 16-weeks on diet (as in the present study) following which the animals are maintained for a total of 18-months in order to determine if there is an inverse correlation between keratin expression at the early time-point and tumor formation later.

Although the focus of this study was on mineral supplementation, we also included OCA as a separate intervention for comparison. OCA has been shown to inhibit features of NAFLD / NASH in human patients (22–25) and in mice (26). In the current study, OCA produced a modest reduction in steatosis (histology) and a corresponding reduction in serum triglyceride, AST and ALT levels. In the proteomic screen, there was some overlap in upregulated proteins between Aquamin® and OCA, but OCA did not have the same effect on epithelial differentiation proteins as was seen with Aquamin®. In regard to downregulated proteins, the changes seen with OCA were related to cholesterol metabolism and bile acid synthesis, consistent with OCA's function as a Farnesoid X Receptor agonist (22–25). Given these observations, it is unlikely that the two agents – Aquamin® and OCA – would overlap mechanistically in regard to tumor suppression in a meaningful way. Whether they might produce synergistic anti-tumor activity is not known but is a question worth addressing.

NAFLD is a rising public health challenge. Currently, public health measures focus on “lifestyle” changes, especially diet, to prevent the development of steatosis. Such an approach will not work with everyone. Perhaps efforts directed at reducing the downstream consequences of fatty liver infiltration would be of value. It should be noted in this regard that a majority of individuals in Western society do not meet USDA daily intake guidelines for calcium (80, 81). Many individuals are also deficient in magnesium (82) and, presumably, in other minerals that are nutritionally associated with calcium and magnesium. Inadequate mineral intake is not limited to individuals consuming a Western-style diet. A recent study demonstrated that a majority of individuals in many developing regions of the world also lacked an adequate amount of calcium in their diet (83, 84). Whether the mineral supplement used here or some other formulation might provide a way to ensure adequate mineral intake remains to be seen. We have recently completed a 90-day pilot phase trial in which 30 healthy human subjects were randomized to receive Aquamin® formulated to provide 800 mg of calcium per day, calcium carbonate at the same level or placebo (38, 85). To summarize, no safety or tolerability issues were seen with Aquamin®. At the same time, colon biopsies obtained before and after treatment demonstrated upregulation of several differentiation-related proteins in the colonic mucosa. In the calcium-alone group, differentiation proteins were also induced, but the levels of increase were lower than seen with Aquamin®. Finally, a decrease in the levels of certain primary and secondary bile acids was also observed in subjects receiving Aquamin® in conjunction with altered gut microbial profile. These metabolomic and microbial changes were not observed with calcium alone. While the focus of these clinical studies has been colonic health, the same approach may provide benefit in the liver.

**Figure 6:**
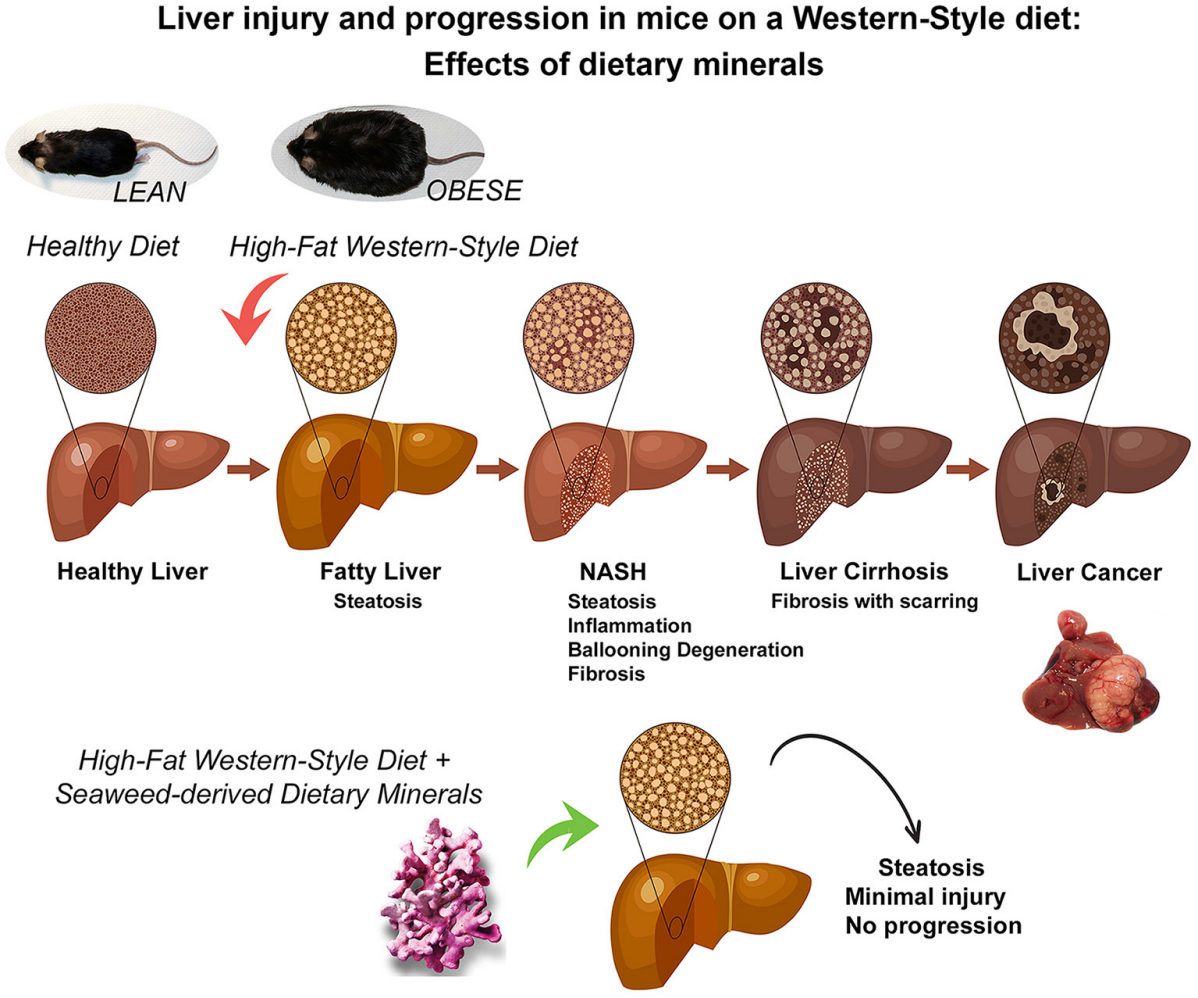
A graphical summary illustrating the mitigating effects of dietary minerals on the high-fat induced liver injury and consequences of liver injury. Some people with non-alcoholic fatty liver disease develop a more aggressive form known as non-alcoholic steatohepatitis (NASH) in which the liver becomes inflamed, develops ballooning degeneration and progresses to fibrosis. In the long-term, this can lead to advanced scarring known as cirrhosis, which can cause liver failure and cancer. However, Aquamin, which is rich in calcium, magnesium and additional minerals and trace elements (derived from calcified red algae), could prevent the adverse downstream effects activated by the high-fat diet (as demonstrated in previous long-term murine studies of up to 18-month and current 16-week mechanistic study).

In summary, our past studies have clearly demonstrated the importance of adequate mineral intake for preventing the downstream consequences of fatty liver accumulation in a murine model (as summarized in the graphic cartoon – Figure 6). The present studies provide mechanistic insight into how mineral supplementation may contribute to the reduction in liver tumor formation, one of the most devastating consequences of fatty liver disease in the face of steatosis.

## Data Availability Statement

All relevant data are within the manuscript and its Supporting Information files. The mass spectrometry proteomics data are available on ProteomeXchange Consortium (PRIDE partner repository) – dataset identifier PXD030954.

## Ethics Statement

The animal study was reviewed and approved by the Institutional Animal Care and Use Committee (IACUC). Crown Bio, Inc., is an American Association for Accreditation of Laboratory Animal Care (AAALAC)-Accredited Institution.

## Author Contributions

All listed authors have contributed toward this project to attain authorship status according to ICMJE criteria. Their specific contributions are as follows: MA and JV: conceptualization, methodology, resources, writing—original draft preparation, supervision, and project administration. MA: software and visualization. MA, SM, RK, and JV: validation. MA, SM, RK, IH, DZ, and MJ-M: formal analysis and data curation. MA, SM, RK, IH, DZ, MJ-M, MA, and JV: investigation and writing—review and editing. MA and JV: funding acquisition. All authors have read and agreed to this version of the manuscript.

## Funding

This study was funded by NIH grant CA201782 including supplemental funding through the Office of Dietary Supplements to JV and funding from the American Society for Investigative Pathology (ASIP) Summer Research Opportunity Program in Pathology (SROPP) to MA.

## Conflict of Interest

The authors declare that the research was conducted in the absence of any commercial or financial relationships that could be construed as a potential conflict of interest.

## Publisher's Note

All claims expressed in this article are solely those of the authors and do not necessarily represent those of their affiliated organizations, or those of the publisher, the editors and the reviewers. Any product that may be evaluated in this article, or claim that may be made by its manufacturer, is not guaranteed or endorsed by the publisher.

## Supplementary Material

Supplementary Table 1 Mineral Composition of Aquamin® Soluble.

Supplementary Table 2 Common and unique upregulated proteins (at 2-fold change threshold).

Supplementary Table 3 Common and unique downregulated proteins (at 2-fold change threshold).

Supplementary Table 4 Comprehensive list of upregulated (and significantly altered) proteins with Aquamin® in high-fat mice.

Supplementary Table 5 Top pathways associated with significantly upregulated proteins (shown in Supplementary Table 4) altered with Aquamin®.

Supplementary Table 6 Comprehensive list of downregulated (and significantly altered) proteins with Aquamin® in high-fat mice.

Supplementary Table 7 Top pathways associated with significantly downregulated proteins (shown in Supplementary Table 6) altered with Aquamin®.

Supplementary Table 8 String enrichment analysis of upregulated proteins (presented in Supplementary Table 4).

Supplementary Table 9 String enrichment analysis of downregulated proteins (presented in Supplementary Table 6).

Supplementary Table 10 Upregulated Proteins by an unbiased proteomic screening of C57BL6 mice on low-fat diet.

Supplementary Table 11 Downregulated Proteins by an unbiased proteomic screening of C57BL6 mice on low-fat diet.

Supplementary Table 12 Top pathways associated with upregulated proteins altered with low-fat diet in C57BL6 mice.

Supplementary Table 13 Top pathways associated with downregulated proteins altered with low-fat diet in C57BL6 mice.

Supplementary Table 14 Upregulated Proteins by an unbiased proteomic screening with Obeticholic acid (OCA) in high-fat mice.

Supplementary Table 15 Downregulated Proteins by an unbiased proteomic screening with Obeticholic acid (OCA) in high-fat mice.

Supplementary Table 16 Top pathways associated with upregulated proteins altered with Obeticholic acid (OCA).

Supplementary Table 17 Top pathways associated with downregulated proteins altered with Obeticholic acid (OCA).

Supplementary Table 18 Top pathways associated with upregulated (and common) proteins altered with Aquamin® and Obeticholic acid (OCA).

## References

[1] Younossi Z Anstee QM Marietti M Hardy T Henry L Eslam M. Global burden of NAFLD and NASH: trends, predictions, risk factors and prevention. Nature Rev Gastroenterol Hepatol. (2018) 15:11–20. 10.1038/nrgastro.2017.109 28930295

[2] Younossi ZM Koenig AB Abdelatif D Fazel Y Henry L Wymer M. Global epidemiology of non-alcoholic fatty liver disease-Meta-analytic assessment of prevalence, incidence, and outcomes. Hepatology. (2016) 64:73–84. 10.1002/hep.28431 26707365

[3] Anstee QM Reeves HL Kotsiliti E Govaere O Heikenwalder M. From NASH to HCC: current concepts and future challenges. Nat Rev Gastroenterol Hepatol. (2019) 16:411–28. 10.1038/s41575-019-0145-7 31028350

[4] Cotter TG Rinella M. Non-alcoholic fatty liver disease 2020: the state of the disease. Gastroenterology. (2020) 158:1851–64. 10.1053/j.gastro.2020.01.052 32061595

[5] Sheka AC Adeyi O Thompson J Hameed B Crawford PA Ikramuddin S. Non-alcoholic steatohepatitis: a review. JAMA. (2020) 323:1175–83. 10.1001/jama.2020.2298 32207804

[6] Hardy T Oakley F Anstee QM Day CP. Non-alcoholic fatty liver disease: pathogenesis and disease spectrum. Annu Rev Pathol. (2016) 11:451–96. 10.1146/annurev-pathol-012615-044224 26980160

[7] Day CP James OF. Steatohepatitis: a tale of two “hits”? Gastroenterology. (1998) 114:842–5. 10.1016/s0016-5085(98)70599-2 9547102

[8] Day CP Saksena S. Non-alcoholic steatohepatitis: definitions and pathogenesis. J Gastroenterol Hepatol. (2002) 17(Suppl 3):S377–84. 10.1046/j.1440-1746.17.s3.31.x 12472967

[9] Fengler VH Macheiner T Kessler SM Czepukojc B Gemperlein K Müller R. Susceptibility of different mouse wild type strains to develop diet-induced NAFLD/AFLD-associated liver disease. PLoS ONE. (2016) 11:e0155163. 10.1371/journal.pone.0155163 27167736 PMC4863973

[10] Nevzorova YA Boyer-Diaz Z Cubero FJ Gracia-Sancho J. Animal models for liver disease - A practical approach for translational research. J Hepatol. (2020) 73:423–40. 10.1016/j.jhep.2020.04.011 32330604

[11] Clapper JR Hendricks MD Gu G Wittmer C Dolman CS Herich J. Diet-induced mouse model of fatty liver disease and non-alcoholic steatohepatitis reflecting clinical disease progression and methods of assessment. Am J Physiol Gastrointestinal Liver Physiol. (2013) 305:G483–95. 10.1152/ajpgi.00079.2013 23886860

[12] Febbraio MA Reibe S Shalapour S Ooi GJ Watt MJ Karin M. Preclinical models for studying NASH-driven HCC: how useful are they? Cell Metab. (2019) 29:18–26. 10.1016/j.cmet.2018.10.012 30449681 PMC6326872

[13] Zhang G Wang X Chung TY Ye W Hodge L Zhang L. Carbon tetrachloride (CCl4) accelerated development of non-alcoholic fatty liver disease (NAFLD)/steatohepatitis (NASH) in MS-NASH mice fed western diet supplemented with fructose (WDF). BMC Gastroenterol. (2020) 20:339. 10.1186/s12876-020-01467-w 33059584 PMC7560288

[14] Brol MJ Rösch F Schierwagen R Magdaleno F Uschner FE Manekeller S. Combination of CCl_4_ with alcoholic and metabolic injuries mimics human liver fibrosis. Am J Physiol Gastrointestinal Liver Physiol. (2019) 317:G182–94. 10.1152/ajpgi.00361.2018 31188634

[15] Honda H Ikejima K Hirose M Yoshikawa M Lang T Enomoto N. Leptin is required for fibrogenic responses induced by thioacetamide in the murine liver. Hepatology. (2002) 36:12–21. 10.1053/jhep.2002.33684 12085344

[16] Marcolin E Forgiarini LF Tieppo J Dias AS Freitas LA Marroni NP. Methionine- and choline-deficient diet induces hepatic changes characteristic of non-alcoholic steatohepatitis. Arquivos de Gastroenterologia. (2011) 48:72–9. 10.1590/s0004-28032011000100015 21537547

[17] Zaki M Mahdi AK Patman GL Whitehead A Maurício JP McCain MV. Key features of the environment promoting liver cancer in the absence of cirrhosis. Sci Rep. (2021) 11:16727. 10.1038/s41598-021-96076-2 34408183 PMC8373870

[18] Dowman JK Hopkins LJ Reynolds GM Nikolaou N Armstrong MJ Shaw JC. Development of hepatocellular carcinoma in a murine model of non-alcoholic steatohepatitis induced by use of a high-fat/fructose diet and sedentary lifestyle. Am J Pathol. (2014) 184:1550–61. 10.1016/j.ajpath.2014.01.034 24650559 PMC4005975

[19] Tetri LH Basaranoglu M Brunt EM Yerian LM Neuschwander-Tetri BA. Severe NAFLD with hepatic necroinflammatory changes in mice fed trans fats and a high-fructose corn syrup equivalent. Am J Physiol Gastrointestinal Liver Physiol. (2008) 295:G987–95. 10.1152/ajpgi.90272.2008 18772365 PMC4059366

[20] Nadeem Aslam M Bassis CM Zhang L Zaidi S Varani J Bergin IL. Calcium reduces liver injury in mice on a high-fat diet: alterations in microbial and bile acid profiles. PLoS ONE. (2016) 11:e0166178. 10.1371/journal.pone.0166178 27851786 PMC5113033

[21] Aslam MN Bergin I Naik M Hampton A Allen R Kunkel SL. A multi-mineral natural product inhibits liver tumor formation in C57BL/6 mice. Biol Trace Elem Res. (2012) 147:267–74. 10.1007/s12011-011-9316-2 22222483 PMC3360994

[22] Sepe V Distrutti E Fiorucci S Zampella A. Farnesoid X receptor modulators 2014-present: a patent review. Expert Opin Ther Pat. (2018) 28:351–64. 10.1080/13543776.2018.1459569 29649907

[23] Neuschwander-Tetri BA. Farnesoid x receptor agonists: what they are and how they might be used in treating liver disease. Current Gastroenterol Reports. (2012) 14:55–62. 10.1007/s11894-011-0232-6 22094889

[24] Mudaliar S Henry RR Sanyal AJ Morrow L Marschall HU Kipnes M. et al. Efficacy and safety of the farnesoid X receptor agonist obeticholic acid in patients with type 2 diabetes and non-alcoholic fatty liver disease. Gastroenterology. (2013) 145:574–82. 10.1053/j.gastro.2013.05.042 23727264

[25] Neuschwander-Tetri BA Loomba R Sanyal AJ Lavine JE Van Natta ML Abdelmalek MF. Farnesoid X nuclear receptor ligand obeticholic acid for non-cirrhotic, non-alcoholic steatohepatitis (FLINT): a multicentre, randomised, placebo-controlled trial. Lancet. (2015) 385:956–65. 10.1016/S0140-6736(14)61933-4 25468160 PMC4447192

[26] Haczeyni F Poekes L Wang H Mridha AR Barn V Geoffrey Haigh W. Obeticholic acid improves adipose morphometry and inflammation and reduces steatosis in dietary but not metabolic obesity in mice. Obesity. (2017) 25:155–65. 10.1002/oby.21701 27804232 PMC5849463

[27] Adey W McKibbin D. Studies on the maerl species Phymatolithon calcareum (Pallas) nov. Comb and Lithothamnium coralloides Crouan in the Ria de Vigo. (1970) 13:100–6. 10.1515/botm.1970.13.2.100

[28] Ward DT Riccardi D. New concepts in calcium-sensing receptor pharmacology and signalling. Br J Pharmacol. (2012) 165:35–48. 10.1111/j.1476-5381.2011.01511.x 21627634 PMC3252964

[29] Pi M Faber P Ekema G Jackson PD Ting A Wang N. Identification of a novel extracellular cation-sensing G-protein-coupled receptor. J Biol Chem. (2005) 280:40201–9. 10.1074/jbc.M505186200 16199532 PMC1435382

[30] Attili D Jenkins B Aslam MN Dame MK Varani J. Growth control in colon epithelial cells: gadolinium enhances calcium-mediated growth regulation. Biol Trace Elem Res. (2012) 150:467–76. 10.1007/s12011-012-9503-9 23008064 PMC3661021

[31] Aslam MN Paruchuri T Bhagavathula N Varani J. A mineral-rich red algae extract inhibits polyp formation and inflammation in the gastrointestinal tract of mice on a high-fat diet. Integrative Cancer Therap. (2010) 9:93–9. 10.1177/1534735409360360 20150219 PMC2861409

[32] Aslam MN Bergin I Naik M Paruchuri T Hampton A Rehman M. A multimineral natural product from red marine algae reduces colon polyp formation in C57BL/6 mice. Nutr Cancer. (2012). 64:1020–8. 10.1080/01635581.2012.713160 23035966 PMC3660990

[33] Sun G Jackson CV Zimmerman K Zhang LK Finnearty CM Sandusky GE. The FATZO mouse, a next generation model of type 2 diabetes, develops NAFLD and NASH when fed a Western diet supplemented with fructose. BMC Gastroenterol. (2019) 19:41. 10.1186/s12876-019-0958-4 30885145 PMC6421686

[34] Neff EP. Farewell, FATZO: a NASH mouse update. Lab Animal. (2019). 48:151. 10.1038/s41684-019-0311-0 31061508

[35] Kleiner DE Brunt EM Van Natta M Behling C Contos MJ Cummings OW. Design and validation of a histological scoring system for non-alcoholic fatty liver disease. Hepatology. (2005) 41:1313–21. 10.1002/hep.20701 15915461

[36] Pais P D'Amato M. *In vivo* efficacy study of milk thistle extract (ETHIS-094™) in STAM™ model of non-alcoholic steatohepatitis. Drugs in R&D. (2014) 14:291–9. 10.1007/s40268-014-0068-2 25404123 PMC4269824

[37] Piguet AC Saran U Simillion C Keller I Terracciano L Reeve. Regular exercise decreases liver tumors development in hepatocyte-specific PTEN-deficient mice independently of steatosis. J Hepatol. (2015) 62:1296–303. 10.1016/j.jhep.2015.01.017 25623824

[38] Aslam MN McClintock SD Jawad-Makki M Knuver K Ahmad HM Basrur V. A multi-mineral intervention to modulate colonic mucosal protein profile: results from a 90-day trial in human subjects. Nutrients. (2021) 13:939. 10.3390/nu13030939 33799486 PMC8002192

[39] Attili D. McClintock S. D. Rizvi A. H. Pandya S. Rehman H. Nadeem D. M.. Calcium-induced differentiation in normal human colonoid cultures: cell-cell / cell-matrix adhesion, barrier formation and tissue integrity. PLoS ONE. (2019) 14:e0215122. 10.1371/journal.pone.0215122 30995271 PMC6469792

[40] Aslam MN McClintock SD Attili D Pandya S Rehman H Nadeem DM. Ulcerative colitis-derived colonoid culture: a multi-mineral-approach to improve barrier protein expression. Front Cell Develop Biol. (2020) 8:577221. 10.3389/fcell.2020.577221 33330453 PMC7719760

[41] McAlister GC Nusinow DP Jedrychowski MP Wühr M Huttlin EL Erickson BK. MultiNotch MS3 enables accurate, sensitive, and multiplexed detection of differential expression across cancer cell line proteomes. Anal Chem. (2014) 86:7150–8. 10.1021/ac502040v 24927332 PMC4215866

[42] Fabregat A Sidiropoulos K Garapati P Gillespie M Hausmann K Haw R. The reactome pathway knowledgebase. Nuc Acids Res. (2016) 44:D481–7. 10.1093/nar/gkv1351 26656494 PMC4702931

[43] Georgila K Vyrla D Drakos E. Apolipoprotein A-I (ApoA-I), Immunity, inflammation, and cancer. Cancers. (2019) 11:1097. 10.3390/cancers11081097 31374929 PMC6721368

[44] McDonnell AM Dang CH. Basic review of the cytochrome p450 system. J Adv Pract Oncol. (2013), 4:263–8. 10.6004/jadpro.2013.4.4.7 25032007 PMC4093435

[45] Robertson G Leclercq I Farrell GC. Non-alcoholic steatosis and steatohepatitis. II Cytochrome P-450 enzymes and oxidative stress. Am J Physiol Gastro Liver physiol. (2001) 281:G1135–9. 10.1152/ajpgi.2001.281.5.G1135 11668021

[46] Golob-Schwarzl N Bettermann K Mehta AK Kessler SM Unterluggauer J Krassnig S. High Keratin 8/18 ratio predicts aggressive hepatocellular cancer phenotype. Transl Oncol. (2019) 12:256–68. 10.1016/j.tranon.2018.10.010 30439626 PMC6234703

[47] Dmello C Srivastava SS Tiwari R Chaudhari PR Sawant S Vaidya MM. Multifaceted role of keratins in epithelial cell differentiation and transformation. J Biosci. (2019) 44:33. 10.1007/s12038-019-9864-8 31180046

[48] Binarová P Tuszynski J. Tubulin: structure, functions, and roles in disease. Cells. (2019) 8:1294. 10.3390/cells8101294 31652491 PMC6829893

[49] Montgomery MK Bayliss J Nie S De Nardo W Keenan SN Miotto PM. Deep proteomic profiling unveils arylsulfatase A as a non-alcoholic steatohepatitis inducible hepatokine and regulator of glycemic control. Nat Commun. (2022) 13:1259. 10.1038/s41467-022-28889-2 35273160 PMC8913628

[50] Fujita K Teramura N Hattori S Irie S Mitsunaga-Nakatsubo K Akimoto Y. Mammalian arylsulfatase A functions as a novel component of the extracellular matrix. Connect Tissue Res. (2010) 51:388–96. 10.3109/03008200903537097 20297902

[51] Jeong WI Park O Radaeva S Gao B. STAT1 inhibits liver fibrosis in mice by inhibiting stellate cell proliferation and stimulating NK cell cytotoxicity. Hepatology. (2006) 44:1441–51. 10.1002/hep.21419 17133483

[52] Graber ZT Shi Z Baumgart T. Cations induce shape remodeling of negatively charged phospholipid membranes. Phys Chem Chem Phy: PCCP. (2017) 19:15285–95. 10.1039/c7cp00718c 28569910 PMC5562360

[53] Tiwari S Askari JA Humphries MJ Bulleid NJ. Divalent cations regulate the folding and activation status of integrins during their intracellular trafficking. J Cell Sci. (2011) 124:1672–80. 10.1242/jcs.084483 21511727 PMC3085436

[54] Shen X Peng Y Li H. The injury-related activation of hedgehog signaling pathway modulates the repair-associated inflammation in liver fibrosis. Front Immunol. (2017) 8:1450. 10.3389/fimmu.2017.01450 29163520 PMC5681491

[55] Huang S He J Zhang X Bian Y Yang L Xie G. Activation of the hedgehog pathway in human hepatocellular carcinomas. Carcinogenesis. (2006) 27:1334–40. 10.1093/carcin/bgi378 16501253

[56] Chen CY Chen J He L Stiles BL. PTEN: tumor suppressor and metabolic regulator. Front Endocrinol. (2018) 9:338. 10.3389/fendo.2018.00338 30038596 PMC6046409

[57] Sang J Qu H Gu R Chen D Chen X Yin B. Proteomics study of the effect of high-fat diet on rat liver. Br J Nutr. (2019) 122:1062–72. 10.1017/S0007114519001740 31311615

[58] Khan ZN Leite A Charone S Sabino I Martini T Pereira HA. Liver proteome of mice with different genetic susceptibilities to the effects of fluoride. J App Oral Sci: Revista FOB. (2016) 24:250–7. 10.1590/1678-775720150364 27383706 PMC5022220

[59] Jiang H Guan Q Xiao Y Feng Z Yu G Pan Q. Strontium alleviates endoplasmic reticulum stress in a non-alcoholic fatty liver disease model. J Med Food. (2018) 21:1228–37. 10.1089/jmf.2018.4186 30457429

[60] Miyata M Matsushita K Shindo R Shimokawa Y Sugiura Y Yamashita M. Selenoneine ameliorates hepatocellular injury and hepatic steatosis in a mouse model of NAFLD. Nutrients. (2020) 12:1898. 10.3390/nu12061898 32604760 PMC7353312

[61] Li R Yu L Qin Y Zhou Y Liu W Li Y. Protective effects of rare earth lanthanum on acute ethanol-induced oxidative stress in mice via Keap 1/Nrf2/p62 activation. Sci Total Environ. (2021) 758:143626. 10.1016/j.scitotenv.2020.143626 33243512

[62] Keum N Aune D Greenwood DC Ju W Giovannucci EL. Calcium intake and colorectal cancer risk: dose-response meta-analysis of prospective observational studies. Int J Cancer. (2014) 135:1940–8. 10.1002/ijc.28840 24623471

[63] Kesse E Boutron-Ruault MC Norat T Riboli E Clavel-Chapelon F E3N Group. Dietary calcium, phosphorus, vitamin D, dairy products and the risk of colorectal adenoma and cancer among French women of the E3N-EPIC prospective study. Int J Cancer. (2005) 117:137–44. 10.1002/ijc.21148 15880532

[64] Flood A Peters U Chatterjee N Lacey JV Jr Schairer C Schatzkin A. Calcium from diet and Supplements is associated with reduced risk of colorectal cancer in a prospective cohort of women. Cancer Epidemiol Biomarkers Prev. (2005) 14:126–32. 10.1158/1055-9965.126.14.1 15668485

[65] Shaukat A Scouras N Schünemann HJ. Role of supplemental calcium in the recurrence of colorectal adenomas: a metaanalysis of randomized controlled trials. Am J Gastroenterol. (2005) 100:390–4. 10.1111/j.1572-0241.2005.41220.x 15667497

[66] Larsson SC Bergkvist L Rutegård J Giovannucci E Wolk A. Calcium and dairy food intakes are inversely associated with colorectal cancer risk in the Cohort of Swedish men. Am J Clin Nutr. (2006) 83:667–729. 10.1093/ajcn.83.3.667 16522915

[67] Newmark HL Yang K Kurihara N Fan K Augenlicht LH Lipkin M. Western-style diet-induced colonic tumors and their modulation by calcium and vitamin D in C57Bl/6 mice: a preclinical model for human sporadic colon cancer. Carcinogenesis. (2009) 30:88–92. 10.1093/carcin/bgn229 19017685 PMC2722141

[68] Mariadason JM Bordonaro M Aslam F Shi L Kuraguchi M Velcich A. Down-regulation of beta-catenin TCF signaling is linked to colonic epithelial cell differentiation. Cancer Res. (2001) 61:3465–71. 11309309

[69] Chakrabarty S Radjendirane V Appelman H Varani J. Extracellular calcium and calcium sensing receptor function in human colon carcinomas: promotion of E-cadherin expression and suppression of beta-catenin/TCF activation. Cancer Res. (2003) 63:67–71. 12517779

[70] Sievers CK Leystra AA Clipson L Dove WF Halberg RB. Understanding intratumoral heterogeneity: lessons from the analysis of at-risk tissue and premalignant lesions in the colon. Cancer Prevent Res). (2016) 9:638–41. 10.1158/1940-6207.CAPR-16-0096 27199343 PMC4970935

[71] Morson BC Whiteway JE Jones EA Macrae FA Williams CB. Histopathology and prognosis of malignant colorectal polyps treated by endoscopic polypectomy. Gut. (1984) 25:437–44. 10.1136/gut.25.5.437 6714785 PMC1432448

[72] Cunningham KN Mills LR Schuman BM Mwakyusa DH. Long-term prognosis of well-differentiated adenocarcinoma in endoscopically removed colorectal adenomas. Dig Dis Sci. (1994) 39:2034–7. 10.1007/BF02088143 8082514

[73] Xu B Yu L Zhao LZ Ma DW. Prognostic factors in the patients with T2N0M0 colorectal cancer. World J Surg Oncol. (2016) 14:76. 10.1186/s12957-016-0826-4 26965721 PMC4785652

[74] Xiao H Yoon YS Hong SM Roh SA Cho DH Yu CS. Poorly differentiated colorectal cancers: correlation of microsatellite instability with clinicopathologic features and survival. Am J Clin Pathol. (2013) 140:341–7. 10.1309/AJCP8P2DYNKGRBVI 23955452

[75] Stewart TA Yapa KT Monteith GR. (2015). Altered calcium signaling in cancer cells. Biochimica et biophysica acta. (1848):2502–11. 10.1016/j.bbamem.2014.08.016 25150047

[76] Takaoka S Yamaguchi T Yano S Yamauchi M Sugimoto T. The Calcium-sensing Receptor (CaR) is involved in strontium ranelate-induced osteoblast differentiation and mineralization. Hormone Metabol Res = *Hormon- und Stoffwechselforschung* = *Hormones et metabolism*. (2010) 42:627–31. 10.1055/s-0030-1255091 20560105

[77] Huang Y Zhou Y Castiblanco A Yang W Brown EM Yang JJ. Multiple Ca(2+)-binding sites in the extracellular domain of the Ca(2+)-sensing receptor corresponding to cooperative Ca(2+) response. Biochemistry. (2009) 48:388–98. 10.1021/bi8014604 19102677 PMC2627791

[78] Carrillo-López N Fernández-Martín JL Alvarez-Hernández D González-Suárez I Castro-Santos P Román-García P. Lanthanum activates calcium-sensing receptor and enhances sensitivity to calcium. Nephrology, dialysis, transplantation: official publication of the European dialysis and transplant association. Eur Renal Assoc. (2010) 25:2930–7. 10.1093/ndt/gfq124 20233740

[79] Canaff L Petit JL Kisiel M Watson PH Gascon-Barré M Hendy GN. Extracellular calcium-sensing receptor is expressed in rat hepatocytes. Coupling to intracellular calcium mobilization and stimulation of bile flow. J Biol Chem. (2001) 276:4070–9. 10.1074/jbc.M009317200 11071898

[80] U.S. Department of Health and Human Services; U.S. Department of Agriculture. 2015-−2020 Dietary Guidelines for Americans. December 2015, 8th ed.; U.S. Department of Health and Human Services: Washington, DC, USA; U.S. Department of Agriculture: Washington, DC, USA, 2015. Available online at: https://health.gov/our-work/food-and-nutrition/2015–2020-dietary-guidelines/. (accessed January 6, 2022).

[81] Aslam MN Varani J. The western-style diet, calcium deficiency and chronic disease. J Nutr Food Sci. (2016) 6:3. 10.4172/2155-9600.1000496

[82] Lennie TA Andreae C Rayens MK Song EK Dunbar SB Pressler SJ. Micronutrient deficiency independently predicts time to event in patients with heart failure. J Am Heart Assoc. (2018) 7:e007251. 10.1161/JAHA.117.007251 30371170 PMC6201427

[83] Bai Y Herforth A Masters WA. (2022). Global variation in the cost of a nutrient-adequate diet by population group: an observational study. Lancet Planetary Health. 6:e19–28. 10.1016/S2542-5196(21)00285-0 34998455 PMC8753783

[84] Kopru S Cadir M Soylak M. Investigation of trace elements in vegan foods by ICP-MS after microwave digestion. Biol Trace Element Res. (2022). 10.1007/s12011-022-03106-9 35006553

[85] Aslam MN Bassis CM Bergin IL Knuver K Zick SM Sen A. A calcium-rich multi-mineral intervention to modulate colonic microbial communities and metabolomic profiles in humans: results from a 90-day trial. Cancer Prevention Res. (2020) 13:101–16. 10.1158/1940-6207.CAPR-19-0325 31771942 PMC7528938

