# Supplementary material for: Liver Protein Expression in NASH Mice on a High-Fat Diet: Response to Multi-Mineral Intervention
Source: Front Nutr. 2022 May 11;9:859292. doi: 10.3389/fnut.2022.859292 (PMC9130755; doi:10.3389/fnut.2022.859292)
Supplement: Supplementary Table 1 — Mineral Composition of Aquamin® Soluble. [file Data_Sheet_1.zip › SM Table 10 859292.pdf]

Supplement Table 10. Upregulated Proteins by an unbiased proteomic screening of C57BL6 mice on low-fat diet

		C57BL6	MS-NASH	
**Proteins**	**Genes**	**Control**	**OCA**	**Aquamin**
Alpha-1-antitrypsin 1-5	Serpina1e	37.05±11.87*	0.70±0.35	0.52±0.27
NADPH-dependent 3-keto-steroid reductase Hsd3b5	Hsd3b5	19.97±21.84	0.78±0.26	0.83±0.12
Solute carrier organic anion transporter family member 1A1	Slco1a1	18.33±10.59*	1.03±0.73	1.05±0.66
Cytochrome P450 7B1	Cyp7b1	15.31±8.01*	0.63±0.31	0.74±0.21
Glycine cleavage system H protein, mitochondrial	Gcsh	11.09±21.41	9.05±17.88	7.63±14.93
Major urinary protein 20	Mup20	10.78±14.91	1.02±0.37	0.90±0.50
Major urinary protein 2	Mup2	10.40±5.30*	0.44±0.19	0.59±0.23
Fucose mutarotase	Fuom	8.72±17.40	10.03±19.94	10.28±20.61
UDP-glucuronosyltransferase 3A2	Ugt3a2	8.39±3.65*	0.85±0.07	0.96±0.13
Nuclear transport factor 2	Nutf2	8.16±16.12	10.29±20.76	12.12±24.59
Ras-related protein R-Ras	Rras	6.32±11.74	4.75±8.44	5.46±9.57
Glutathione S-transferase P 1	Gstp1	6.24±2.43*	0.92±0.14	1.14±0.15
Epidermal growth factor receptor	Egfr	6.21±1.51*	0.79±0.19*	0.87±0.18
Alpha-1-acid glycoprotein 1	Orm1	6.12±9.25	2.93±5.05	3.98±6.98
SH3 domain-containing protein 21[#]	Sh3d21	6.05±10.62	5.43±10.12	8.30±16.34
Transmembrane protein 14C	Tmem14c	5.97±10.43	4.82±8.80	5.56±10.29
Keratin, type II cytoskeletal 2 oral	Krt76	5.89±7.01	1.71±2.42	8.07±14.08
Keratin, type I cytoskeletal 14	Krt14	5.88±7.13	0.70±0.43	5.58±9.45
1,5-anhydro-D-fructose reductase	Akr1e2	5.75±1.76*	1.65±1.00	1.24±0.50
Ras-related protein Rap-1A	Rap1a	5.66±10.21	5.83±10.73	5.20±9.18
Selenium-binding protein 2	Selenbp2	5.63±0.99*	1.24±0.31	1.07±0.23
Carboxylesterase 3B	Ces3b	5.56±2.65*	1.34±0.32*	0.71±0.16
Isopentenyl-diphosphate Delta-isomerase 1	Idi1	5.50±4.41	1.62±0.95	0.98±0.35
Keratin, type II cytoskeletal 79	Krt79	5.40±4.07	5.73±7.97	26.34±43.11
Tubulin beta-5 chain	Tubb5	4.90±9.25	5.06±9.36	5.81±10.51
60S acidic ribosomal protein P1	Rplp1	4.84±8.27	3.61±5.97	3.53±5.59
Cytochrome P450 2C50	Cyp2c50	4.82±2.53*	0.79±0.40	0.65±0.22
Methylsterol monooxygenase 1	Msmo1	4.54±1.38*	1.38±0.45	1.15±0.54
Isoamyl acetate-hydrolyzing esterase 1 homolog	Iah1	4.50±1.12*	1.00±0.24	0.98±0.27
ATP-binding cassette sub-family D member 4	Abcd4	4.49±4.10	2.12±1.99	1.50±1.19
Adenylate kinase 4, mitochondrial	Ak4	4.42±1.10*	1.75±0.21*	1.00±0.13
60S ribosomal protein L36	Rpl36	4.35±8.31	4.95±8.52	4.32±7.31
Inositol-3-phosphate synthase 1	Isyna1	4.32±5.70	0.79±0.23	0.90±0.24

Lanosterol 14-alpha demethylase	Cyp51a1	4.21±2.01*	1.38±0.72	0.99±0.30
IgG receptor FcRn large subunit p51	Fcgrt	4.17±6.07	5.74±10.39	4.12±6.83
Keratin, type I cytoskeletal 10	Krt10	4.10±6.43	1.99±3.15	4.86±8.83
Ig alpha chain C region	n/a	4.06±1.96*	0.96±0.40	1.03±0.48
Pyrethroid hydrolase Ces2a	Ces2a	4.00±1.62*	1.49±0.51	0.96±0.27
Keratin, type II cytoskeletal 1	Krt1	3.96±3.36	1.80±2.51	10.38±20.44
4-hydroxy-2-oxoglutarate aldolase, mitochondrial	Hoga1	3.81±2.81	2.05±1.87	1.27±0.85
UDP-glucuronosyltransferase 1-9	Ugt1a9	3.78±4.76	1.73±1.63	1.68±1.68
Glutaredoxin-1	Glrx	3.76±5.34	2.85±3.94	2.58±3.23
Tubulin beta-4A chain	Tubb4a	3.76±6.90	4.73±8.30	4.85±8.51
Keratin, type I cytoskeletal 42	Krt42	3.74±3.98	1.26±1.63	7.91±13.57
Aldehyde oxidase 2	Aox2	3.70±2.76	1.86±0.89	1.22±0.36
Serum amyloid A-1 protein	Saa1	3.65±4.61	0.45±0.19	0.73±0.40
Carbamoyl-phosphate synthase [ammonia], mitochondrial	Cps1	3.62±2.04*	1.39±0.35*	0.90±0.33
Bile salt sulfotransferase 1	Sult2a1	3.60±2.35*	0.98±0.50	0.79±0.23
Glutaminase liver isoform, mitochondrial	Gls2	3.56±1.50*	1.53±0.29*	0.83±0.31
Keratin, type II cytoskeletal 5	Krt5	3.56±4.11	1.60±2.45	11.14±22.14
Frataxin, mitochondrial	Fxn	3.54±3.31	1.80±1.67	1.97±2.15
Keratin, type I cytoskeletal 17	Krt17	3.50±2.69	1.23±1.59	10.62±21.47
Peroxisomal coenzyme A diphosphatase NUDT7	Nudt7	3.48±2.23*	1.14±0.80	1.12±0.72
Splicing factor 3B subunit 4	Sf3b4	3.46±4.27	3.57±5.19	3.03±4.20
Cytochrome b-c1 complex subunit 10	Uqcr11	3.40±4.36	2.45±3.24	2.47±3.24
Carboxypeptidase B2	Cpb2	3.39±4.66	2.29±2.74	2.39±2.63
Complement component C8 beta chain	C8b	3.39±1.08*	1.07±0.18	0.87±0.30
Beta-2-microglobulin	B2m	3.35±3.11	0.95±0.24	1.31±0.42
Pigment epithelium-derived factor	Serpinf1	3.34±5.88	5.58±10.41	6.03±10.85
Lysosomal acid glucosylceramidase	Gba	3.31±0.46*	1.54±0.52	1.33±0.31
Serine protease inhibitor A3K	Serpina3k	3.28±0.96*	0.88±0.16	0.80±0.13
Coatomer subunit epsilon	Cope	3.27±4.74	2.78±4.10	3.50±5.59
Proteasome subunit beta type-3	Psmb3	3.27±4.30	3.50±5.41	3.56±5.56
Endophilin-B1	Sh3glb1	3.27±5.18	3.47±5.80	3.93±6.42
U8 snoRNA-decapping enzyme	Nudt16	3.26±4.15	3.02±4.24	4.03±5.49
Glycine N-acyltransferase-like protein Keg1	Keg1	3.25±0.94*	1.06±0.23	0.88±0.21
Mevalonate kinase	Mvk	3.22±1.45*	1.24±0.47	1.09±0.35
2-oxo-4-hydroxy-4-carboxy-5-ureidoimidazoline decarboxylase	Urad	3.19±2.07*	0.94±0.20	0.64±0.20
Beta-ureidopropionase	Upb1	3.19±0.80*	1.34±0.51	1.05±0.25
Biotinidase	Btd	3.10±1.91*	1.31±0.72	1.35±0.99

Protein PAT1 homolog 1[#]	Patl1	3.09±2.72	2.74±2.40	4.99±4.23
Signal recognition particle 19 kDa protein	Srp19	3.08±4.09	2.29±3.07	2.46±3.39
Aldehyde oxidase 3	Aox3	3.00±1.58*	1.59±0.49*	1.16±0.25
Epididymis-specific alpha-mannosidase	Man2b2	2.96±1.97	1.64±1.54	1.82±1.75
Cysteine sulfinic acid decarboxylase	Csad	2.91±1.74*	0.50±0.24	1.88±1.14
2-hydroxyacyl-CoA lyase 2	Ilvbl	2.91±4.43	2.82±4.22	2.99±4.38
Pancreatic alpha-amylase	Amy2	2.89±3.48	0.67±0.54	0.85±0.69
Small nuclear ribonucleoprotein E	Snrpe	2.86±3.61	2.22±2.99	2.90±4.12
Galectin-related protein	Lgalsl	2.86±4.63	3.52±5.77	3.26±5.14
Fatty acid-binding protein 5	Fabp5	2.85±1.63*	0.91±0.48	1.11±0.33
Galactose-1-phosphate uridylyltransferase	Galt	2.84±3.21	3.02±4.19	2.14±2.67
Aldose reductase-related protein 2	Akr1b8	2.84±3.75	2.25±2.73	2.75±3.39
Cathepsin F	Ctsf	2.84±1.10*	1.10±0.40	1.11±0.44
Phosphoenolpyruvate carboxykinase, cytosolic [GTP]	Pck1	2.83±1.39*	1.41±0.33*	0.74±0.29
Kynurenine/alpha-aminoadipate aminotransferase, mitochondrial	Aadat	2.82±0.74*	1.28±0.39	0.91±0.29
Acyl-coenzyme A synthetase ACSM1, mitochondrial	Acsm1	2.82±0.86*	1.16±0.32	1.02±0.31
Kynurenine--oxoglutarate transaminase 1	Kyat1	2.76±0.86*	1.00±0.21	0.99±0.20
Keratin, type II cytoskeletal 1b	Krt77	2.74±2.78	0.67±0.42	0.82±0.66
Farnesyl pyrophosphate synthase	Fdps	2.73±1.05*	1.41±0.60	1.07±0.43
Cytochrome P450 2C54	Cyp2c54	2.72±0.49*	0.61±0.19	0.48±0.14
Arylacetamide deacetylase	Aadac	2.70±2.02	1.45±1.19	1.34±1.01
Putative RNA-binding protein Luc7-like 1	Luc7l	2.67±3.04	2.56±3.40	3.20±4.78
Cytochrome b	Mt-Cyb	2.66±2.80	1.83±1.81	1.48±1.03
COMM domain-containing protein 8	Commd8	2.63±2.14	2.16±1.99	2.26±2.73
Cystathionine gamma-lyase	Cth	2.63±0.81*	1.50±0.32*	0.92±0.22
Leukemia inhibitory factor receptor	Lifr	2.63±1.40	1.03±0.23	1.13±0.15
Threonine synthase-like 2	Thnsl2	2.63±0.21*	1.08±0.46	1.13±0.57
Carboxylesterase 3A	Ces3a	2.61±1.04*	0.65±0.14	1.01±0.33
AP-2 complex subunit sigma	Ap2s1	2.61±3.59	2.05±2.57	2.45±3.06
Sodium-coupled neutral amino acid transporter 3	Slc38a3	2.60±1.10*	0.71±0.18	0.63±0.13
Phosphatidylethanolamine N-methyltransferase	Pemt	2.60±3.30	2.02±2.17	1.81±1.56
Alpha-aminoadipic semialdehyde synthase, mitochondrial	Aass	2.60±2.27	1.33±0.59	0.87±0.29
Ornithine carbamoyltransferase, mitochondrial	Otc	2.59±0.85*	1.47±0.52	1.07±0.35
Cytochrome P450 2C70	Cyp2c70	2.58±2.08	1.69±0.78	1.13±0.59
Isovaleryl-CoA dehydrogenase, mitochondrial	Ivd	2.57±0.87*	0.89±0.12	0.74±0.10
Complement component C8 gamma chain	C8g	2.57±2.50	1.29±1.05	1.34±1.25
Cation channel sperm-associated protein subunit beta[#]	Catsperb	2.55±1.47	2.00±0.90	1.42±0.54

Serine--pyruvate aminotransferase, mitochondrial	Agxt	2.55±1.07*	1.08±0.11	0.83±0.15
Cytochrome P450 2C37	Cyp2c37	2.55±1.14*	0.71±0.19	0.73±0.19
NADH-ubiquinone oxidoreductase chain 5	Mtnd5	2.55±3.40	2.44±3.18	2.42±3.25
Apolipoprotein M[#]	Apom	2.55±0.25*	0.75±0.12	0.95±0.21
Retinoid-inducible serine carboxypeptidase	Scpep1	2.53±0.55*	0.80±0.12	0.92±0.21
Aldo-keto reductase family 1 member C18	Akr1c18	2.52±2.51	2.31±2.65	2.74±3.86
Zinc finger and BTB domain-containing protein 20	Zbtb20	2.52±2.14	1.82±1.49	1.33±0.71
Nucleoside diphosphate kinase B	Nme2	2.51±1.07*	1.03±0.20	0.98±0.19
Ig gamma-1 chain C region, membrane-bound form	Ighg1	2.50±2.04	1.28±0.47	1.43±0.90
Phosphopantothenoylcysteine decarboxylase	Ppcdc	2.50±2.07	1.37±0.38	1.30±0.07*
Cytochrome P450 2C44	Cyp2c23	2.50±0.46*	1.09±0.27	0.76±0.12
Cytochrome P450 1A2	Cyp1a2	2.50±0.63*	0.78±0.08	0.95±0.18
Tyrosine aminotransferase	Tat	2.46±0.62*	0.87±0.24	0.72±0.27
4-aminobutyrate aminotransferase, mitochondrial	Abat	2.46±1.39*	1.38±0.75	0.93±0.43
Ancient ubiquitous protein 1	Aup1	2.45±3.38	2.04±2.78	2.00±2.73
Protein CREG1	Creg1	2.45±0.80*	0.96±0.39	1.54±0.79
Ergosterol biosynthetic protein 28 homolog	Erg28	2.44±1.88	1.61±1.46	1.42±1.19
Peptidyl-prolyl cis-trans isomerase NIMA-interacting 1	Pin1	2.43±2.54	2.51±2.90	2.52±2.87
Lanosterol synthase	Lss	2.41±1.16*	1.40±0.98	1.16±0.38
Sterol-4-alpha-carboxylate 3-dehydrogenase, decarboxylating	Nsdhl	2.40±1.33*	1.33±0.85	1.06±0.29
Glycine N-methyltransferase	Gnmt	2.40±1.09*	1.31±0.45	0.85±0.37
Tetratricopeptide repeat protein 39C	Ttc39c	2.40±0.77*	0.86±0.15	0.88±0.13
Arylsulfatase B	Arsb	2.39±0.65*	0.71±0.05	1.03±0.08
Carnitine O-palmitoyltransferase 2, mitochondrial	Cpt2	2.36±0.54*	1.11±0.25	1.12±0.30
CD81 antigen	Cd81	2.36±2.50	1.67±2.05	2.10±2.42
Corrinoid adenosyltransferase	Mmab	2.35±0.52*	1.15±0.23	0.86±0.16
STIP1 homology and U box-containing protein 1	Stub1	2.33±2.80	2.22±2.68	2.07±2.40
Neuroplastin	Nptn	2.33±2.72	2.11±2.67	2.44±3.07
Cytochrome P450 4V2	Cyp4v3	2.32±0.97*	1.05±0.39	1.04±0.39
Protein FAM210B, mitochondrial	Fam210b	2.32±1.67	0.92±0.21	0.96±0.17
Condensin-2 complex subunit D3[#]	Ncapd3	2.31±1.46	1.31±0.95	1.69±0.86
Cytochrome c oxidase subunit 6A1, mitochondrial	Cox6a1	2.30±3.14	1.96±2.32	1.93±2.32
Nuclear cap-binding protein subunit 1	Ncbp1	2.29±2.99	2.41±3.23	2.54±3.24
Nascent polypeptide-associated complex subunit alpha, muscle-specific form	Naca	2.29±2.60	2.15±2.63	2.13±2.58
Mitogen-activated protein kinase kinase 11[#]	Map3k11	2.28±0.43*	1.17±0.31	1.30±0.35
Delta(14)-sterol reductase TM7SF2	Tm7sf2	2.28±0.76*	0.85±0.18	0.78±0.20
CDGSH iron-sulfur domain-containing protein 1	Cisd1	2.27±1.41	1.61±1.32	1.45±1.04

Pro-cathepsin H	Ctsh	2.27±0.47*	1.09±0.25	1.41±0.50
NAD-dependent malic enzyme, mitochondrial	Me2	2.24±2.73	1.95±2.35	2.54±3.24
Cytochrome P450 2F2	Cyp2f2	2.23±1.27	1.64±0.98	1.18±0.74
WD repeat-containing protein 18	Wdr18	2.22±2.64	2.18±2.66	2.03±2.17
Cadherin EGF LAG seven-pass G-type receptor 3#	Celsr3	2.21±1.48	1.78±1.60	1.65±1.18
Endonuclease G, mitochondrial	Endog	2.21±1.64	1.56±1.14	1.40±1.03
Keratin, type I cytoskeletal 28	Krt28	2.21±3.17	0.81±0.55	1.74±1.37
Acylcarnitine hydrolase	Ces2c	2.20±0.95*	0.93±0.09	0.92±0.09
Sarcosine dehydrogenase, mitochondrial	Sardh	2.19±0.59*	1.45±0.33*	0.95±0.24
ER membrane protein complex subunit 7	Emc7	2.17±2.81	1.88±2.23	1.92±2.37
Beta-mannosidase	Manba	2.16±1.19	0.71±0.09	0.86±0.30
Alpha-N-acetylgalactosaminidase	Naga	2.16±0.36*	0.89±0.07	1.06±0.17
Keratin, type II cytoskeletal 2 epidermal	Krt2	2.15±1.84	1.47±1.86	4.58±8.23
Kynurenine 3-monooxygenase	Kmo	2.14±2.47	1.74±1.64	1.34±1.22
Glycerophosphocholine phosphodiesterase GPCPD1	Gpcpd1	2.14±1.59	0.79±0.39	0.70±0.35
ATP synthase subunit f, mitochondrial	Atp5mf	2.13±2.20	1.94±1.94	1.80±2.03
Small nuclear ribonucleoprotein Sm D1	Snrpd1	2.13±1.84	2.05±2.04	2.03±2.00
CD302 antigen	Cd302	2.12±1.72	0.86±0.49	1.08±0.91
Parathymosin	Ptms	2.12±1.66	1.92±1.69	1.68±1.45
Hydroxyacid oxidase 1	Hao1	2.11±0.90*	0.99±0.45	1.15±0.34
Histidine triad nucleotide-binding protein 2, mitochondrial	Hint2	2.10±0.35*	0.99±0.14	0.93±0.18
Pyridoxine-5'-phosphate oxidase	Pnpo	2.10±0.67*	1.23±0.16*	1.00±0.04
Protein-glutamine gamma-glutamyltransferase K	Tgm1	2.10±0.62*	0.82±0.27	0.91±0.20
Serine hydrolase-like protein#	Serhl	2.10±2.09	1.94±1.62	2.05±1.88
Mitochondrial chaperone BCS1	Bcs1l	2.09±1.82	1.65±1.32	1.54±1.28
Ubiquitin-like modifier-activating enzyme ATG7	Atg7	2.07±1.35	1.38±0.73	1.41±0.88
Argininosuccinate synthase	Ass1	2.05±1.64	1.92±0.75*	0.90±0.38
Acyl-CoA-binding protein	Dbi	2.05±2.30	2.20±3.02	2.05±2.43
UDP-glucuronosyltransferase 1-1	Ugt1a1	2.05±1.31	1.34±0.88	1.32±0.83
Acetyl-coenzyme A transporter 1	Slc33a1	2.04±2.75	2.20±2.51	2.13±2.62
Phenylalanine-4-hydroxylase	Pah	2.03±1.32	1.42±0.79	1.21±0.73
Regucalcin	Rgn	2.03±0.80*	1.08±0.59	0.82±0.31
Arginase-1	Arg1	2.02±0.50*	1.21±0.25	0.97±0.23
Keratin, type I cytoskeletal 16	Krt16	2.02±1.83	0.80±0.58	4.30±6.06
S-methylmethionine--homocysteine S-methyltransferase BHMT2	Bhmt2	2.01±2.67	2.51±3.26	2.70±3.60
Medium-chain acyl-CoA ligase ACSF2, mitochondrial	Acsf2	2.01±0.59*	0.89±0.14	1.05±0.21
Glutathione S-transferase Mu 7	Gstm7	2.00±1.36	1.79±1.50	1.47±1.11

Mitochondrial import receptor subunit TOM5 homolog	Tomm5	2.00±2.27	1.97±2.01	1.93±2.13

These values represent average (± standard deviation) fold-change of abundance ratios for each altered protein in C57BL6 mice on low-fat compared to the high-fat control group (MS-NASH mice on high-fat) with a cutoff of 2-fold-change. For each upregulated protein, corresponding values from the other two groups (on high-fat diet) are shown for comparison. The liver samples (from 5 mice in each group) were individually assessed by TMT based differential proteomic expression and data were merged to get averages. (*) represents significance (p-value <0.05) as compared to the high-fat mice. Protein FDR Confidence for all proteins was $\leq$1% except 8 proteins ($^{\#}\leq$2%). These data are also presented in Figure 3A. FDR: False Discovery Rate.
