# Supplementary material for: Liver Protein Expression in NASH Mice on a High-Fat Diet: Response to Multi-Mineral Intervention
Source: Front Nutr. 2022 May 11;9:859292. doi: 10.3389/fnut.2022.859292 (PMC9130755; doi:10.3389/fnut.2022.859292)
Supplement: Supplementary Table 1 — Mineral Composition of Aquamin® Soluble. [file Data_Sheet_1.zip › SM Table 11 859292.pdf]

Supplement Table 11. Downregulated Proteins by an unbiased proteomic screening of C57BL6 mice on low-fat diet

Proteins	Genes	C57BL6	MS-NASH	
		Control	OCA	Aquamin
Leucine-rich repeat-containing protein 9[#]	Lrrc9	0.046±0.013*	0.978±0.235	0.705±0.168*
Histone H2A type 2-A	Hist2h2aa1	0.181±0.229*	0.878±0.500	0.941±0.590
Cytochrome P450 2B9	Cyp2b9	0.182±0.068*	0.751±0.219*	1.076±0.412
Perilipin-2	Plin2	0.184±0.013*	0.559±0.096*	0.864±0.107*
Cornifin-A	Sprr1a	0.207±0.148*	0.920±0.570	1.341±0.634
Thymidine phosphorylase	Tymp	0.216±0.038*	1.633±0.324*	0.939±0.160
Lymphocyte antigen 6D	Ly6d	0.221±0.094*	1.116±0.828	1.693±0.866
Histone H2B type 1-B	H2bc3	0.227±0.251*	0.840±0.449	0.931±0.614
SCY1-like protein 2	Scyl2	0.231±0.131*	1.032±0.144	1.028±0.080
Son of sevenless homolog 1[#]	Sos1	0.233±0.096*	0.605±0.115*	1.015±0.134
Keratin, type I cytoskeletal 20	Krt20	0.236±0.167*	0.925±0.343	0.971±0.256
PDZ and LIM domain protein 7	Pdlim7	0.241±0.121*	0.804±0.342	1.123±0.265
Branched-chain-amino-acid aminotransferase, mitochondrial	Bcat2	0.250±0.211*	0.604±0.594	0.656±0.549
Oxysterol-binding protein-related protein 3	Osbpl3	0.255±0.087*	0.627±0.467	1.297±0.707
Histone H2AX	H2ax	0.257±0.253*	0.931±0.515	1.056±0.712
Centrosomal protein of 170 kDa	Cep170	0.260±0.244*	0.461±0.341	0.672±0.529
Perilipin-4	Plin4	0.270±0.170*	0.502±0.264*	1.445±1.157
Lymphocyte-specific protein 1	Lsp1	0.272±0.136*	0.798±0.420	1.091±0.551
Myosin regulatory light polypeptide 9	Myl9	0.274±0.056*	0.936±0.603	0.939±0.406
Aquaporin-4	Aqp4	0.285±0.197*	3.992±3.087	0.921±0.561
Cysteine and glycine-rich protein 1	Csrp1	0.292±0.208*	0.695±0.207*	1.097±0.307
Histone H2A.V	H2az2	0.295±0.264*	0.993±0.330	1.076±0.663
Acyl-CoA desaturase 1	Scd1	0.296±0.141*	0.720±0.322	0.828±0.212
Trinucleotide repeat-containing gene 6A protein[#]	Tnrc6a	0.304±0.193*	0.663±0.455	0.809±0.607
Cytochrome P450 2C38	Cyp2c38	0.305±0.177*	1.332±0.317	1.084±0.283
Apolipoprotein C-II	Apoc2	0.311±0.147*	1.155±0.593	1.180±0.433
Elongation of very long chain fatty acids protein 5	Elovl5	0.311±0.087*	1.058±0.222	0.965±0.239
Interferon alpha-inducible protein 27-like protein 2B	Ifi27l2b	0.333±0.070*	0.909±0.194	1.124±0.310
Tonsoku-like protein	Tonsl	0.342±0.232*	0.306±0.172*	0.512±0.333
2'-deoxynucleoside 5'-phosphate N-hydrolase 1	Dnph1	0.344±0.214*	1.428±0.860	0.897±0.489
Brain acid soluble protein 1	Basp1	0.347±0.101*	0.864±0.223	1.184±0.333
Histone H2A type 1-F	Hist1h2af	0.355±0.388*	0.647±0.432	0.818±0.511
Fatty acid synthase	Fasn	0.356±0.145*	1.501±0.692	1.171±0.425

Protein ABHD1	Abhd1	0.356±0.146*	0.995±0.116	0.981±0.165
Uridine-cytidine kinase 1	Uck1	0.362±0.083*	0.695±0.077*	0.928±0.119
Casein kinase I isoform alpha	Csnk1a1	0.364±0.271*	0.972±0.207	0.892±0.140
Protein phosphatase 1 regulatory subunit 12C	Ppp1r12c	0.368±0.297*	0.870±0.098	0.908±0.144
Synaptopodin[#]	Synpo	0.368±0.036*	0.812±0.351	1.250±0.587
Regulator of G-protein signaling 10	Rgs10	0.369±0.196*	0.856±0.278	1.296±0.198
Cytochrome P450 2C39	Cyp2c39	0.373±0.132*	1.365±0.175	1.029±0.185
Emopamil-binding protein-like	Ebpl	0.376±0.212*	0.724±0.108*	0.867±0.125*
Histone H4	H4c1	0.378±0.410*	0.851±0.341	0.936±0.620
Plastin-1	Pls1	0.380±0.345*	1.401±0.599	1.398±0.404
Glycine amidinotransferase, mitochondrial	Gatm	0.387±0.211*	0.732±0.431	0.876±0.486
Death-associated protein 1	Dap	0.389±0.146*	1.013±0.459	0.924±0.470
Annexin A2	Anxa2	0.390±0.119*	0.826±0.272	1.313±0.171
Diacylglycerol O-acyltransferase 1	Dgat1	0.392±0.319*	0.835±0.266	0.999±0.428
Histone H3.1	H3c1	0.392±0.418*	0.791±0.422	0.936±0.741
Monoglyceride lipase	Mgll	0.394±0.079*	0.767±0.123*	0.942±0.151
Protein LSM12 homolog	Lsm12	0.394±0.257*	0.954±0.108	1.058±0.202
Ena/VASP-like protein	Evl	0.397±0.194*	0.927±0.705	1.309±0.750
STE20/SPS1-related proline-alanine-rich protein kinase	Stk39	0.398±0.315*	0.885±0.634	1.204±0.786
Heme-binding protein 1	Hebp1	0.398±0.064*	1.110±0.264	0.989±0.193
Histone H3.2	H3c2	0.399±0.280*	0.884±0.193	0.960±0.032
Acyl-coenzyme A thioesterase 2, mitochondrial	Acot2	0.400±0.123*	1.022±0.290	1.289±0.436
Collagen alpha-1(XXV) chain[#]	Col25a1	0.402±0.221*	0.915±0.334	0.903±0.369
Annexin A1	Anxa1	0.403±0.156*	0.716±0.208*	1.037±0.381
Triokinase/FMN cyclase	Tkfc	0.404±0.099*	1.472±0.355	1.213±0.317
Sorbin and SH3 domain-containing protein 1	Sorbs1	0.410±0.092*	0.895±0.123	0.923±0.226
Acyl-coenzyme A thioesterase 11	Acot11	0.412±0.058*	0.924±0.133	0.980±0.240
17-beta-hydroxysteroid dehydrogenase 13	Hsd17b13	0.412±0.177*	0.916±0.286	1.256±0.304
ATP-citrate synthase	Acly	0.413±0.098*	1.367±0.295	1.065±0.253
Charged multivesicular body protein 2b	Chmp2b	0.416±0.219*	1.072±0.156	1.378±0.229
Pyridine nucleotide-disulfide oxidoreductase domain-containing protein 2	Pyroxd2	0.420±0.324*	1.061±0.076	0.950±0.025*
Acetyl-CoA carboxylase 1	Acaca	0.420±0.111*	1.152±0.300	1.096±0.188
Fatty acid-binding protein, intestinal	Fabp2	0.421±0.136*	1.233±0.062	1.128±0.067
Apolipoprotein A-IV	Apoa4	0.423±0.125*	1.238±0.432	1.706±0.733
Quinone oxidoreductase-like protein 1	Cryzl1	0.423±0.299*	0.650±0.491	0.514±0.430
Sterile alpha motif domain-containing protein 9-like	Samd9l	0.426±0.077*	1.178±0.168	1.415±0.144*
High mobility group protein HMG-I/HMG-Y	Hmga1	0.426±0.209*	1.460±0.331	1.105±0.231

Rho guanine nucleotide exchange factor 2	Arhgef2	0.427±0.135*	0.744±0.358	1.205±0.350
Acyl-CoA synthetase short-chain family member 3, mitochondrial	Acss3	0.429±0.111*	1.082±0.234	1.083±0.400
Histone H1.5	H1-5	0.430±0.145*	1.010±0.181	1.211±0.266
ATP-binding cassette sub-family D member 1	Abcd1	0.430±0.161*	0.942±0.180	0.934±0.212
Transmembrane protein 230	Tmem230	0.431±0.199*	0.877±0.186	0.867±0.280
Peroxisomal bifunctional enzyme	Ehhadh	0.431±0.168*	0.858±0.240	1.110±0.395
Galectin-3	Lgals3	0.434±0.161*	0.840±0.280	1.430±0.391
Zyxin	Zyx	0.435±0.053*	1.089±0.210	1.076±0.150
FYN-binding protein 2	Fyb2	0.436±0.158*	1.275±0.175	1.055±0.305
Lysophosphatidylcholine acyltransferase 1	Lpcat1	0.437±0.221*	1.094±0.292	1.053±0.332
NudC domain-containing protein 3	Nudcd3	0.439±0.150*	0.913±0.092	1.076±0.147
D-3-phosphoglycerate dehydrogenase	Phgdh	0.440±0.375	0.551±0.464	0.690±0.600
Melanoma-associated antigen D1	Maged1	0.441±0.184*	1.002±0.422	1.043±0.639
Prostaglandin reductase 1	Ptgr1	0.443±0.093*	0.847±0.153	1.123±0.187
Epididymal-specific lipocalin-10#	Lcn10	0.444±0.466	0.918±0.787	0.745±0.695
Helicase-like transcription factor	Hltf	0.445±0.168*	0.946±0.458	0.890±0.079
Histone H3.3	H3-3a	0.449±0.505*	0.875±0.463	0.894±0.586
Myosin regulatory light chain 12B	Myl12b	0.450±0.127*	1.029±0.255	1.131±0.215
CTTNBP2 N-terminal-like protein	Cttnbp2nl	0.453±0.151*	0.832±0.154	1.035±0.263
Bromodomain-containing protein 3	Brd3	0.453±0.068*	1.041±0.254	0.856±0.349
WAS/WASL-interacting protein family member 3	Wipf3	0.454±0.107*	1.289±0.481	1.201±0.559
Endonuclease 8-like 2	Neil2	0.455±0.255*	1.209±0.434	1.231±0.724
Sulfotransferase 1C2	Sult1c2	0.455±0.135*	0.984±0.371	1.092±0.407
Reticulon-4	Rtn4	0.456±0.107*	0.793±0.107*	1.108±0.178
A-kinase anchor protein 2	Akap2	0.456±0.068*	0.833±0.065*	1.004±0.197
Sphingomyelin phosphodiesterase 3	Smpd3	0.459±0.153*	1.224±0.148*	0.972±0.253
Apolipoprotein A-II	Apoa2	0.459±0.258*	0.651±0.183*	1.074±0.327
Galectin-1	Lgals1	0.461±0.192*	0.488±0.266*	1.038±0.387
Phosphatidylethanolamine-binding protein 1	Pebp1	0.461±0.204*	1.263±0.771	1.378±0.930
Serine/threonine-protein phosphatase 6 regulatory subunit 1	Ppp6r1	0.462±0.225*	1.016±0.279	0.975±0.220
Microtubule-associated protein 4	Map4	0.462±0.115*	0.955±0.142	1.120±0.275
Rho GTPase-activating protein 23	Arhgap23	0.463±0.096*	0.767±0.135*	1.155±0.152
85/88 kDa calcium-independent phospholipase A2	Pla2g6	0.464±0.115*	1.045±0.221	0.908±0.333
PDZ domain-containing protein GIPC1	Gipc1	0.466±0.110*	0.904±0.140	1.009±0.210
Collagen alpha-1(XIV) chain	Col14a1	0.466±0.036*	1.155±0.543	1.815±0.964
Adenylate kinase isoenzyme 1	Ak1	0.467±0.392	0.656±0.522	0.812±0.649
H-2 class II histocompatibility antigen gamma chain	Cd74	0.470±0.182*	0.971±0.774	2.733±3.743

Zinc finger CCCH domain-containing protein 11A	Zc3h11a	0.471±0.214*	0.939±0.278	1.075±0.352
Uncharacterized protein KIAA1522	Kiaa1522	0.474±0.279*	0.994±0.278	1.207±0.614
Microtubule-associated protein tau	Mapt	0.475±0.132*	0.960±0.315	1.776±0.814
Protein S100-A10	S100a10	0.476±0.073*	0.857±0.361	1.353±0.302
Glucose-6-phosphatase	G6pc	0.476±0.098*	0.801±0.156*	0.893±0.173
Arf-GAP domain and FG repeat-containing protein 1	Agfg1	0.478±0.260*	0.957±0.291	0.951±0.309
GRB10-interacting GYF protein 2	Gigyf2	0.479±0.148*	0.979±0.225	0.972±0.339
Actin-binding LIM protein 1	Ablim1	0.480±0.137*	0.987±0.071	0.990±0.125
Testis-expressed protein 2	Tex2	0.482±0.124*	0.956±0.113	0.933±0.158
Band 4.1-like protein 1	Epb41l1	0.485±0.163*	1.628±0.540	1.236±0.474
Neuronal proto-oncogene tyrosine-protein kinase Src	Src	0.486±0.515	1.418±1.088	1.441±0.757
Glycogen synthase kinase-3 alpha	Gsk3a	0.489±0.280*	1.191±0.427	1.233±0.208
Nucleoporin NUP35	Nup35	0.492±0.205*	0.930±0.359	0.900±0.226
Protein farnesyltransferase subunit beta	Fntb	0.493±0.362	0.684±0.446	0.616±0.428
Peroxisomal membrane protein 11A	Pex11a	0.493±0.078*	0.657±0.092*	0.957±0.034*
Coronin-1A	Coro1a	0.494±0.210*	0.798±0.389	1.131±0.466
ATP-binding cassette sub-family D member 2	Abcd2	0.495±0.065*	0.924±0.148	1.236±0.287
Serpin H1	Serpinh1	0.497±0.208*	0.819±0.171*	1.141±0.224
Proteolipid protein 2#	Plp2	0.498±0.393*	0.644±0.364	1.007±0.478

These values represent average (± standard deviation) fold-change of abundance ratios for each altered protein in C57BL6 mice on low-fat compared to the high-fat control group (MS-NASH mice on high-fat) with a cutoff of 2-fold-change. For each downregulated protein, corresponding values from the other two groups (on high-fat diet) are shown for comparison. The liver samples (from 5 mice in each group) were individually assessed by TMT based differential proteomic expression and data were merged to get averages. (*) represents significance (p-value <0.05) as compared to the high-fat mice. Protein FDR Confidence for all proteins was ≤1% except 7 proteins (#≤2%). These data are also presented in Figure 3A. FDR: False Discovery Rate
