# Supplementary material for: Liver Protein Expression in NASH Mice on a High-Fat Diet: Response to Multi-Mineral Intervention
Source: Front Nutr. 2022 May 11;9:859292. doi: 10.3389/fnut.2022.859292 (PMC9130755; doi:10.3389/fnut.2022.859292)
Supplement: Supplementary Table 1 — Mineral Composition of Aquamin® Soluble. [file Data_Sheet_1.zip › SM Table 12 859292.pdf]

Supplement Table 12. Top pathways associated with upregulated proteins altered with low-fat diet in C57BL6 mice

Pathway name	Entities pValue	Mapped entities
Metabolism	4.84×10^{-10}	Apom;Ces3b;Otc;Kmo;Ak4;Hao1;Nsdhl;Aass;Nme2;Lss;Glrx;Ass1;Rap1a;Akr1b8;Ugt3a2;Cps1;Gnmt;Cyp1a2;Fdps;Cyp2f2;Mmab;Gba;Ivd;Ugt1a9;Gls2;Cyp51a1;Cyp7b1;Mvk;Me2;Hoga1;Msmo1;Isyna1;Arsb;Aadat;Abcd4;Pck1;Cox6a1;Kyat1;Aadac;Acsf2;Nudt16;Cyp4v2;Mtnd5;Atp5mf;Sardh;Gstm7;Psmb3;Pnpo;Gstp1;Arg1;Man2b2;Fxn;Agxt;Idi1;Galt;Ugt1a1;Ppcdc;Upb1;Cpt2;Manba;Gcsh;Cth;Dbi;Acsm1;Fabp5;Pah;Btd;Tm7sf2;Mt-Cyb;Pemt;Bhmt2;Tat
Metabolism of amino acids and derivatives	4.63×10^{-8}	Gnmt;Sardh;Kmo;Otc;Psmb3;Hao1;Gcsh;Ivd;Arg1;Aass;Gls2;Cth;Hoga1;Agxt;Pah;Aadat;Ass1;Bhmt2;Tat;Kyat1;Cps1
Cholesterol biosynthesis	5.63×10^{-7}	Msmo1; Lss;Fdps;Idi1;Tm7sf2;Nsdhl;Cyp51a1;Mvk
Formation of the cornified envelope	2.61×10^{-7}	Krt2; Krt1;Krt79;Krt16;Tgm1;Krt5;Krt76;Krt17;Krt10; Krt14;Krt77;Krt28
Urea cycle	5.62×10^{-5}	Ass1;Otc;Arg1;Cps1
Metabolism of steroids	1.58×10^{-4}	Msmo1; Lss;Fdps;Idi1;Tm7sf2;Akr1b8;Nsdhl;Cyp51a1;Cyp7b1;Mvk
Keratinization	2.03×10^{-4}	Krt2; Krt1;Krt79;Krt16;Tgm1;Krt5;Krt76;Krt17;Krt10; Krt14;Krt77;Krt28
Biological oxidations	3.84×10^{-4}	Cyp1a2;Ugt1a1;Cyp4v2;Ces3b;Gstm7;Cyp2f2;Gstp1;Ugt1a9;Cyp51a1;Cyp7b1;Acsm1;Ugt3a2;Aadac
Glyoxylate metabolism and glycine degradation	3.87×10^{-4}	Gnmt;Hoga1;Agxt;Hao1;Gcsh
Phenylalanine and tyrosine metabolism	0.002	Pah;Tat;Kyat1
Tryptophan catabolism	0.002	Aadat;Kmo;Kyat1
Lysosomal oligosaccharide catabolism	0.003	Man2b2;Manba
Phase II - Conjugation of compounds	0.004	Cyp1a2;Ugt1a1;Gstm7;Gstp1;Ugt1a9;Ugt3a2;Acsm1
Phenylalanine metabolism	0.007	Pah;Kyat1
Phase I - Functionalization of compounds	0.007	Cyp1a2;Cyp4v2;Ces3b;Cyp2f2;Cyp51a1;Aadac;Cyp7b1
Glucuronidation	0.010	Ugt1a1;Ugt1a9;Ugt3a2
Terminal pathway of complement	0.012	C8b;C8g
Cytochrome P450 - arranged by substrate type	0.016	Cyp1a2;Cyp4v2;Cyp2f2;Cyp51a1;Cyp7b1
SLBP independent Processing of Histone Pre-mRNAs	0.018	Ncbp1;Snrpe
Mitochondrial protein import	0.018	Fxn;Otc
Endogenous sterols	0.018	Cyp4v2;Cyp51a1;Cyp7b1
Interconversion of nucleotide di- and triphosphates	0.019	Nme2;Glrx;Ak4
Aromatic amines can be N-hydroxylated or N-dealkylated by CYP1A2	0.020	Cyp1a2

SLBP Dependent Processing of Replication-Dependent Histone Pre-mRNAs	0.021	Ncbp1;Snrpe
Type I hemidesmosome assembly	0.021	Krt5; Krt14
mRNA Splicing - Minor Pathway	0.022	Sf3b4;Ncbp1;Snrpe;Snrpd1
Lysine catabolism	0.025	Aadat;Aass
Cobalamin (Cbl, vitamin B12) transport and metabolism	0.025	Mmab;Abcd4
Glutamate and glutamine metabolism	0.029	Gls2;Kyat1
Conjugation of phenylacetate with glutamine	0.039	Acsm1
Cysteine formation from homocysteine	0.039	Cth
Degradation of GABA	0.039	Abat
Developmental Biology	0.045	Krt1;Krt16;Tgm1;Rras;Krt76; Krt14;Krt77;Krt2;Egfr;Krt79;Krt5;Tubb4a;Krt17;Krt10;Krt28;Ap2s1
Metabolism of nucleotides	0.046	Nudt16;Nme2;Glrx;Upb1;Ak4

The pathways listed here are altered by the significantly upregulated proteins with the control low-fat diet in C57BL6 mice presented in Supplement Table 10 using an unbiased approach. Reactome (v78) was used to generate the pathway analysis report for species *Mus musculus*. The significance (*p*-value) is calculated by the overrepresentation analysis (hypergeometric distribution).
