# Supplementary material for: Liver Protein Expression in NASH Mice on a High-Fat Diet: Response to Multi-Mineral Intervention
Source: Front Nutr. 2022 May 11;9:859292. doi: 10.3389/fnut.2022.859292 (PMC9130755; doi:10.3389/fnut.2022.859292)
Supplement: Supplementary Table 1 — Mineral Composition of Aquamin® Soluble. [file Data_Sheet_1.zip › SM Table 13 859292.pdf]

Supplement Table 13. Top pathways associated with downregulated proteins altered with low-fat diet in C57BL6 mice

Pathway name	Entities pValue	Mapped entities
NoRC negatively regulates rRNA expression	1.27×10^{-9}	H2ax;H3c1;Hist2h2aa1;H4c1;H3-3;H2az2;H2bc3;H3c2;Hist1h2af
SIRT1 negatively regulates rRNA expression	2.11×10^{-9}	H2ax;H3c1;Hist2h2aa1;H4c1;H3-3;H2az2;H2bc3;H3c2;Hist1h2af
RNA Polymerase I Promoter Opening	2.11×10^{-9}	H2ax;H3c1;Hist2h2aa1;H4c1;H3-3;H2az2;H2bc3;H3c2;Hist1h2af
Negative epigenetic regulation of rRNA expression	2.69×10^{-9}	H2ax;H3c1;Hist2h2aa1;H4c1;H3-3;H2az2;H2bc3;H3c2;Hist1h2af
Activated PKN1 stimulates transcription of AR (androgen receptor) regulated genes KLK2 and KLK3	3.40×10^{-9}	H2ax;H3c1;Hist2h2aa1;H4c1;H3-3;H2az2;H2bc3;H3c2;Hist1h2af
Assembly of the ORC complex at the origin of replication	8.18×10^{-9}	H2ax;H3c1;Hist2h2aa1;H4c1;H3-3;H2az2;H2bc3;H3c2;Hist1h2af
Transcriptional regulation by small RNAs	1.59×10^{-8}	H2ax;H3c1;Hist2h2aa1;H4c1;H3-3;H2az2;H2bc3;Q3UHK8;H3c2;Hist1h2af;Nup35
PRC2 methylates histones and DNA	1.80×10^{-8}	H2ax;H3c1;Hist2h2aa1;H4c1;H3-3;H2az2;H2bc3;H3c2;Hist1h2af
Gene Silencing by RNA	4.75×10^{-8}	H2ax;H3c1;Hist2h2aa1;H4c1;H3-3;H2az2;H2bc3;Q3UHK8;H3c2;Hist1h2af;Nup35
RUNX1 regulates genes involved in megakaryocyte differentiation and platelet function	8.39×10^{-8}	H2ax;H3c1;Hist2h2aa1;H4c1;H3-3;H2az2;H2bc3;H3c2;Hist1h2af
RHO GTPases activate PKNs	1.14×10^{-7}	H2ax;H3c1;Hist2h2aa1;H4c1;H3-3;H2az2;H2bc3;H3c2;Hist1h2af
RNA Polymerase I Promoter Escape	2.65×10^{-7}	H2ax;H3c1;Hist2h2aa1;H4c1;H3-3;H2az2;H2bc3;H3c2;Hist1h2af
Positive epigenetic regulation of rRNA expression	2.65×10^{-7}	H2ax;H3c1;Hist2h2aa1;H4c1;H3-3;H2az2;H2bc3;H3c2;Hist1h2af
B-WICH complex positively regulates rRNA expression	2.65×10^{-7}	H2ax;H3c1;Hist2h2aa1;H4c1;H3-3;H2az2;H2bc3;H3c2;Hist1h2af
Senescence-Associated Secretory Phenotype (SASP)	4.44×10^{-7}	H2ax;H3c1;Hist2h2aa1;H4c1;H3-3;H2az2;H2bc3;H3c2;Hist1h2af
Oxidative Stress Induced Senescence	1.74×10^{-7}	H2ax;H3c1;Hist2h2aa1;H4c1;H3-3;H2az2;H2bc3;H3c2;Hist1h2af
RNA Polymerase I Promoter Clearance	1.93×10^{-6}	H2ax;H3c1;Hist2h2aa1;H4c1;H3-3;H2az2;H2bc3;H3c2;Hist1h2af
Epigenetic regulation of gene expression	2.13×10^{-6}	H2ax;H3c1;Hist2h2aa1;H4c1;H3-3;H2az2;H2bc3;H3c2;Hist1h2af
RNA Polymerase I Transcription	2.13×10^{-6}	H2ax;H3c1;Hist2h2aa1;H4c1;H3-3;H2az2;H2bc3;H3c2;Hist1h2af
DNA Damage/Telomere Stress Induced Senescence	2.96×10^{-6}	H2ax;Hist2h2aa1;H4c1;Hmga1;H2az2;H1-5;H2bc3;Hist1h2af

Cellular Senescence	3.41×10^{-6}	H2ax;H3c1;Hist2h2aa1;H4c1;H3-3;Hmga1;H2az2;H1-5;H2bc3;H3c2;Hist1h2af
Base-Excision Repair, AP Site Formation	4.35×10^{-6}	H2ax;Hist2h2aa1;H4c1;H2az2;Q6R2P8;H2bc3;Hist1h2af
Estrogen-dependent gene expression	5.98×10^{-6}	H2ax;H3c1;Hist2h2aa1;H4c1;H3-3;H2az2;H2bc3;H3c2;Hist1h2af
Assembly of the pre-replicative complex	1.36×10^{-5}	H2ax;H3c1;Hist2h2aa1;H4c1;H3-3;H2az2;H2bc3;H3c2;Hist1h2af
Inhibition of DNA recombination at telomere	1.51×10^{-5}	H2ax;Hist2h2aa1;H4c1;H2az2;H2bc3;Hist1h2af
Recognition and association of DNA glycosylase with site containing an affected purine	1.51×10^{-5}	H2ax;Hist2h2aa1;H4c1;H2az2;H2bc3;Hist1h2af
Depurination	2.02×10^{-5}	H2ax;Hist2h2aa1;H4c1;H2az2;H2bc3;Hist1h2af
Cleavage of the damaged purine	2.02×10^{-5}	H2ax;Hist2h2aa1;H4c1;H2az2;H2bc3;Hist1h2af
Condensation of Prophase Chromosomes	3.05×10^{-5}	H2ax;Hist2h2aa1;H4c1;H2az2;H2bc3;Hist1h2af
RHO GTPase Effectors	4.21×10^{-5}	H2ax;Myl9;Hist2h2aa1;H4c1;H2bc3;Myl12b;Evl;H3c1;Src;H3-3;H2az2;H3c2;Hist1h2af;Wipf3
DNA Replication Pre-Initiation	4.25×10^{-5}	H2ax;H3c1;Hist2h2aa1;H4c1;H3-3;H2az2;H2bc3;H3c2;Hist1h2af
Base Excision Repair	8.80×10^{-5}	H2ax;Hist2h2aa1;H4c1;H2az2;Q6R2P8;H2bc3;Hist1h2af
ESR-mediated signaling	8.88×10^{-5}	H2ax;H3c1;Hist2h2aa1;H4c1;H3-3;Src;H2az2;H2bc3;H3c2;Hist1h2af
Nucleosome assembly	1.61×10^{-4}	H2ax;Hist2h2aa1;H4c1;H2az2;H2bc3;Hist1h2af
Deposition of new CENPA-containing nucleosomes at the centromere	1.61×10^{-4}	H2ax;Hist2h2aa1;H4c1;H2az2;H2bc3;Hist1h2af
HDACs deacetylate histones	2.12×10^{-4}	H3c1;Hist2h2aa1;H4c1;H2bc3;H3c2;Hist1h2af
Chylomicron remodeling	3.96×10^{-4}	Apoa2;Apoa4;Apoc2
Chylomicron assembly	3.96×10^{-4}	Apoa2;Apoa4;Apoc2
DNA Replication	4.32×10^{-4}	H2ax;H3c1;Hist2h2aa1;H4c1;H3-3;H2az2;H2bc3;H3c2;Hist1h2af
Mitotic Prophase	4.78×10^{-4}	H2ax;Hist2h2aa1;H4c1;H2az2;H2bc3;Hist1h2af;Nup35
Transcriptional regulation by RUNX1	5.38×10^{-4}	H2ax;H3c1;Hist2h2aa1;H4c1;H3-3;H2az2;H2bc3;H3c2;Hist1h2af
RMTs methylate histone arginines	6.01×10^{-4}	H3c1;Hist2h2aa1;H4c1;H3c2;Hist1h2af
Signaling by Nuclear Receptors	7.81×10^{-4}	H2ax;H3c1;Hist2h2aa1;H4c1;H3-3;Src;H2az2;H2bc3;H3c2;Hist1h2af
Telomere Maintenance	9.49×10^{-4}	H2ax;Hist2h2aa1;H4c1;H2az2;H2bc3;Hist1h2af
Fatty acyl-CoA biosynthesis	0.001	Acly;Fasn;Acaca;Elovl5
Plasma lipoprotein assembly	0.002	Apoa2;Apoa4;Apoc2
Signaling by Rho GTPases	0.002	H2ax;Arhgef2;Myl9;Hist2h2aa1;H4c1;H2bc3;Myl12b;Sos1;Evl;H3c1;Src;H3-3;H2az2;Tex2;Arhgap23;H3c2;Hist1h2af;Basp1;Wipf3
Fatty acid metabolism	0.003	Ehhadh;Acot11;Acly;Fasn;Abcd1;Acaca;Acly;Elovl5

Signaling by Rho GTPases, Miro GTPases and RHOBTB3	0.003	H2ax;Arhgef2;Myl9;Hist2h2aa1;H4c1;H2bc3;Myl12b;Sos1;Evl;H3c1;Src;H3-3;H2az2;Tex2;Arhgap23;H3c2;Hist1h2af;Basp1;Wipf3
HATs acetylate histones	0.003	H3c1;H4c1;H2bc3;H3c2
ChREBP activates metabolic gene expression	0.003	Fasn;Acaca
Chromosome Maintenance	0.004	H2ax;Hist2h2aa1;H4c1;H2az2;H2bc3;Hist1h2af
Metabolism of lipids	0.004	Hsd17b13;Fasn;Abcd1;Acaca;Lpcat1;Mgll;Ehhadh;Pla2g6;Dgat1;Acot11;Acly;Acly;Osbpl3;Elovl5;Smpd3;Acss3;Fabp2
Acyl chain remodeling of DAG and TAG	0.004	Mgll;Dgat1
Linoleic acid (LA) metabolism	0.006	Abcd1;Elovl5
HDMs demethylate histones	0.006	H3c1;H4c1;H3c2
Plasma lipoprotein remodeling	0.007	Apoa2;Apoa4;Apoc2
Smooth Muscle Contraction	0.009	Sorbs1;Myl9;Myl12b
Beta-oxidation of very long chain fatty acids	0.011	Ehhadh;Abcd1
Pyrimidine salvage	0.011	Uck1;Tymp
Formyl peptide receptors bind formyl peptides and many other ligands	0.012	Anxa1;Hebp1
alpha-linolenic (omega3) and linoleic (omega6) acid metabolism	0.014	Abcd1;Elovl5
alpha-linolenic acid (ALA) metabolism	0.014	Abcd1;Elovl5
Dissolution of Fibrin Clot	0.014	S100a10;Anxa2
Nonhomologous End-Joining (NHEJ)	0.016	H2ax;H4c1;H2bc3
Regulation of KIT signaling	0.017	Src;Sos1
RET signaling	0.019	Pdlim7;Src;Sos1
GRB2:SOS provides linkage to MAPK signaling for Integrins	0.019	Src;Sos1
Formation of Senescence-Associated Heterochromatin Foci (SAHF)	0.019	Hmga1;H1-5
Retinoid metabolism and transport	0.020	Apoa2;Apoa4;Apoc2
ABC transporters in lipid homeostasis	0.024	Abcd1;Abcd2
Interleukin-7 signaling	0.024	H3c1;H3c2
Metabolism of fat-soluble vitamins	0.025	Apoa2;Apoa4;Apoc2
PKMTs methylate histone lysines	0.028	H3c1;H4c1;H3c2
Class I peroxisomal membrane protein import	0.029	Abcd1;Abcd2
Recruitment and ATM-mediated phosphorylation of repair and signaling proteins at DNA double strand breaks	0.030	H2ax;H4c1;H2bc3

DNA Double Strand Break Response	0.032	H2ax;H4c1;H2bc3
RHO GTPases activate PAKs	0.032	Myl9;Myl12b
Visual phototransduction	0.035	Apoa2;Fntb;Apoa4;Apoc2
Cellular responses to stress	0.038	H2ax;H3c1;Hist2h2aa1;H4c1;H3-3;Hmga1;H2az2;H1-5;H2bc3;H3c2;Hist1h2af;Nup35
Cellular responses to stimuli	0.040	H2ax;H3c1;Hist2h2aa1;H4c1;H3-3;Hmga1;H2az2;H1-5;H2bc3;H3c2;Hist1h2af;Nup35
Regulation of gap junction activity	0.041	Src
Activated NTRK3 signals through PI3K	0.041	Src
Triglyceride metabolism	0.045	Dgat1;Fabp2
Nucleotide salvage	0.045	Uck1;Tymp
Chromatin organization	0.045	H3c1;Hist2h2aa1;H4c1;H2bc3;H3c2;Hist1h2af
Chromatin modifying enzymes	0.045	H3c1;Hist2h2aa1;H4c1;H2bc3;H3c2;Hist1h2af
Metabolism of nucleotides	0.046	Dnph1;Ak1;Uck1;Tymp

The pathways listed here are altered by the significantly downregulated proteins with the control low-fat diet in C57BL6 mice presented in Supplement Table 11 using an unbiased approach. Reactome (v78) was used to generate the pathway analysis report for species *Mus musculus*. The significance (*p*-value) is calculated by the overrepresentation analysis (hypergeometric distribution).
