# Supplementary material for: Liver Protein Expression in NASH Mice on a High-Fat Diet: Response to Multi-Mineral Intervention
Source: Front Nutr. 2022 May 11;9:859292. doi: 10.3389/fnut.2022.859292 (PMC9130755; doi:10.3389/fnut.2022.859292)
Supplement: Supplementary Table 1 — Mineral Composition of Aquamin® Soluble. [file Data_Sheet_1.zip › SM Table 14 859292.pdf]

Supplement Table 14. Upregulated Proteins by an unbiased proteomic screening with Obeticholic acid (OCA) in high-fat mice

		MS-NASH		C57BL6
**Proteins**	**Genes**	**OCA**	**Aquamin**	**Control**
Nuclear transport factor 2	Nutf2	10.29±20.76	12.12±24.59	8.16±16.12
Fucose mutarotase	Fuom	10.03±19.94	10.28±20.61	8.72±17.40
Glycine cleavage system H protein, mitochondrial	Gcsh	9.05±17.88	7.63±14.93	11.09±21.41
Ras-related protein Rap-1A	Rap1a	5.83±10.73	5.20±9.18	5.66±10.21
IgG receptor FcRn large subunit p51	Fcgrt	5.74±10.39	4.12±6.83	4.17±6.07
Keratin, type II cytoskeletal 79	Krt79	5.73±7.97	26.34±43.11	5.40±4.07
Pigment epithelium-derived factor	Serpinf1	5.58±10.41	6.03±10.85	3.34±5.88
SH3 domain-containing protein 21[#]	Sh3d21	5.43±10.12	8.30±16.34	6.05±10.62
Tubulin beta-5 chain	Tubb5	5.06±9.36	5.81±10.51	4.90±9.25
60S ribosomal protein L36	Rpl36	4.95±8.52	4.32±7.31	4.35±8.31
Transmembrane protein 14C	Tmem14c	4.82±8.80	5.56±10.29	5.97±10.43
Ras-related protein R-Ras	Rras	4.75±8.44	5.46±9.57	6.32±11.74
Tubulin beta-4A chain	Tubb4a	4.73±8.30	4.85±8.51	3.76±6.90
Aquaporin-4	Aqp4	3.99±3.09	0.92±0.56	0.29±0.20
60S acidic ribosomal protein P1	Rplp1	3.61±5.97	3.53±5.59	4.84±8.27
Splicing factor 3B subunit 4	Sf3b4	3.57±5.19	3.03±4.20	3.46±4.27
Galectin-related protein	Lgalsl	3.52±5.77	3.26±5.14	2.86±4.63
Proteasome subunit beta type-3	Psmb3	3.50±5.41	3.56±5.56	3.27±4.30
Endophilin-B1	Sh3glb1	3.47±5.80	3.93±6.42	3.27±5.18
ATP-dependent translocase ABCB1	Abcb1a	3.40±2.67	1.54±0.91	0.91±0.72
Galactose-1-phosphate uridylyltransferase	Galt	3.02±4.19	2.14±2.67	2.84±3.21
U8 snoRNA-decapping enzyme	Nudt16	3.02±4.24	4.03±5.49	3.26±4.15
5'-AMP-activated protein kinase subunit beta-1	Prkab1	3.00±3.74	3.05±4.30	1.92±2.61
Alpha-1-acid glycoprotein 1	Orm1	2.93±5.05	3.98±6.98	6.12±9.25
Glutathione S-transferase A4	Gsta4	2.90±2.09	1.63±1.12	1.95±1.06
Glutaredoxin-1	Glrx	2.85±3.94	2.58±3.23	3.76±5.34
Copper transport protein ATOX1	Atox1	2.84±4.30	3.44±5.40	1.64±2.28
2-hydroxyacyl-CoA lyase 2	Ilvbl	2.82±4.22	2.99±4.38	2.91±4.43
Coatomer subunit epsilon	Cope	2.78±4.10	3.50±5.59	3.27±4.74
Protein PAT1 homolog 1[#]	Patl1	2.74±2.40	4.99±4.23	3.09±2.72
Glutathione S-transferase A2	Gsta2	2.66±0.92*	1.04±0.20	1.27±0.53
All-trans-retinol dehydrogenase [NAD(+)] ADH4	Adh4	2.66±2.14	1.16±0.72	1.77±1.64
Putative RNA-binding protein Luc7-like 1	Luc7l	2.56±3.40	3.20±4.78	2.67±3.04

Histidine ammonia-lyase	Hal	2.54±0.83*	0.71±0.27	1.88±0.49*
S-methylmethionine--homocysteine S-methyltransferase BHMT2	Bhmt2	2.51±3.26	2.70±3.60	2.01±2.67
Peptidyl-prolyl cis-trans isomerase NIMA-interacting 1	Pin1	2.51±2.90	2.52±2.87	2.43±2.54
Cytochrome b-c1 complex subunit 10	Uqcr11	2.45±3.24	2.47±3.24	3.40±4.36
NADH-ubiquinone oxidoreductase chain 5	Mtnd5	2.44±3.18	2.42±3.25	2.55±3.40
Nuclear cap-binding protein subunit 1	Ncbp1	2.41±3.23	2.54±3.24	2.29±2.99
TIP41-like protein	Tiprl	2.36±2.70	2.18±2.36	1.63±1.88
Exportin-5	Xpo5	2.32±2.68	1.77±1.75	1.82±2.36
Aldo-keto reductase family 1 member C18	Akr1c18	2.31±2.65	2.74±3.86	2.52±2.51
NADH dehydrogenase [ubiquinone] 1 alpha subcomplex subunit 11	Ndufa11	2.31±2.93	1.71±1.62	1.55±1.16
Carboxypeptidase B2	Cpb2	2.29±2.74	2.39±2.63	3.39±4.66
Signal recognition particle 19 kDa protein	Srp19	2.29±3.07	2.46±3.39	3.08±4.09
Tubulin beta-2A chain	Tubb2a	2.26±2.77	2.30±2.80	1.00±1.29
Aldose reductase-related protein 2	Akr1b8	2.25±2.73	2.75±3.39	2.84±3.75
STIP1 homology and U box-containing protein 1	Stub1	2.22±2.68	2.07±2.40	2.33±2.80
Small nuclear ribonucleoprotein E	Snrpe	2.22±2.99	2.90±4.12	2.86±3.61
Platelet-activating factor acetylhydrolase IB subunit gamma	Pafah1b3	2.21±1.34	2.45±1.29	1.80±0.90
Acyl-CoA-binding protein	Dbi	2.20±3.02	2.05±2.43	2.05±2.30
Acetyl-coenzyme A transporter 1	Slc33a1	2.20±2.51	2.13±2.62	2.04±2.75
WD repeat-containing protein 18	Wdr18	2.18±2.66	2.03±2.17	2.22±2.64
Thyroid hormone-inducible hepatic protein	Thrsp	2.18±2.04	1.24±0.95	0.70±0.46
COMM domain-containing protein 8	Commd8	2.16±1.99	2.26±2.73	2.63±2.14
Nascent polypeptide-associated complex subunit alpha, muscle-specific form	Naca	2.15±2.63	2.13±2.58	2.29±2.60
Sulfotransferase 1A1	Sult1a1	2.14±1.74	1.35±0.92	1.61±1.90
ATP-binding cassette sub-family D member 4	Abcd4	2.12±1.99	1.50±1.19	4.49±4.10
Glutathione S-transferase theta-2	Gstt2	2.12±1.22	1.31±0.65	0.91±0.45
Neuroplastin	Nptn	2.11±2.67	2.44±3.07	2.33±2.72
Ubiquinol-cytochrome-c reductase complex assembly factor 3	Uqcc3	2.11±1.93	1.82±1.44	1.57±1.50
Histone-lysine N-methyltransferase 2A	Kmt2a	2.08±0.33*	1.14±0.61	1.61±0.32*
SH3-containing GRB2-like protein 3-interacting protein 1	Sgip1	2.08±2.33	1.41±0.96	1.82±2.47
Ig kappa chain V-V region MOPC 149	n/a	2.06±2.70	4.02±4.71	1.52±2.10
Protein quaking	Qki	2.06±2.46	2.49±3.17	1.99±2.52
Small nuclear ribonucleoprotein Sm D1	Snrpd1	2.05±2.04	2.03±2.00	2.13±1.84
AP-2 complex subunit sigma	Ap2s1	2.05±2.57	2.45±3.06	2.61±3.59
[Protein ADP-ribosylarginine] hydrolase	Adprh	2.05±2.29	2.36±2.84	1.53±1.69
4-hydroxy-2-oxoglutarate aldolase, mitochondrial	Hoga1	2.05±1.87	1.27±0.85	3.81±2.81
Ancient ubiquitous protein 1	Aup1	2.04±2.78	2.00±2.73	2.45±3.38

Protein dpy-30 homolog	Dpy30	2.04±2.36	1.74±0.94	1.17±0.28
ADP-ribosylation factor 4	Arf4	2.02±2.64	2.31±2.87	1.84±2.57
Phosphatidylethanolamine N-methyltransferase	Pemt	2.02±2.17	1.81±1.56	2.60±3.30
Transthyretin	Ttr	2.02±2.15	1.57±0.94	0.98±0.41
Cation channel sperm-associated protein subunit beta#	Catsperb	2.00±0.90	1.42±0.54	2.55±1.47
Bile salt export pump	Abcb11	2.00±0.35*	1.03±0.07	1.96±0.57*

These values represent average (± standard deviation) fold-change of abundance ratios for each altered protein compared to the high-fat control group (MS-NASH mice on high-fat) with a cutoff of 2-fold-change in response to OCA intervention. For each upregulated protein with OCA, corresponding values from the other two groups are shown for comparison. The liver samples (from 5 mice in each group) were individually assessed by TMT based differential proteomic expression and data were merged to get averages. (*) represents significance (p-value <0.05) as compared to the high-fat mice. Protein FDR Confidence for all proteins was ≤1% except 3 proteins (#≤2%). These data are also presented in Figure 3A. FDR: False Discovery Rate.
