# Supplementary material for: Liver Protein Expression in NASH Mice on a High-Fat Diet: Response to Multi-Mineral Intervention
Source: Front Nutr. 2022 May 11;9:859292. doi: 10.3389/fnut.2022.859292 (PMC9130755; doi:10.3389/fnut.2022.859292)
Supplement: Supplementary Table 1 — Mineral Composition of Aquamin® Soluble. [file Data_Sheet_1.zip › SM Table 15 859292.pdf]

Supplement Table 15. Downregulated Proteins by an unbiased proteomic screening with Obeticholic acid (OCA) in high-fat mice

		MS-NASH		C57BL6
**Proteins**	**Genes**	**OCA**	**Aquamin**	**Control**
7-alpha-hydroxycholest-4-en-3-one 12-alpha-hydroxylase	Cyp8b1	0.268±0.134*	0.809±0.302	1.012±0.462
Tonsoku-like protein	Tonsl	0.306±0.172*	0.502±0.313	0.342±0.232*
Major urinary protein 2	Mup2	0.440±0.187*	0.586±0.229*	10.396±5.298
Serum amyloid A-1 protein	Saa1	0.447±0.190*	0.728±0.404	3.653±4.613
Major intrinsically disordered NOTCH2-binding receptor 1-like homolog[#]	Minar2	0.451±0.175*	0.491±0.122*	1.062±0.232
Centrosomal protein of 170 kDa	Cep170	0.461±0.341	0.672±0.529	0.260±0.244*
Ornithine aminotransferase, mitochondrial	Oat	0.485±0.124*	0.757±0.355	1.681±0.551
Galectin-1	Lgals1	0.488±0.266*	1.038±0.387	0.461±0.192*

These values represent average (± standard deviation) fold-change of abundance ratios for each altered protein compared to the high-fat control group (MS-NASH mice on high-fat) with a cutoff of 2-fold-change in response to OCA intervention. For each downregulated protein with OCA, corresponding values from the other two groups are shown for comparison. The liver samples (from 5 mice in each group) were individually assessed by TMT based differential proteomic expression and data were merged to get averages. (*) represents significance (p-value <0.05) as compared to the high-fat mice. Protein FDR Confidence for all proteins was ≤1% except 1 protein ([#]≤2%). These data are also presented in Figure 3A. FDR: False Discovery Rate.
