# Supplementary material for: Liver Protein Expression in NASH Mice on a High-Fat Diet: Response to Multi-Mineral Intervention
Source: Front Nutr. 2022 May 11;9:859292. doi: 10.3389/fnut.2022.859292 (PMC9130755; doi:10.3389/fnut.2022.859292)
Supplement: Supplementary Table 1 — Mineral Composition of Aquamin® Soluble. [file Data_Sheet_1.zip › SM Table 16 859292.pdf]

Supplement Table 16. Top pathways associated with upregulated proteins altered with Obeticholic acid (OCA)

Pathway Name	Entities pValue	Mapped entities
mRNA Splicing - Minor Pathway	0.001	Sf3b4;Ncbp1;Snrpe;Snrpd1
SLBP independent Processing of Histone Pre-mRNAs	0.003	Ncbp1;Snrpe
Recycling pathway of L1	0.004	Tubb4a;Tubb2a;P62743
SLBP Dependent Processing of Replication-Dependent Histone Pre-mRNAs	0.004	Ncbp1;Snrpe
Golgi-to-ER retrograde transport	0.005	Pafah1b3;Arf4;Tubb4a;Cope;Tubb2a
Microtubule-dependent trafficking of connexons from Golgi to the plasma membrane	0.007	Tubb4a;Tubb2a
COPI-independent Golgi-to-ER retrograde traffic	0.008	Pafah1b3;Tubb4a;Tubb2a
Transport of connexons to the plasma membrane	0.008	Tubb4a;Tubb2a
COPI-mediated anterograde transport	0.008	Arf4;Tubb4a;Cope;Tubb2a
COPI-dependent Golgi-to-ER retrograde traffic	0.008	Arf4;Tubb4a;Cope;Tubb2a
L1CAM interactions	0.020	Tubb4a;Tubb2a;P62743
Intra-Golgi and retrograde Golgi-to-ER traffic	0.020	Pafah1b3;Arf4;Tubb4a;Cope;Tubb2a
Carboxyterminal post-translational modifications of tubulin	0.020	Tubb4a;Tubb2a
Sealing of the nuclear envelope (NE) by ESCRT-III	0.020	Tubb4a;Tubb2a
The role of GTSE1 in G2/M progression after G2 checkpoint	0.023	Tubb4a;Psmb3;Tubb2a
Processing of Capped Intronless Pre-mRNA	0.023	Ncbp1;Snrpe
Glyoxylate metabolism and glycine degradation	0.027	Hoga1;Gcsh
Gap junction assembly	0.028	Tubb4a;Tubb2a
Glycine degradation	0.033	Gcsh
Ion influx/efflux at host-pathogen interface	0.033	Atox1
Aggrephagy	0.035	Tubb4a;Tubb2a
ER to Golgi Anterograde Transport	0.035	Arf4;Tubb4a;Cope;Tubb2a
Recruitment of NuMA to mitotic centrosomes	0.039	Tubb5;Tubb4a;Tubb2a
SRP-dependent cotranslational protein targeting to membrane	0.040	Srp19;Rplp1;Rpl36
ARMS-mediated activation	0.041	Rap1a
Galactose catabolism	0.041	Galt
Nonsense Mediated Decay (NMD) independent of the Exon Junction Complex (EJC)	0.043	Ncbp1;Rplp1;Rpl36
Metabolism of RNA	0.046	Sf3b4;Ncbp1;Snrpe;Psmb3;Rplp1;Patl1;Rpl36;Snrpd1;Wdr18
Gap junction trafficking	0.047	Tubb4a;Tubb2a
GDP-fucose biosynthesis	0.049	Fuom

The pathways listed here are altered by the significantly upregulated proteins with the intervention “OCA” presented in Supplement Table 14 using an unbiased approach. Reactome (v78) was used to generate the pathway analysis report for species *Mus musculus*. The significance (*p*-value) is calculated by the overrepresentation analysis (hypergeometric distribution).
