# Supplementary material for: Liver Protein Expression in NASH Mice on a High-Fat Diet: Response to Multi-Mineral Intervention
Source: Front Nutr. 2022 May 11;9:859292. doi: 10.3389/fnut.2022.859292 (PMC9130755; doi:10.3389/fnut.2022.859292)
Supplement: Supplementary Table 1 — Mineral Composition of Aquamin® Soluble. [file Data_Sheet_1.zip › SM Table 17 859292.pdf]

Supplement Table 17. Top pathways associated with downregulated proteins altered with Obeticholic acid (OCA)

Pathway name	Entities pValue	Mapped entities
Sterols are 12-hydroxylated by CYP8B1	0.002	Cyp8b1
Synthesis of Prostaglandins (PG) and Thromboxanes (TX)	0.011	Cyp8b1
Synthesis of bile acids and bile salts via 24-hydroxycholesterol	0.011	Cyp8b1
Glutamate and glutamine metabolism	0.011	Oat
Synthesis of bile acids and bile salts via 27-hydroxycholesterol	0.011	Cyp8b1
Eicosanoids	0.015	Cyp8b1
Nicotinamide salvaging	0.017	Cyp8b1
Synthesis of bile acids and bile salts via 7alpha-hydroxycholesterol	0.019	Cyp8b1
Endogenous sterols	0.024	Cyp8b1
Nicotinate metabolism	0.027	Cyp8b1
Synthesis of bile acids and bile salts	0.028	Cyp8b1
Bile acid and bile salt metabolism	0.037	Cyp8b1
Arachidonic acid metabolism	0.049	Cyp8b1

The pathways listed here are altered by the downregulated proteins with the intervention “Obeticholic acid (OCA)” presented in Supplement Table 15 using an unbiased approach. Reactome (v78) was used to generate the pathway analysis report for species *Mus musculus*. The significance (*p* value) is calculated by the overrepresentation analysis (hypergeometric distribution).
