# Supplementary material for: Liver Protein Expression in NASH Mice on a High-Fat Diet: Response to Multi-Mineral Intervention
Source: Front Nutr. 2022 May 11;9:859292. doi: 10.3389/fnut.2022.859292 (PMC9130755; doi:10.3389/fnut.2022.859292)
Supplement: Supplementary Table 1 — Mineral Composition of Aquamin® Soluble. [file Data_Sheet_1.zip › SM Table 18 859292.pdf]

**Supplement Table 18. Top pathways associated with upregulated (& common) proteins altered with Aquamin and Obeticholic acid (OCA)**

| Pathway Name | Entities pValue | Mapped entities |
|---|---|---|
| mRNA Splicing - Minor Pathway | 0.0003 | Sf3b4;Ncbp1; Snrpe;Snrpd1 |
| *Golgi-to-ER retrograde transport | 0.001 | Pafah1b3;Arf4; Tubb4a; Cope;Tubb2a |
| *Recycling pathway of L1 | 0.001 | Tubb4a;Tubb2a; Ap2s1 |
| SLBP independent Processing of Histone Pre-mRNAs | 0.002 | Ncbp1; Snrpe |
| SLBP Dependent Processing of Replication-Dependent Histone Pre-mRNAs | 0.002 | Ncbp1; Snrpe |
| *COPI-dependent Golgi-to-ER retrograde traffic | 0.003 | Arf4; Tubb4a; Cope;Tubb2a |
| *COPI-mediated anterograde transport | 0.003 | Arf4; Tubb4a; Cope;Tubb2a |
| *COPI-independent Golgi-to-ER retrograde traffic | 0.003 | Pafah1b3; Tubb4a;Tubb2a |
| *Microtubule-dependent trafficking of connexons from Golgi to the plasma membrane | 0.004 | Tubb4a;Tubb2a |
| *Transport of connexons to the plasma membrane | 0.004 | Tubb4a;Tubb2a |
| *Intra-Golgi and retrograde Golgi-to-ER traffic | 0.005 | Pafah1b3;Arf4; Tubb4a; Cope;Tubb2a |
| Metabolism of RNA | 0.007 | Sf3b4;Ncbp1; Snrpe;Q9R1P1;Rplp1;Patl1;Rpl36;Snrpd1;Wdr18 |
| L1CAM interactions | 0.008 | Tubb4a;Tubb2a; Ap2s1 |
| The role of GTSE1 in G2/M progression after G2 checkpoint | 0.01 | Tubb4a;Q9R1P1;Tubb2a |
| *Carboxyterminal post-translational modifications of tubulin | 0.01 | Tubb4a;Tubb2a |
| *Sealing of the nuclear envelope (NE) by ESCRT-III | 0.01 | Tubb4a;Tubb2a |
| *ER to Golgi Anterograde Transport | 0.01 | Arf4; Tubb4a; Cope;Tubb2a |
| Processing of Capped Intronless Pre-mRNA | 0.01 | Ncbp1; Snrpe |
| *Membrane Trafficking | 0.01 | Pafah1b3; Arf4;Tubb2a |
| *Gap junction assembly | 0.02 | Tubb4a;Tubb2a |
| Recruitment of NuMA to mitotic centrosomes | 0.02 | Tubb5; Tubb4a;Tubb2a |
| SRP-dependent cotranslational protein targeting to membrane | 0.02 | Srp19;Rplp1;Rpl36 |
| Nonsense Mediated Decay independent of the Exon Junction Complex | 0.02 | Ncbp1;Rplp1;Rpl36 |
| *Aggrephagy | 0.02 | Tubb4a;Tubb2a |
| *VxPx cargo-targeting to cilium | 0.02 | Arf4 |
| *Asparagine N-linked glycosylation | 0.02 | Arf4; Tubb4a; Cope;Tubb2a;Fuom |
| *Transport to the Golgi and subsequent modification | 0.02 | Arf4; Tubb4a; Cope;Tubb2a |
| G2/M Transition | 0.02 | Tubb5; Tubb4a;Q9R1P1;Tubb2a |
| mRNA Splicing - Major Pathway | 0.02 | Sf3b4;Ncbp1; Snrpe;Snrpd1 |
| *Ion influx/efflux at host-pathogen interface | 0.02 | Atox1 |
| Glycine degradation | 0.02 | Gcsh |

| | | |
|---|---|---|
| Mitotic G2-G2/M phases | 0.02 | Tubb5; Tubb4a;Q9R1P1;Tubb2a |
| *Gap junction trafficking | 0.03 | Tubb4a;Tubb2a |
| *Vesicle-mediated transport | 0.03 | Pafah1b3; Arf4;Tubb2a |
| mRNA Splicing | 0.03 | Sf3b4;Ncbp1; Snrpe;Snrpd1 |
| Hedgehog 'off' state | 0.03 | Tubb4a;Q9R1P1;Tubb2a |
| *Energy dependent regulation of mTOR by LKB1-AMPK | 0.03 | Prkab1 |
| *Gap junction trafficking and regulation | 0.03 | Tubb4a;Tubb2a |
| ARMS-mediated activation | 0.03 | Rap1a |
| Galactose catabolism | 0.03 | Galt |
| *Cilium Assembly | 0.03 | Tubb5;Arf4; Tubb4a;Tubb2a |
| Nonsense Mediated Decay enhanced by the Exon Junction Complex | 0.03 | Ncbp1;Rplp1;Rpl36 |
| Nonsense-Mediated Decay (NMD) | 0.03 | Ncbp1;Rplp1;Rpl36 |
| MHC class II antigen presentation | 0.03 | Tubb4a;Tubb2a; Ap2s1 |
| GDP-fucose biosynthesis | 0.04 | Fuom |
| *Autophagy | 0.04 | Tubb4a;Prkab1;Tubb2a |
| *Macroautophagy | 0.04 | Tubb4a;Prkab1;Tubb2a |
| Metabolism of non-coding RNA | 0.04 | Snrpe;Snrpd1 |
| snRNP Assembly | 0.04 | Snrpe;Snrpd1 |
| Regulation of RUNX2 expression and activity | 0.04 | Stub1;Q9R1P1 |
| *MTOR signalling | 0.04 | Prkab1 |
| HSP90 chaperone cycle for steroid hormone receptors in the presence of ligand | 0.04 | Tubb4a;Tubb2a |
| Intraflagellar transport | 0.04 | Tubb4a;Tubb2a |
| Phosphate bond hydrolysis by NUDT proteins | 0.04 | Nudt16 |
| Kinesins | 0.04 | Tubb4a;Tubb2a |
| *Organelle biogenesis and maintenance | 0.05 | Tubb5;Arf4; Tubb4a;Tubb2a |
| Estrogen biosynthesis | 0.05 | Akr1b8 |
| Sema4D mediated inhibition of cell attachment and migration | 0.05 | Rras |
| *Cargo trafficking to the periciliary membrane | 0.05 | Arf4 |

The pathways listed here are altered by the upregulated proteins (at 2-fold change threshold) common among the interventions “Aquamin”, “OCA” and low-fat mice presented in Supplement Table 2 using an unbiased approach. 49 proteins were common among three interventions and 9 were common between “OCA” & “Aquamin.” (*) represents pathways unique to Aquamin and OCA interventions only. Reactome (v78) was used to generate the pathway analysis report for species *Mus musculus*. The significance (*p*-value) is calculated by the overrepresentation analysis (hypergeometric distribution).
