# Supplementary material for: Liver Protein Expression in NASH Mice on a High-Fat Diet: Response to Multi-Mineral Intervention
Source: Front Nutr. 2022 May 11;9:859292. doi: 10.3389/fnut.2022.859292 (PMC9130755; doi:10.3389/fnut.2022.859292)
Supplement: Supplementary Table 1 — Mineral Composition of Aquamin® Soluble. [file Data_Sheet_1.zip › SM Table 2 859292.pdf]

Supplement Table 2. Common and unique upregulated proteins (at 2-fold change threshold)

Accession	Protein names	Gene Name
	**Common Proteins among Aquamin, OCA and C57BL6 (low-fat): 46**	
Q7TSG5	SH3 domain-containing protein 21	Sh3d21
P62743	AP-2 complex subunit sigma	Ap2s1
Q8R2K1	Fucose mutarotase	Fuom
Q60590	Alpha-1-acid glycoprotein 1	Agp1
P99024	Tubulin beta-5 chain	Tubb5
Q9CQN6	Transmembrane protein 14C	Tmem14c
P47955	60S acidic ribosomal protein P1	Rplp1
P03921	NADH-ubiquinone oxidoreductase chain 5	Mtnd5
P47964	60S ribosomal protein L36	Rpl36
Q99J27	Acetyl-coenzyme A transporter 1	Acatn
Q9JHH6	Carboxypeptidase B2	Cpb2
P10833	Ras-related protein R-Ras	Rras
Q9R1P1	Proteasome subunit beta type-3	Psmb3
Q9CPX8	Cytochrome b-c1 complex subunit 10	Uqcr11
Q61559	IgG receptor FcRn large subunit p51	Fcgrt
P70295	Lipid droplet-regulating VLDL assembly factor AUP1	Aup1
Q03249	Galactose-1-phosphate uridylyltransferase	Galt
P97300	Neuroplastin	Nptn
Q8K023	Aldo-keto reductase family 1 member C18	Akr1c18
P31786	Acyl-CoA-binding protein	Dbi
Q91WS4	S-methylmethionine--homocysteine S-methyltransferase BHMT2	Bhmt2
P97298	Pigment epithelium-derived factor	Serpinf1
Q9JK48	Endophilin-B1	Sh3glb1
Q9CYI4	Putative RNA-binding protein Luc7-like 1	Luc7l
Q9CZG3	COMM domain-containing protein 8	Commd8
Q8VED9	Galectin-related protein	Lgalsl
Q6P3D0	U8 snoRNA-decapping enzyme (m7GpppN-mRNA hydrolase)	Nudt16
Q9D7A6	Signal recognition particle 19 kDa protein	Srp19
O89079	Coatomer subunit epsilon	Cope
P62835	Ras-related protein Rap-1A	Rap1a
Q3TC46	Protein PAT1 homolog 1	Patl1
Q9D6F9	Tubulin beta-4A chain	Tubb4a
Q9QUH0	Glutaredoxin-1	Glrx
P45377	Aldose reductase-related protein 2	Akr1b8
Q3UYV9	Nuclear cap-binding protein subunit 1	Ncbp1
P61971	Nuclear transport factor 2	Nutf2
P62305	Small nuclear ribonucleoprotein E	Snrpe
Q4VBE8	WD repeat-containing protein 18	Wdr18
Q8BU33	2-hydroxyacyl-CoA lyase 2	Ilvbl
P70670	Alpha-NAC, muscle-specific form	Naca
Q9QUR7	Peptidyl-prolyl cis-trans isomerase NIMA-interacting 1	Pin1

Q91WK5	Glycine cleavage system H protein, mitochondrial	Gcsh
Q9WUD1	E3 ubiquitin-protein ligase CHIP	Stub1
Q8QZY9	Splicing factor 3B subunit 4	Sf3b4
Q8VED5	Keratin, type II cytoskeletal 79	Krt79
P62315	Small nuclear ribonucleoprotein Sm D1	Snrpd1
	**Common Proteins between OCA and C57BL6 (low-fat): 4**	
Q61907	Phosphatidylethanolamine N-methyltransferase	Pemt
A2RTF1	Cation channel sperm-associated protein subunit beta	Catsperb
O89016	Lysosomal cobalamin transporter ABCD4	Abcd4
Q9DCU9	4-hydroxy-2-oxoglutarate aldolase, mitochondrial	Hoga1
	**Common Proteins between Aquamin and C57BL6 (low-fat): 12**	
Q6IFX2	Keratin, type I cytoskeletal 42	Krt42
Q3TTY5	Keratin, type II cytoskeletal 2 epidermal	Krt2
Q9EPB5	Serine hydrolase-like protein	Serhl
Q922U2	Keratin, type II cytoskeletal 5	Krt5
Q9QWL7	Keratin, type I cytoskeletal 17	Krt17
P35762	CD81 antigen	Cd81
Q99KE1	NAD-dependent malic enzyme, mitochondrial	Me2
Q61781	Keratin, type I cytoskeletal 14	Krt14
Q3UV17	Keratin, type II cytoskeletal 2 oral	Krt76
P04104	Keratin, type II cytoskeletal 1	Krt1
P02535	Keratin, type I cytoskeletal 10	Krt10
Q9Z2K1	Keratin, type I cytoskeletal 16	Krt16
	**Common Proteins between Aquamin and OCA: 9**	
Q61205	Platelet-activating factor acetylhydrolase IB subunit alpha1	Pafah1b3
O08997	Copper transport protein ATOX1	Atox1
Q7TMM9	Tubulin beta-2A chain	Tubb2a
Q9R078	5'-AMP-activated protein kinase subunit beta-1	Prkab1
P54923	[Protein ADP-ribosylarginine] hydrolase	Adprh
P61750	ADP-ribosylation factor 4	Arf4
P01636	Ig kappa chain V-V region MOPC 149	n/a
Q8BH58	TIP41-like protein	Tiprl
Q9QYS9	Protein quaking	Qki
	**Unique Proteins to Aquamin: 19**	
P14434	H-2 class II histocompatibility antigen, A-B alpha chain	H2-Aa
Q8CHW4	Translation initiation factor eIF-2B subunit epsilon	Eif2b5
Q99JW4	LIM and senescent cell antigen-like-containing domain protein 1	Lims1
P01864	Ig gamma-2A chain C region secreted form	n/a
P04441	H-2 class II histocompatibility antigen gamma chain	Cd74
P50428	Arylsulfatase A	Arsa
Q61093	Cytochrome b-245 heavy chain	Cybb
Q9DBC7	cAMP-dependent protein kinase type I-alpha regulatory subunit	Prkar1a
Q8K1B8	Fermitin family homolog 3	Fermt3
P42225	Signal transducer and activator of transcription 1	Stat1
P18468	H-2 class II histocompatibility antigen, I-A beta chain	H2-Eb1

A2AWP8	Rho guanine nucleotide exchange factor 10-like protein	Arhgef10l
Q01514	Guanylate-binding protein 1	Gbp1
P50543	Protein S100-A11	S100a11
P28063	Proteasome subunit beta type-8	Psmb8
P24638	Lysosomal acid phosphatase	Acp2
Q99LU0	Charged multivesicular body protein 1b-1	Chmp1b1
Q9Z0M5	Lysosomal acid lipase/cholesteryl ester hydrolase	Lipa
Q9QZ25	Vascular non-inflammatory molecule 3	Vnn3
	**Unique Proteins to OCA: 17**	
P55200	Histone-lysine N-methyltransferase 2A	Kmt2a
P10648	Glutathione S-transferase A2	Gsta2
Q8VD37	SH3-containing GRB2-like protein 3-interacting protein 1	Sgip1
P35492	Histidine ammonia-lyase	Hal
P52840	Sulfotransferase 1A1	Sult1a1
Q9QYY9	All-trans-retinol dehydrogenase [NAD(+)] ADH4	Adh4
Q8K2T4	Ubiquinol-cytochrome-c reductase complex assembly factor 3	Uqcc3
P21447	ATP-dependent translocase ABCB1	Abcb1a
Q61133	Glutathione S-transferase theta-2	Gstt2
P55088	Aquaporin-4	Aqp4
Q924C1	Exportin-5	Xpo5
Q9QY30	Bile salt export pump	Abcb11
Q9D8B4	NADH dehydrogenase [ubiquinone] 1 alpha subcomplex subunit 11	Ndufa11
P24472	Glutathione S-transferase A4	Gsta4
P07309	Transthyretin	Ttr
Q99LT0	Protein dpy-30 homolog	Dpy30
Q62264	Thyroid hormone-inducible hepatic protein	Thrsp
	**Unique Proteins to C57BL6 (Low-fat): 120**	
P01869	Ig gamma-1 chain C region, membrane-bound form	Ighg1
Q8VCN5	Cystathionine gamma-lyase	Cth
Q80W21	Glutathione S-transferase Mu 7	Gstm7
Q64374	Regucalcin	Rgn
P00158	Cytochrome b	Mt-Cyb
Q63836	Selenium-binding protein 2	Selenbp2
O35423	Serine--pyruvate aminotransferase, mitochondrial	Agxt
Q91WS0	CDGSH iron-sulfur domain-containing protein 1	Cisd1
Q9DCP2	Sodium-coupled neutral amino acid transporter 3	Slc38a3
Q8BTY1	Kynurenine--oxoglutarate transaminase 1	Kyat1
P19157	Glutathione S-transferase P 1	Gstp1
Q9WVM8	Kynurenine/alpha-aminoadipate aminotransferase, mitochondrial	Aadat
P56654	Cytochrome P450 2C37	Cyp2c37
Q91X77	Cytochrome P450 2C50	Cyp2c50
Q9QXZ6	Solute carrier organic anion transporter family member 1A1	Slco1a1
Q91W64	Cytochrome P450 2C70	Cyp2c70
Q9DBW0	Cytochrome P450 4V2	Cyp4v2
Q8VE09	Tetratricopeptide repeat protein 39C	Ttc39c

Q91ZI0	Cadherin EGF LAG seven-pass G-type receptor 3	Celsr3
Q9JHI5	Isovaleryl-CoA dehydrogenase, mitochondrial	Ivd
Q9D906	Ubiquitin-like modifier-activating enzyme ATG7	Atg7
Q8CIF4	Biotinidase	Btd
Q8BLN5	Lanosterol synthase	Lss
Q8BH35	Complement component C8 beta chain	C8b
P61922	4-aminobutyrate aminotransferase, mitochondrial	Abat
Q283N4	2-oxo-4-hydroxy-4-carboxy-5-ureidoimidazoline decarboxylase	Urad
E9Q5K4	Cytochrome P450 2C44	Cyp2c23
Q9DCT1	1,5-anhydro-D-fructose reductase	Akr1e2
Q5SGK3	Aldehyde oxidase 2	Aox2
Q6ZQK0	Condensin-2 complex subunit D3	Ncapd3
Q05816	Fatty acid-binding protein 5	Fabp5
Q8C0L9	Glycerophosphocholine phosphodiesterase GPCPD1	Gpcpd1
Q5FW60	Major urinary protein 20	Mup20
Q91VA0	Acyl-coenzyme A synthetase ACSM1, mitochondrial	Acsm1
Q60991	Cytochrome P450 7B1	Cyp7b1
Q9D0J8	Parathymosin	Ptms
Q63880	Carboxylesterase 3A	Ces3a
O54782	Epididymis-specific alpha-mannosidase	Man2b2
Q8BZB2	Phosphopantothenoylcysteine decarboxylase	Ppcdc
Q8QZR1	Tyrosine aminotransferase	Tat
Q9JHU9	Inositol-3-phosphate synthase 1	Isyna1
P01887	Beta-2-microglobulin	B2m
Q8VCW8	Medium-chain acyl-CoA ligase ACSF2, mitochondrial	Acsf2
P56135	ATP synthase subunit f, mitochondrial	Atp5mf
P42703	Leukemia inhibitory factor receptor	Lifr
Q80XI6	Mitogen-activated protein kinase kinase kinase 11	Map3k11
Q01768	Nucleoside diphosphate kinase B	Nme2
Q920A5	Retinoid-inducible serine carboxypeptidase	Scpep1
Q8QZR3	Pyrethroid hydrolase Ces2a	Ces2a
Q920E5	Farnesyl pyrophosphate synthase	Fdps
Q9WU19	Hydroxyacid oxidase 1	Hao1
P00186	Cytochrome P450 1A2	Cyp1a2
Q99PG0	Arylacetamide deacetylase	Aadac
Q9WUR9	Adenylate kinase 4, mitochondrial	Ak4
A6BLY7	Keratin, type I cytoskeletal 28	Krt28
Q8VC97	Beta-ureidopropionase	Upb1
O35943	Frataxin, mitochondrial	Fxn
P28666	Murinoglobulin-2	Mug2
P50429	Arylsulfatase B	Arsb
Q8JZZ0	UDP-glucuronosyltransferase 3A2	Ugt3a2
P58044	Isopentenyl-diphosphate Delta-isomerase 1	Idi1
Q9DCY0	Glycine N-acyltransferase-like protein Keg1	Keg1
Q9ERY9	Ergosterol biosynthetic protein 28 homolog	Erg28

Q6XVG2	Cytochrome P450 2C54	Cyp2c54
O08600	Endonuclease G, mitochondrial	Endog
P11589	Major urinary protein 2	Mup2
Q9CZP5	Mitochondrial chaperone BCS1	Bcs1l
P07759	Serine protease inhibitor A3K	Serpina3k
Q9R1J0	Sterol-4-alpha-carboxylate 3-dehydrogenase, decarboxylating	Nsdhl
Q62452	UDP-glucuronosyltransferase 1A9	Ugt1a9
Q8VCG4	Complement component C8 gamma chain	C8g
Q8K0C4	Lanosterol 14-alpha demethylase	Cyp51a1
Q8K0L9	Zinc finger and BTB domain-containing protein 20	Zbtb20
G3X982	Aldehyde oxidase 3	Aox3
P01878	Ig alpha chain C region	n/a
Q571F8	Glutaminase liver isoform, mitochondrial	Gls2
Q9D8B6	Protein FAM210B, mitochondrial	Fam210b
Q9CRA4	Methylsterol monooxygenase 1	Msmo1
Q61694	NADPH-dependent 3-keto-steroid reductase Hsd3b5	Hsd3b5
Q91WN4	Kynurenine 3-monooxygenase	Kmo
Q61176	Arginase-1	Arg1
Q9R008	Mevalonate kinase	Mvk
P33267	Cytochrome P450 2F2	Cyp2f2
Q71KT5	Delta(14)-sterol reductase TM7SF2	Tm7sf2
Q8K2I4	Beta-mannosidase	Manba
Q9DBE0	Cysteine sulfinic acid decarboxylase	Csad
Q9Z2V4	Phosphoenolpyruvate carboxykinase, cytosolic [GTP]	Pck1
P17439	Lysosomal acid glucosylceramidase	Gba
Q9DB29	Isoamyl acetate-hydrolyzing esterase 1 homolog	Iah1
Q9Z1R3	Apolipoprotein M	Apom
Q6IFZ6	Keratin, type II cytoskeletal 1b	Krt77
P49935	Pro-cathepsin H	Ctsh
P43024	Cytochrome c oxidase subunit 6A1, mitochondrial	Cox6a1
Q9R013	Cathepsin F	Ctsf
Q91WG0	Acylcarnitine hydrolase	Ces2c
Q9QWR8	Alpha-N-acetylgalactosaminidase	Naga
Q9QXF8	Glycine N-methyltransferase	Gnmt
P11725	Ornithine transcarbamylase, mitochondrial	Otc
O88668	Protein CREG1	Creg1
Q8C196	Carbamoyl-phosphate synthase [ammonia], mitochondrial	Cps1
Q63886	UDP-glucuronosyltransferase 1A1	Ugt1a1
Q99K67	Alpha-aminoadipic semialdehyde synthase, mitochondrial	Aass
Q01279	Epidermal growth factor receptor	Egfr
Q99P30	Peroxisomal coenzyme A diphosphatase NUDT7	Nudt7
P52843	Sulfotransferase 2A1	Sult2a1
Q8VCU1	Carboxylesterase 3B	Ces3b
Q80W22	Threonine synthase-like 2	Thnsl2
P05366	Serum amyloid A-1 protein	Saa1

Q9D0S9	Histidine triad nucleotide-binding protein 2, mitochondrial	Hint2
P00688	Pancreatic alpha-amylase	Amy2
Q9JLF6	Protein-glutamine gamma-glutamyltransferase K	Tgm1
Q99LB7	Sarcosine dehydrogenase, mitochondrial	Sardh
Q9EP72	ER membrane protein complex subunit 7	Emc7
Q9DCG2	CD302 antigen	Cd302
Q00898	Alpha-1-antitrypsin 1-5	Serpina1e
P16331	Phenylalanine-4-hydroxylase	Pah
P52825	Carnitine O-palmitoyltransferase 2, mitochondrial	Cpt2
Q9D273	Corrinoid adenosyltransferase	Mmab
P16460	Argininosuccinate synthase	Ass1
Q91XF0	Pyridoxine-5'-phosphate oxidase	Pnpo

The liver samples (from 5 mice in each group) were individually assessed by TMT-based differential proteomic expression and data were merged to get averages. Protein FDR Confidence for all proteins was $\leq$2%. FDR: False Discovery Rate. These altered proteins were upregulated compared to the high-fat control group (MS-NASH mice on a high-fat diet) with a 2-fold-change threshold. These data (Venn diagrams) are shown in Figure 3A.
