# Supplementary material for: Liver Protein Expression in NASH Mice on a High-Fat Diet: Response to Multi-Mineral Intervention
Source: Front Nutr. 2022 May 11;9:859292. doi: 10.3389/fnut.2022.859292 (PMC9130755; doi:10.3389/fnut.2022.859292)
Supplement: Supplementary Table 1 — Mineral Composition of Aquamin® Soluble. [file Data_Sheet_1.zip › SM Table 3 859292.pdf]

Supplement Table 3. Common and unique downregulated proteins (at 2-fold change threshold)

Accession	Protein names	Gene Name
	**Common Proteins among Aquamin, OCA and C57BL6 (low-fat): 1**	
Q6NZL6	Tonsoku-like protein	Tonsl
	**Common Proteins between OCA and C57BL6 (low-fat): 2**	
Q6A065	Centrosomal protein of 170 kDa	Cep170
P16045	Galectin-1	Lgals1
	**Common Proteins between Aquamin and C57BL6 (low-fat): 1**	
Q921W4	Quinone oxidoreductase-like protein 1	Cryzl1
	**Common Proteins between Aquamin and OCA: 1**	
Q8C4X7	Membrane integral NOTCH2-associated receptor 2	Minar2
	**Unique Proteins to Aquamin: 2**	
Q6XVG2	Cytochrome P450 2C54	Cyp2c54
Q00898	Alpha-1-antitrypsin 1-5	Serpina1e
	**Unique Proteins to OCA: 4**	
P29758	Ornithine aminotransferase, mitochondrial	Oat
P11589	Major urinary protein 2	Mup2
O88962	7-alpha-hydroxycholest-4-en-3-one 12-alpha-hydroxylase	Cyp8b1
P05366	Serum amyloid A-1 protein	Saa1
	**Unique Proteins to C57BL6 (Low-fat): 122**	
P62806	Histone H4	H4c1
A2A995	FYN-binding protein 2	Fyb2
Q9R0Y5	Adenylate kinase isoenzyme 1	Ak1
Q8K2K6	Arf-GAP domain and FG repeat-containing protein 1	Agfg1
Q62245	Son of sevenless homolog 1	Sos1
Q3U4I7	Pyridine nucleotide-disulfide oxidoreductase domain-containing protein 2	Pyroxd2
Q8CC35	Synaptopodin	Synpo
Q8K4G5	Actin-binding LIM protein 1	Ablim1
P97819	85/88 kDa calcium-independent phospholipase A2	Pla2g6
Q8VCR2	17-beta-hydroxysteroid dehydrogenase 13	Hsd17b13
Q61285	ATP-binding cassette sub-family D member 2	Abcd2
Q2NL51	Glycogen synthase kinase-3 alpha	Gsk3a
Q6NZF1	Zinc finger CCCH domain-containing protein 11A	Zc3h11a
Q64475	Histone H2B type 1-B	H2bc3
Q9DBS9	Oxysterol-binding protein-related protein 3	Osbpl3
Q8BK63	Casein kinase I isoform alpha	Csnk1a1
Q810Z1	Epididymal-specific lipocalin-10	Lcn10
P16110	Galectin-3	Lgals3
Q62417	Sorbin and SH3 domain-containing protein 1	Sorbs1
Q8R4R6	Nucleoporin NUP35	Nup35
Q8VC49	Interferon alpha-inducible protein 27-like protein 2B	Ifi27l2b
Q69Z37	Sterile alpha motif domain-containing protein 9-like	Samd9l
Q8BJF9	Charged multivesicular body protein 2b	Chmp2b
P12790	Cytochrome P450 2B9	Cyp2b9

Q8K2F0	Bromodomain-containing protein 3	Brd3
Q60875	Rho guanine nucleotide exchange factor 2	Arhgef2
Q8CGP5	Histone H2A type 1-F	Hist1h2af
Q91V92	ATP-citrate synthase	Acly
Q8CDN9	Leucine-rich repeat-containing protein 9	Lrrc9
O89053	Coronin-1A	Coro1a
P19096	Fatty acid synthase	Fasn
Q99LJ0	CTTNBP2 N-terminal-like protein	Cttnbp2nl
Q14DH7	Acyl-CoA synthetase short-chain family member 3, mitochondrial	Acss3
Q8CIB6	Transmembrane protein 230	Tmem230
Q9R257	Heme-binding protein 1	Hebp1
Q9DBM2	Peroxisomal bifunctional enzyme	Ehhadh
Q9Z2H5	Band 4.1-like protein 1	Epb41l1
Q9R1Q7	Proteolipid protein 2	Plp2
Q3TJD7	PDZ and LIM domain protein 7	Pdlim7
Q9D0P0	Emopamil-binding protein-like	Ebpl
Q9Z2A7	Diacylglycerol O-acyltransferase 1	Dgat1
P04441	H-2 class II histocompatibility antigen gamma chain	Cd74
Q9CQ19	Myosin regulatory light polypeptide 9	Myl9
P55050	Fatty acid-binding protein, intestinal	Fabp2
Q8R1N4	NudC domain-containing protein 3	Nudcd3
O88492	Perilipin-4	Plin4
Q99MQ5	Collagen alpha-1(XXV) chain	Col25a1
P56656	Cytochrome P450 2C39	Cyp2c39
Q9D939	Sulfotransferase 1C2	Sult1c2
Q91YR9	Prostaglandin reductase 1	Ptgr1
Q9QZC8	Protein ABHD1	Abhd1
Q9D312	Keratin, type I cytoskeletal 20	Krt20
O54931	A-kinase anchor protein 2	Akap2
P84244	Histone H3.3	H3-3a
Q6GSS7	Histone H2A type 2-A	Hist2h2aa1
Q99N42	Thymidine phosphorylase	Tymp
P97315	Cysteine and glycine-rich protein 1	Csrp1
Q9D964	Glycine amidinotransferase, mitochondrial	Gatm
Q6Y7W8	GRB10-interacting GYF protein 2	Gigyf2
P06728	Apolipoprotein A-IV	Apoa4
P35576	Glucose-6-phosphatase catalytic subunit 1	G6pc1
Q5SWU9	Acetyl-CoA carboxylase 1	Acaca
P05480	Neuronal proto-oncogene tyrosine-protein kinase Src	Src
Q9QYR9	Acyl-coenzyme A thioesterase 2, mitochondrial	Acot2
Q6PCN7	Helicase-like transcription factor	Hltf
Q61753	D-3-phosphoglycerate dehydrogenase	Phgdh
P13516	Acyl-CoA desaturase 1	Scd1
P17095	High mobility group protein HMG-I/HMG-Y	Hmga1
P55088	Aquaporin-4	Aqp4

Q6ZPJ0	Testis-expressed protein 2	Tex2
Q3TFD2	Lysophosphatidylcholine acyltransferase 1	Lpcat1
Q9JJY3	Sphingomyelin phosphodiesterase 3	Smpd3
Q62523	Zyxin	Zyx
Q9Z211	Peroxisomal membrane protein 11A	Pex11a
P27661	Histone H2AX	H2ax
Q80VJ3	2'-deoxynucleoside 5'-phosphate N-hydrolase 1	Dnph1
Q8K2I1	Protein farnesyltransferase subunit beta	Fntb
Q3UHK8	Trinucleotide repeat-containing gene 6A protein	Tnrc6a
Q9Z1W9	STE20/SPS1-related proline-alanine-rich protein kinase	Stk39
Q3THW5	Histone H2A.V	H2az2
Q80X19	Collagen alpha-1(XIV) chain	Col14a1
P19973	Lymphocyte-specific protein 1	Lsp1
P70429	Ena/VASP-like protein	Evl
Q99P72	Reticulon-4	Rtn4
Q9Z0G0	PDZ domain-containing protein GIPC1	Gipc1
O35855	Branched-chain-amino-acid aminotransferase, mitochondrial	Bcat2
P84228	Histone H3.2	H3c2
Q9QYH6	Melanoma-associated antigen D1	Maged1
P52623	Uridine-cytidine kinase 1	Uck1
P27546	Microtubule-associated protein 4	Map4
P48410	ATP-binding cassette sub-family D member 1	Abcd1
Q91XC8	Death-associated protein 1	Dap
P43276	Histone H1.5	H1-5
P0C7L0	WAS/WASL-interacting protein family member 3	Wipf3
O35678	Monoglyceride lipase	Mgll
P08207	Protein S100-A10	S100a10
Q8BHI7	Elongation of very long chain fatty acids protein 5	Elovl5
Q3THE2	Myosin regulatory light chain 12B	Myl12b
Q8VC30	Triokinase/FMN cyclase	Tkfc
P68433	Histone H3.1	H3c1
Q69ZH9	Rho GTPase-activating protein 23	Arhgap23
Q62266	Cornifin-A	Sprr1a
P10107	Annexin A1	Anxa1
Q3UMT1	Protein phosphatase 1 regulatory subunit 12C	Ppp1r12c
Q9D0R8	Protein LSM12 homolog	Lsm12
Q05020	Apolipoprotein C-II	Apoc2
Q91XV3	Brain acid soluble protein 1	Basp1
Q8VHQ9	Acyl-coenzyme A thioesterase 11	Acot11
P07356	Annexin A2	Anxa2
Q8CFE4	SCY1-like protein 2	Scyl2
P43883	Perilipin-2	Plin2
Q9CQE5	Regulator of G-protein signaling 10	Rgs10
P35459	Lymphocyte antigen 6D	Ly6d
A2A7S8	Uncharacterized protein KIAA1522	Kiaa1522

P70296	Phosphatidylethanolamine-binding protein 1	Pebp1
Q6R2P8	Endonuclease 8-like 2	Neil2
P09813	Apolipoprotein A-II	Apoa2
P10637	Microtubule-associated protein tau	Mapt
Q7TSI3	Serine/threonine-protein phosphatase 6 regulatory subunit 1	Ppp6r1
P56655	Cytochrome P450 2C38	Cyp2c38
P19324	Serpin H1	Serpinh1
Q3V0K9	Plastin-1	Pls1

The liver samples (from 5 mice in each group) were individually assessed by TMT-based differential proteomic expression and data were merged to get averages. Protein FDR Confidence for all proteins was ≤2%. FDR: False Discovery Rate. These altered proteins were downregulated compared to the high-fat control group (MS-NASH mice on a high-fat diet) with a 2-fold-change threshold. These data (Venn diagrams) are shown in Figure 3A.
