# Supplementary material for: Liver Protein Expression in NASH Mice on a High-Fat Diet: Response to Multi-Mineral Intervention
Source: Front Nutr. 2022 May 11;9:859292. doi: 10.3389/fnut.2022.859292 (PMC9130755; doi:10.3389/fnut.2022.859292)
Supplement: Supplementary Table 1 — Mineral Composition of Aquamin® Soluble. [file Data_Sheet_1.zip › SM Table 4 859292.pdf]

Supplement Table 4. Significantly altered (Upregulated) proteins with Aquamin in high-fat mice

Proteins	Genes	MS-NASH Aquamin	MS-NASH OCA	C57BL6 (Low-Fat)
UDP-N-acetylhexosamine pyrophosphorylase-like protein 1	Uap1l1	*1.9±0.8	0.9±0.4	1.1±0.2
Biglycan	Bgn	*1.7±0.5	1.5±0.6	0.8±0.3
Tax1-binding protein 3	Tax1bp3	*1.6±0.5	1.6±1.1	1.1±0.5
Succinyl-CoA:3-ketoacid coenzyme A transferase 1, mitochondrial	Oxct1	*1.6±0.3	*1.4±0.2	1.2±0.4
Glycogen phosphorylase, brain form	Pygb	*1.5±0.3	0.9±0.2	0.6±0.1
Acyl-coenzyme A thioesterase 9, mitochondrial	Acot9	*1.5±0.5	1.3±0.6	0.8±0.6
CD166 antigen	Alcam	*1.5±0.4	1.3±0.5	*1.2±0.2
Erythrocyte band 7 integral membrane protein	Stom	*1.5±0.4	1.2±0.5	0.9±0.5
RNA-binding protein 3	Rbm3	*1.5±0.3	*1.4±0.4	1.2±0.3
Angiotensinogen	Agt	*1.5±0.4	1.1±0.3	*1.5±0.4
Apolipoprotein A-I	Apoa1	*1.5±0.3	1.0±0.3	0.8±0.2
Heparin cofactor 2	Serpind1	*1.5±0.4	1.2±0.6	1.1±0.4
Endonuclease/exonuclease/phosphatase family domain-containing protein 1	Eepd1	*1.4±0.4	1.4±0.5	0.9±0.4
Galectin-3	Lgals3	*1.4±0.4	0.8±0.3	0.4±0.2
Sterile alpha motif domain-containing protein 9-like	Samd9l	*1.4±0.1	1.2±0.2	0.4±0.1
Annexin A5	Anxa5	*1.4±0.3	1.0±0.3	0.7±0.1
Myoferlin	Myof	*1.4±0.1	1.0±0.2	0.6±0.2
Charged multivesicular body protein 2b	Chmp2b	*1.4±0.2	1.1±0.2	0.4±0.2
ATP-dependent 6-phosphofructokinase, platelet type	Pfkp	*1.4±0.3	0.9±0.3	0.6±0.3
55 kDa erythrocyte membrane protein	Mpp1	*1.4±0.3	1.1±0.2	0.9±0.3
Protein S100-A10	S100a10	*1.4±0.3	0.9±0.4	0.5±0.1
Protein unc-119 homolog B	Unc119b	*1.3±0.2	1.1±0.2	1.1±0.5
Glucose-6-phosphate isomerase	Gpi1	*1.3±0.3	*1.4±0.4	0.7±0.1
Major vault protein	Mvp	*1.3±0.3	1.0±0.1	0.9±0.1
Hexokinase-4	Gck	*1.3±0.3	*1.3±0.1	0.6±0.2
Ankyrin repeat and SOCS box protein 13	Asb13	*1.3±0.1	*1.5±0.2	0.8±0.1
Serine/threonine-protein kinase TBK1	Tbk1	*1.3±0.0	1.2±0.3	1.3±0.2
Annexin A2	Anxa2	*1.3±0.2	0.8±0.3	0.4±0.1
Glutamate--cysteine ligase catalytic subunit	Gclc	*1.3±0.2	1.1±0.1	0.7±0.1
DnaJ homolog subfamily C member 8	Dnajc8	*1.3±0.1	1.1±0.2	1.6±0.9
Coactosin-like protein	Cotl1	*1.3±0.2	1.0±0.2	0.5±0.2
RNA polymerase II-associated factor 1 homolog	Paf1	*1.3±0.1	1.2±0.2	1.1±0.1
Phosphopantothenoylcysteine decarboxylase	Ppcdc	*1.3±0.1	1.4±0.4	2.5±2.1

Cytochrome c oxidase subunit 7A1, mitochondrial	Cox7a1	*1.3±0.1	1.0±0.1	0.8±0.2
Carbonyl reductase [NADPH] 1	Cbr1	*1.3±0.2	*1.5±0.2	1.1±0.4
Rho GDP-dissociation inhibitor 1	Arhgdia	*1.3±0.0	1.1±0.2	1.0±0.1
Alpha-2-HS-glycoprotein	Ahsg	*1.3±0.2	1.1±0.1	1.0±0.3
COMM domain-containing protein 4	Commd4	*1.3±0.1	1.1±0.4	0.8±0.1
Xaa-Pro dipeptidase	Pepd	*1.3±0.2	1.2±0.2	0.7±0.1
Fascin	Fscn1	*1.3±0.2	0.9±0.2	0.9±0.3
Exopolyphosphatase PRUNE1	Prune1	*1.3±0.2	1.1±0.2	0.6±0.1
Afamin	Afm	*1.3±0.2	1.1±0.1	0.9±0.4
C4b-binding protein	C4bp	*1.3±0.2	0.8±0.2	0.9±0.2
Transketolase	Tkt	*1.3±0.2	*1.4±0.3	1.0±0.2
Pyruvate kinase PKLR	Pklr	*1.3±0.2	*1.5±0.2	0.8±0.2
Myosin light polypeptide 6	Myl6	*1.3±0.2	1.1±0.5	0.6±0.3
Glutathione S-transferase Mu 3	Gstm3	*1.3±0.2	1.2±0.4	0.8±0.3
WD repeat-containing protein 1	Wdr1	*1.3±0.2	1.1±0.3	1.0±0.3
Probable rRNA-processing protein EBP2	Ebna1bp2	*1.3±0.2	*1.2±0.1	1.0±0.0
Profilin-1	Pfn1	*1.2±0.2	1.1±0.2	1.0±0.1
Probable tRNA N6-adenosine threonylcarbamoyltransferase	Osgep	*1.2±0.2	1.0±0.2	0.8±0.2
EKC/KEOPS complex subunit Tp53rk	Trp53rk	*1.2±0.1	1.1±0.1	1.4±0.7
Phospholipid scramblase 1	Plscr1	*1.2±0.1	*1.2±0.1	0.8±0.2
Protein LSM14 homolog A	Lsm14a	*1.2±0.2	*1.2±0.2	0.7±0.2
Pyrroline-5-carboxylate reductase 3	Pycrl	*1.2±0.2	1.0±0.2	*1.4±0.3
Glutathione S-transferase A1	Gsta1	*1.2±0.1	*1.5±0.1	0.5±0.1
Striatin-3	Strn3	*1.2±0.1	*1.3±0.1	1.0±0.2
Glycogen synthase kinase-3 alpha	Gsk3a	*1.2±0.2	1.2±0.4	0.5±0.3
Serum albumin	Alb	*1.2±0.1	1.1±0.2	0.8±0.2
Fibulin-1	Fbln1	*1.2±0.1	0.9±0.1	0.9±0.3
Small ubiquitin-related modifier 1	Sumo1	*1.2±0.1	1.0±0.1	0.9±0.1
DnaJ homolog subfamily C member 10	Dnajc10	*1.2±0.1	1.0±0.2	1.0±0.2
Cyclin-dependent kinase 6	Cdk6	*1.2±0.2	1.2±0.2	0.8±0.4
Ragulator complex protein LAMTOR5	Lamtor5	*1.2±0.0	1.0±0.1	*1.3±0.1
Microtubule-associated protein RP/EB family member 3	Mapre3	*1.2±0.2	0.9±0.2	0.9±0.1
Abscission/NoCut checkpoint regulator	Zfyve19	*1.2±0.1	1.1±0.3	0.8±0.1
Rho guanine nucleotide exchange factor 7	Arhgef7	*1.2±0.1	1.1±0.2	0.9±0.1
Synaptic vesicle membrane protein VAT-1 homolog	Vat1	*1.2±0.1	1.0±0.2	1.0±0.2
Carbonyl reductase [NADPH] 3	Cbr3	*1.2±0.2	1.1±0.3	0.8±0.3
m7GpppX diphosphatase	Dcps	*1.2±0.2	*1.2±0.2	*1.5±0.2

Pre-rRNA-processing protein TSR2 homolog	Tsr2	*1.2±0.2	1.1±0.1	0.8±0.3
Adenylyl cyclase-associated protein 1	Cap1	*1.2±0.1	1.0±0.1	0.8±0.1
Endothelin-converting enzyme 1	Ece1	*1.2±0.1	1.1±0.2	1.1±0.2
Cytoplasmic FMR1-interacting protein 1	Cyfip1	*1.2±0.2	1.0±0.1	0.8±0.2
H-2 class I histocompatibility antigen, Q10 alpha chain	H2-Q10	*1.2±0.1	0.9±0.1	0.6±0.0
Nuclear autoantigenic sperm protein	Nasp	*1.2±0.1	1.0±0.1	1.2±0.4
Protein C10	Grcc10	*1.2±0.2	1.4±0.9	1.6±0.9
Zinc-alpha-2-glycoprotein	Azgp1	*1.2±0.1	0.9±0.1	1.0±0.1
Chromobox protein homolog 1	Cbx1	*1.2±0.2	1.1±0.1	0.8±0.2
CCR4-NOT transcription complex subunit 10	Cnot10	*1.2±0.1	1.1±0.1	1.1±0.3
TBC1 domain family member 22A	Tbc1d22a	*1.2±0.1	1.1±0.3	0.6±0.5
Bis(5'-nucleosyl)-tetraphosphatase [asymmetrical]	Nudt2	*1.2±0.2	1.1±0.1	1.0±0.2
Paired amphipathic helix protein Sin3a	Sin3a	*1.2±0.1	1.1±0.2	2.0±2.1
Alpha-actinin-4	Actn4	*1.2±0.2	1.0±0.2	0.9±0.2
Ubiquitin-like modifier-activating enzyme 5	Uba5	*1.2±0.2	1.1±0.1	0.7±0.3
Solute carrier family 23 member 1	Slc23a1	*1.2±0.1	1.0±0.1	0.8±0.0
Leukocyte surface antigen CD47	Cd47	*1.2±0.1	1.0±0.1	*1.3±0.3
Ubiquitin thioesterase OTUB1	Otub1	*1.2±0.1	1.1±0.1	0.8±0.2
Craniofacial development protein 1	Cfdp1	*1.2±0.2	1.1±0.1	0.6±0.1
Osteoclast-stimulating factor 1	Ostf1	*1.2±0.2	1.0±0.1	0.7±0.1
Protein FAM107B	Fam107b	*1.2±0.1	1.0±0.1	0.9±0.2
ATP-dependent 6-phosphofructokinase, liver type	Pfkl	*1.2±0.1	*1.2±0.1	0.7±0.2
39S ribosomal protein L55, mitochondrial	Mrpl55	*1.2±0.0	1.0±0.0	1.2±0.2
Heterogeneous nuclear ribonucleoprotein U-like protein 1	Hnrnpul1	*1.2±0.1	1.1±0.1	1.0±0.1
Protein kinase C and casein kinase substrate in neurons protein 2	Pacsin2	*1.2±0.1	1.1±0.1	1.0±0.1
ATPase inhibitor, mitochondrial	Atpif1	*1.2±0.1	0.9±0.2	0.8±0.1
Alpha-enolase	Eno1	*1.2±0.1	*1.2±0.2	0.7±0.1
Alpha-actinin-1	Actn1	*1.2±0.1	1.0±0.1	0.8±0.1
COMM domain-containing protein 1	Commd1	*1.2±0.1	0.9±0.2	1.2±0.3
High mobility group protein B1	Hmgb1	*1.2±0.1	1.1±0.1	1.1±0.2
Gamma-glutamylcyclotransferase	Ggct	*1.2±0.1	1.0±0.1	0.8±0.2
5'-AMP-activated protein kinase catalytic subunit alpha-2	Prkaa2	*1.2±0.1	*1.4±0.2	0.7±0.1
CTP synthase 1	Ctps	*1.2±0.1	0.9±0.2	0.8±0.2
UBX domain-containing protein 1	Ubxn1	*1.2±0.1	1.0±0.2	1.1±0.3
DNA-(apurinic or apyrimidinic site) lyase	Apex1	*1.2±0.1	1.1±0.1	*1.3±0.1
Serine/threonine-protein phosphatase 2A catalytic subunit alpha isoform	Ppp2ca	*1.2±0.1	1.1±0.1	1.1±0.1
Beta-2-glycoprotein 1	Apoh	*1.2±0.1	0.9±0.1	0.8±0.2

Interferon-induced, double-stranded RNA-activated protein kinase	Eif2ak2	*1.2±0.1	1.0±0.2	1.2±0.2
RNA-binding protein FUS	Fus	*1.2±0.1	1.0±0.2	1.1±0.2
Mini-chromosome maintenance complex-binding protein	Mcmbp	*1.1±0.1	1.2±0.3	1.0±0.2
Receptor-interacting serine/threonine-protein kinase 1	Ripk1	*1.1±0.1	1.0±0.1	1.1±0.3
BMP-2-inducible protein kinase	Bmp2k	*1.1±0.1	1.2±0.3	0.7±0.3
FACT complex subunit SPT16	Supt16h	*1.1±0.1	1.0±0.1	1.1±0.2
U6 snRNA-associated Sm-like protein LSm6	Lsm6	*1.1±0.1	1.0±0.2	1.1±0.2
Protein S100-A4	S100a4	*1.1±0.0	0.9±0.7	1.0±0.4
pre-mRNA 3' end processing protein WDR33	Wdr33	*1.1±0.0	1.1±0.1	1.5±0.7
High affinity immunoglobulin gamma Fc receptor I	Fcgr1	*1.1±0.1	1.0±0.3	0.5±0.1
U6 snRNA-associated Sm-like protein LSm3	Lsm3	*1.1±0.1	0.9±0.1	1.1±0.2
Hepatocyte nuclear factor 4-alpha	Hnf4a	*1.1±0.1	*1.2±0.1	1.2±0.4
Ras GTPase-activating protein-binding protein 1	G3bp1	*1.1±0.1	1.0±0.1	0.9±0.1
Brain-specific angiogenesis inhibitor 1-associated protein 2	Baiap2	*1.1±0.1	*1.3±0.2	*1.3±0.2
Neutral cholesterol ester hydrolase 1	Nceh1	*1.1±0.1	0.7±0.2	0.8±0.1
Heterogeneous nuclear ribonucleoprotein U	Hnrnpu	*1.1±0.1	1.1±0.1	0.9±0.1
Sorting nexin-4	Nceh1	*1.1±0.1	0.9±0.1	0.7±0.2
Cell cycle and apoptosis regulator protein 2	Ccar2	*1.1±0.1	1.0±0.1	0.9±0.1
Host cell factor 1	Hcfc1	*1.1±0.1	1.0±0.2	0.8±0.2
UDP-glucuronic acid/UDP-N-acetylgalactosamine transporter	Slc35d1	*1.1±0.1	1.0±0.1	0.9±0.5
Fatty acid-binding protein, intestinal	Fabp2	*1.1±0.1	*1.2±0.1	0.4±0.1
tRNA pseudouridine synthase A	Pus1	*1.1±0.1	1.1±0.2	1.0±0.2
Alpha-2-antiplasmin	Serpinf2	*1.1±0.1	1.0±0.1	0.9±0.1
Ras-related protein Rab-5C	Rab5c	*1.1±0.1	1.0±0.1	0.9±0.2
Guanine nucleotide-binding protein G(i) subunit alpha-2	Gnai2	*1.1±0.1	0.9±0.2	0.8±0.2
Heterogeneous nuclear ribonucleoprotein D0	Hnrnpd	*1.1±0.1	1.1±0.2	*1.1±0.1
Presequence protease, mitochondrial	Pitrm1	*1.1±0.1	1.0±0.1	1.0±0.1
14-3-3 protein epsilon	Ywhae	*1.1±0.1	*1.1±0.1	1.0±0.1
Dynactin subunit 5	Dctn5	*1.1±0.0	0.9±0.1	0.8±0.3
Actin, cytoplasmic 1	Actb	*1.1±0.1	1.0±0.2	0.8±0.3
Signal transducer and activator of transcription 5B	Stat5b	*1.1±0.1	1.1±0.2	0.8±0.2
Phosphatidylinositol transfer protein alpha isoform	Pitpna	*1.1±0.1	1.0±0.1	0.9±0.1
Septin-2	Sept2	*1.1±0.1	1.0±0.1	0.9±0.1
L-fucose kinase	Fuk	*1.1±0.1	1.1±0.2	0.8±0.3
6-phosphogluconate dehydrogenase, decarboxylating	Pgd	*1.1±0.1	1.1±0.1	0.6±0.1
Wiskott-Aldrich syndrome protein family member 2	Wasf2	*1.1±0.1	0.9±0.1	0.6±0.1
Ubiquitin-conjugating enzyme E2 H	Ube2h	*1.1±0.1	1.0±0.1	0.9±0.1

Calcyclin-binding protein	Cacybp	*1.1±0.1	*1.1±0.1	1.0±0.1
BAG family molecular chaperone regulator 1	Bag1	*1.1±0.1	1.0±0.1	0.8±0.1
RNA-binding Raly-like protein	Ralyl	*1.1±0.0	0.9±0.1	1.1±0.1
Tubulin alpha-1C chain	Tuba1c	*1.1±0.1	1.0±0.1	0.7±0.2
Pre-mRNA-splicing factor ATP-dependent RNA helicase DHX15	Dhx15	*1.1±0.1	*1.1±0.1	1.0±0.2
NEDD8-activating enzyme E1 catalytic subunit	Uba3	*1.1±0.1	1.0±0.1	0.8±0.1
Tumor protein D54	Tpd52l2	*1.1±0.1	1.0±0.1	0.9±0.1
Ras-related C3 botulinum toxin substrate 1	Rac1	*1.1±0.1	1.0±0.1	1.0±0.1
Desmoglein-2	Dsg2	*1.1±0.1	1.1±0.1	1.0±0.1
Serine/threonine-protein phosphatase PP1-alpha catalytic subunit	Ppp1ca	*1.1±0.1	1.1±0.1	1.0±0.1
Paxillin	Pxn	*1.1±0.1	*1.1±0.0	0.8±0.1
Heat shock 70 kDa protein 4	Hspa4	*1.1±0.1	*1.1±0.0	1.0±0.1
Hsc70-interacting protein	St13	*1.1±0.1	1.0±0.1	1.1±0.2
Spliceosome RNA helicase Ddx39b	Ddx39b	*1.1±0.1	1.0±0.1	0.9±0.2
Carboxy-terminal domain RNA polymerase II polypeptide A small phosphatase 1	Ctdsp1	*1.1±0.1	1.0±0.1	1.0±0.1
Soluble calcium-activated nucleotidase 1	Cant1	*1.1±0.0	0.9±0.1	0.9±0.0
Coagulation factor XIII B chain	F13b	*1.1±0.1	1.0±0.2	0.8±0.2
Proteasome subunit beta type-1	Psmb1	*1.1±0.1	1.0±0.1	1.3±0.3
Nck-associated protein 1	Nckap1	*1.1±0.1	1.0±0.0	0.8±0.1
Platelet-activating factor acetylhydrolase IB subunit alpha	Pafah1b1	*1.1±0.1	1.0±0.1	0.9±0.1
SUMO-conjugating enzyme UBC9	Ube2i	*1.1±0.1	1.0±0.1	0.8±0.1
Proteasome subunit alpha type-3	Psma3	*1.1±0.1	1.0±0.1	1.2±0.3
Dystrophin	Dmd	*1.1±0.1	1.0±0.2	1.0±0.2
Threonine--tRNA ligase, mitochondrial	Tars2	*1.1±0.0	0.9±0.2	1.2±0.5
GTPase NRas	Nras	*1.1±0.0	1.1±0.1	0.9±0.2
Acylamino-acid-releasing enzyme	Apeh	*1.1±0.1	*1.3±0.1	1.1±0.3
Basigin	Bsg	*1.1±0.0	1.0±0.1	0.8±0.1
E3 ubiquitin-protein ligase HUWE1	Huwe1	*1.1±0.0	1.0±0.0	0.9±0.2
Kinesin-1 heavy chain	Kif5b	*1.1±0.1	1.1±0.1	0.9±0.1
TP53-regulated inhibitor of apoptosis 1	Triap1	*1.1±0.0	1.0±0.2	1.1±0.1
CapZ-interacting protein	Rcsd1	*1.1±0.1	0.9±0.1	0.9±0.3
Tripartite motif-containing protein 14	Trim14	*1.1±0.0	*1.2±0.2	0.9±0.2
Filamin-B	Flnb	*1.1±0.1	0.9±0.1	0.8±0.1
Conserved oligomeric Golgi complex subunit 8	Cog8	*1.1±0.0	0.9±0.1	1.2±0.3
Trafficking protein particle complex subunit 11	Trappc11	*1.1±0.0	1.0±0.1	0.9±0.2
Transmembrane 9 superfamily member 2	Tm9sf2	*1.1±0.1	0.9±0.0	1.1±0.2
RuvB-like 2	Ruvbl2	*1.1±0.1	1.0±0.1	0.9±0.2

Probable ATP-dependent RNA helicase DDX5	Ddx5	*1.1±0.1	1.0±0.1	1.0±0.2
Exportin-1	Xpo1	*1.1±0.0	1.0±0.0	0.8±0.2

These values represent average (± standard deviation) fold-change of abundance ratios for each altered (upregulated) protein compared to the high-fat control group (MS-NASH mice on a high-fat diet) with a 1.1-fold change threshold in response to Aquamin intervention and are significant with a p-value <0.05 (*). For each upregulated protein with Aquamin, corresponding values from the other two groups are shown for comparison. These liver samples (from 5 mice in each group) were individually assessed by TMT-based differential proteomic expression and data were merged to get averages. These data are also presented in Figure 4.
