# Supplementary material for: Liver Protein Expression in NASH Mice on a High-Fat Diet: Response to Multi-Mineral Intervention
Source: Front Nutr. 2022 May 11;9:859292. doi: 10.3389/fnut.2022.859292 (PMC9130755; doi:10.3389/fnut.2022.859292)
Supplement: Supplementary Table 1 — Mineral Composition of Aquamin® Soluble. [file Data_Sheet_1.zip › SM Table 5 859292.pdf]

Supplement Table 5. Top pathways associated with significantly upregulated proteins (shown in S Table 4) altered with Aquamin

Pathway name	Entities pValue	Mapped entities
RHO GTPases Activate WASPs and WAVEs	1.01×10^{-4}	Rac1;Wasf2;Cyfip1;Nckap1;Baiap2;Actb
Neutrophil degranulation	3.99×10^{-4}	Ahsg;Stom;Mvp;Gpi1;Pygb;Cd47;Cant1;Rab5c;Ostf1;Vat1;Cap1;Rac1;Pfkl;Hmgb1;Cyfip1; Lgals3;Huwe1;Cotl1;H2-Q10;Apeh;Anxa2;Nras;Psmb1
VEGFA-VEGFR2 Pathway	6.09×10^{-4}	Rac1;Wasf2;Cyfip1;Nckap1;Baiap2;Pxn;Nras;Actb
Platelet degranulation	8.98×10^{-4}	Ahsg;Serpinf2;Actn1;Alb;Apoh;Anxa5;Apoa1;Actn4;Wdr1
Signaling by VEGF	0.001	Rac1;Wasf2;Cyfip1;Nckap1;Baiap2;Pxn;Nras;Actb
Response to elevated platelet cytosolic Ca2+	0.001	Ahsg;Serpinf2;Actn1;Alb;Apoh;Anxa5;Apoa1;Actn4;Wdr1
Glycolysis	0.002	Pfkl;Gpi1;Eno1;Gck;Pfkp;Pklr
RHO GTPase Effectors	0.002	Wasf2;Tax1bp3;Nckap1;Kif5b;Baiap2;Tuba1c;Rac1;Cyfip1;Ppp2ca;Myl6;Pafah1b1;Pfn1;Ywhae; Actb
Dissolution of Fibrin Clot	0.002	Serpinf2;S100a10;Anxa2
PTK6 Regulates RHO GTPases, RAS GTPase and MAP kinases	0.003	Rac1;Pxn;Nras
SUMOylation of DNA methylation proteins	0.003	Sumo1;Ube2i
mRNA Splicing - Major Pathway	0.004	Wdr33;Ddx5;Lsm3;Hnrnpd;Dhx15;Dnajc8;Fus;Hnrnpu;Hnrnpul1;Lsm6
SUMO is transferred from E1 to E2 (UBE2I, UBC9)	0.005	Sumo1;Ube2i
mRNA Splicing	0.005	Wdr33;Ddx5;Lsm3;Hnrnpd;Dhx15;Dnajc8;Fus;Hnrnpu;Hnrnpul1;Lsm6
Cell-extracellular matrix interactions	0.006	Actn1;Pxn;Actb
Innate Immune System	0.006	Ahsg;Stom;Psma3;Mvp;Nckap1;Baiap2;Cd47;Rab5c;Tbk1;Ostf1;Vat1;Cap1;Rac1;Uba3;Pfkl; Cyfip1;Huwe1;H2-Q10;Apeh;Anxa2;Psmb1;Actb;Wasf2;Gpi1;Fcgr1;Ripk1;Pygb;Cant1; Hmgb1;Lgals3;Ppp2ca;Cotl1;Nras
Glucose metabolism	0.007	Pfkl;Gpi1;Eno1;Gck;Pfkp;Pklr
SUMOylation of transcription cofactors	0.008	Sin3a;Sumo1;Ddx5;Ube2i
Nuclear Envelope (NE) Reassembly	0.008	Sumo1;Ppp2ca;Ube2i;Tuba1c;Chmp2b
Processing of Capped Intron-Containing Pre-mRNA	0.010	Wdr33;Ddx39b;Ddx5;Lsm3;Hnrnpd;Dhx15;Dnajc8;Fus;Hnrnpu;Hnrnpul1;Lsm6
Regulation of cytoskeletal remodeling and cell spreading by IPP complex components	0.012	Actn1;Pxn
Regulation of HSF1-mediated heat shock response	0.013	Hspa4;St13;Bag1;Ccar2;Ywhae

Platelet activation, signaling and aggregation	0.014	Ahsg;Rac1;Serpinf2;Actn1;Alb;Apoh;Anxa5;Apoa1;Actn4;Wdr1;Gnai2
HDL remodeling	0.014	Alb;Apoa1
Negative regulation of activity of TFAP2 (AP-2) family transcription factors	0.014	Sumo1;Ube2i
Processing and activation of SUMO	0.018	Sumo1;Ube2i
Hemostasis	0.018	Ahsg;Serpinf2;Actn1;S100a10;Alb;Kif5b;Cd47;Anxa5;Bsg;Tuba1c;Serpind1;Rac1;Apoh;F13b;Apoa1;Actn4;Ppp2ca;Anxa2;Nras;Wdr1;Gnai2
SUMOylation of immune response proteins	0.021	Sumo1;Ube2i
RHO GTPases activate KTN1	0.021	Rac1;Kif5b
RHO GTPases activate IQGAPs	0.025	Rac1;Actb
Glutathione synthesis and recycling	0.025	Ggct;Gclc
Deadenylation-dependent mRNA decay	0.026	Dcps;Lsm3;Cnot10;Lsm6
Pentose phosphate pathway	0.029	Tkt;Pgd
Glutathione conjugation	0.030	Ggct;Gclc;Gstm3
RAC2 GTPase cycle	0.032	Dsg2;Wasf2;Cyfip1;Nckap1;Arhgdia
mRNA decay by 5' to 3' exoribonuclease	0.033	Lsm3;Lsm6
Cellular response to heat stress	0.033	Hspa4;St13;Bag1;Ccar2;Ywhae
SUMOylation of transcription factors	0.037	Sumo1;Ube2i
RAC3 GTPase cycle	0.039	Dsg2;Wasf2;Cyfip1;Nckap1;Baiap2
Abasic sugar-phosphate removal via the single-nucleotide replacement pathway	0.039	Apex1
Insulin effects increased synthesis of Xylulose-5-Phosphate	0.039	Tkt
Interleukin-37 signaling	0.039	Tbk1
Signaling by Rho GTPases	0.047	Arhgef7;Wasf2;Stom;Actn1;Ddx39b;Tax1bp3;Nckap1;Kif5b;Baiap2;Arhgdia;Tuba1c;Dsg2;Rac1;Cyfip1;Ppp2ca;Myl6;Pafah1b1;Pfn1;Ywhae;Actb

The pathways listed here are altered by the significantly upregulated proteins with the intervention “Aquamin” presented in Supplement Table 4. Reactome (v78) was used to generate the pathway analysis report for species *Mus musculus.* The significance (p-value) is calculated by the overrepresentation analysis (hypergeometric distribution).
