# Supplementary material for: Liver Protein Expression in NASH Mice on a High-Fat Diet: Response to Multi-Mineral Intervention
Source: Front Nutr. 2022 May 11;9:859292. doi: 10.3389/fnut.2022.859292 (PMC9130755; doi:10.3389/fnut.2022.859292)
Supplement: Supplementary Table 1 — Mineral Composition of Aquamin® Soluble. [file Data_Sheet_1.zip › SM Table 6 859292.pdf]

Supplement Table 6. Significantly altered (Downregulated) proteins with Aquamin in high-fat mice

Proteins	Genes	MS-NASH Aquamin	MS-NASH OCA	C57BL6 (Low-Fat)
Cytochrome P450 2C54	Cyp2c54	*0.48±0.14	*0.61±0.19	2.72±0.49
Major intrinsically disordered NOTCH2-binding receptor 1-like homolog	Minar2	*0.49±0.12	*0.45±0.18	1.06±0.23
Alpha-1-antitrypsin 1-5	Serpina1e	*0.52±0.27	0.70±0.35	37.05±11.87
Protein CASC3	Casc3	*0.57±0.13	*0.55±0.16	*0.60±0.22
Major urinary protein 2	Mup2	*0.59±0.23	*0.44±0.19	10.40±5.30
Cysteine dioxygenase type 1	Cdo1	*0.59±0.18	*0.54±0.11	1.94±0.66
Sodium-coupled neutral amino acid transporter 4	Slc38a4	*0.60±0.27	*0.62±0.27	1.30±0.37
Ferritin light chain 1	Ftl1	*0.62±0.23	0.74±0.27	1.18±0.80
40S ribosomal protein S30	Fau	*0.63±0.33	0.71±0.26	0.80±0.42
Sodium-coupled neutral amino acid transporter 3	Slc38a3	*0.63±0.13	0.71±0.18	2.60±1.10
2-oxo-4-hydroxy-4-carboxy-5-ureidoimidazoline decarboxylase	Urad	*0.64±0.20	0.94±0.20	3.19±2.07
Cytochrome P450 2C50	Cyp2c50	*0.65±0.22	0.79±0.40	4.82±2.53
5'-nucleotidase	Nt5e	*0.66±0.28	*0.67±0.19	1.33±0.30
Ileal sodium/bile acid cotransporter	Slc10a2	*0.67±0.24	0.77±0.22	*0.54±0.27
Ethanolamine-phosphate phospho-lyase	Etnppl	*0.69±0.26	1.55±0.67	1.37±0.38
Cytochrome P450 2C29	Cyp2c29	*0.69±0.25	*0.74±0.24	1.68±0.81
Leucine-rich repeat-containing protein 9	Lrrc9	*0.71±0.17	0.98±0.23	*0.05±0.01
Histidine ammonia-lyase	Hal	*0.71±0.27	2.54±0.83	1.88±0.49
Carboxylesterase 3B	Ces3b	*0.71±0.16	1.34±0.32	5.56±2.65
Magnesium-dependent phosphatase 1	Mdp1	*0.72±0.21	0.83±0.18	0.96±0.13
Hepatocyte nuclear factor 1-alpha	Hnf1a	*0.72±0.21	0.80±0.30	1.18±0.45
Sodium/bile acid cotransporter	Slc10a1	*0.72±0.21	1.21±0.37	1.88±1.04
Aquaporin-9	Aqp9	*0.73±0.23	*0.63±0.09	1.17±0.48
Cytochrome P450 2C37	Cyp2c37	*0.73±0.19	*0.71±0.19	2.55±1.14
Dolichyl-phosphate beta-glucosyltransferase	Alg5	*0.73±0.21	*0.68±0.20	*0.66±0.19
Golgi SNAP receptor complex member 2	Gosr2	*0.73±0.21	*0.75±0.16	0.82±0.29
Cytochrome c oxidase subunit 7A-related protein, mitochondrial	Cox7a2l	*0.74±0.10	*0.73±0.03	1.32±0.06
Isovaleryl-CoA dehydrogenase, mitochondrial	Ivd	*0.74±0.10	0.89±0.12	2.57±0.87
Cytochrome P450 7B1	Cyp7b1	*0.74±0.21	*0.63±0.31	15.31±8.01
Cancer-related nucleoside-triphosphatase homolog	Ntpcr	*0.74±0.19	*0.81±0.18	*0.61±0.16
Cytochrome P450 4A12A	Cyp4a12a	*0.75±0.22	0.75±0.45	0.70±0.32
Cytochrome P450 2C44	Cyp2c23	*0.76±0.12	1.09±0.27	2.50±0.46
Thioredoxin domain-containing protein 15	Txndc15	*0.76±0.11	*0.76±0.14	1.17±0.24

Hermansky-Pudlak syndrome 1 protein homolog	Hps1	*0.76±0.17	0.78±0.43	*0.56±0.15
Vitamin K epoxide reductase complex subunit 1	Vkorc1	*0.76±0.06	*0.86±0.08	1.05±0.14
Homocysteine-responsive endoplasmic reticulum-resident ubiquitin-like domain member 2 protein	Herpud2	*0.76±0.03	0.93±0.05	1.00±0.13
Serine/threonine-protein phosphatase 6 regulatory ankyrin repeat subunit A	Ankrd28	*0.77±0.04	*0.80±0.09	*0.84±0.01
Multiple inositol polyphosphate phosphatase 1	Minpp1	*0.77±0.09	0.87±0.22	*0.75±0.19
Peroxisomal membrane protein 2	Pxmp2	*0.77±0.13	1.03±0.30	1.01±0.28
Calcium uptake protein 1, mitochondrial	Micu1	*0.77±0.02	0.95±0.02	1.06±0.21
Sideroflexin-2	Sfxn2	*0.77±0.11	0.90±0.11	1.81±0.63
Tetratricopeptide repeat protein 36	Ttc36	*0.78±0.09	1.02±0.10	1.19±0.16
Delta(14)-sterol reductase TM7SF2	Tm7sf2	*0.78±0.20	0.85±0.18	2.28±0.76
Hydroxyproline dehydrogenase	Prodh2	*0.78±0.08	0.93±0.16	1.06±0.15
Canalicular multispecific organic anion transporter 1	Abcc2	*0.78±0.19	1.04±0.24	1.03±0.26
28S ribosomal protein S25, mitochondrial	Mrps25	*0.79±0.18	*0.74±0.18	0.97±0.20
Cytochrome P450 2D11	Cyp2d11	*0.79±0.14	0.88±0.31	1.17±0.42
Transmembrane protein 19	Tmem19	*0.79±0.11	*0.83±0.12	0.90±0.24
Methionine-R-sulfoxide reductase B1	Msrb1	*0.79±0.12	0.94±0.13	1.20±0.10
Bifunctional UDP-N-acetylglucosamine 2-epimerase/N-acetylmannosamine kinase	Gne	*0.79±0.14	1.00±0.20	1.38±0.30
Serine protease inhibitor A3K	Serpina3k	*0.80±0.13	0.88±0.16	3.28±0.96
Surfeit locus protein 4	Surf4	*0.80±0.17	*0.70±0.15	*0.67±0.24
Lipoamide acyltransferase component of branched-chain alpha-keto acid dehydrogenase complex, mitochondrial	Dbt	*0.80±0.14	1.04±0.19	1.50±0.25
28S ribosomal protein S12, mitochondrial	Mrps12	*0.80±0.09	0.89±0.18	1.12±0.11
Receptor-type tyrosine-protein phosphatase delta	Ptprd	*0.81±0.08	1.19±0.13	1.67±0.47
Tubulin gamma-1 chain	Tubg1	*0.81±0.14	1.05±0.32	*0.72±0.17
NADH dehydrogenase [ubiquinone] iron-sulfur protein 5	Ndufs5	*0.81±0.15	0.88±0.17	0.91±0.13
Dolichol-phosphate mannosyltransferase subunit 1	Dpm1	*0.81±0.02	*0.80±0.07	*0.81±0.17
Apolipoprotein A-V	Apoa5	*0.81±0.17	1.08±0.23	0.83±0.20
Actin-related protein 2/3 complex subunit 1A	Arpc1a	*0.82±0.12	1.13±0.08	1.32±0.13
Kinesin-like protein KIF16B	Kif16b	*0.82±0.04	1.09±0.49	1.08±0.50
Tensin-2	Tns2	*0.82±0.11	0.88±0.12	0.95±0.33
Ferrochelatase, mitochondrial	Fech	*0.82±0.15	0.95±0.13	1.22±0.19
Mitochondrial 2-oxodicarboxylate carrier	Slc25a21	*0.82±0.06	0.95±0.12	0.81±0.16
ATP-binding cassette sub-family B member 10, mitochondrial	Abcb10	*0.82±0.08	1.02±0.13	1.28±0.13
Probable N-acetyltransferase CML1	Cml1	*0.82±0.06	1.28±0.28	1.19±0.35
Short/branched chain specific acyl-CoA dehydrogenase, mitochondrial	Acadsb	*0.82±0.06	1.02±0.15	1.70±0.44

Sulfite oxidase, mitochondrial	Suox	*0.82±0.10	1.07±0.20	1.26±0.29
NADPH-dependent 3-keto-steroid reductase Hsd3b5	Hsd3b5	*0.83±0.12	0.78±0.26	19.97±21.84
Serine--pyruvate aminotransferase, mitochondrial	Agxt	*0.83±0.15	1.08±0.11	2.55±1.07
Mannose-binding protein C	Mbl2	*0.83±0.12	0.95±0.07	1.24±0.19
Hypoxia up-regulated protein 1	Hyou1	*0.83±0.14	*0.81±0.10	1.40±0.27
Inhibin beta C chain	Inhbc	*0.83±0.08	*0.80±0.10	*0.79±0.13
Prolactin regulatory element-binding protein	Preb	*0.83±0.16	*0.79±0.18	0.87±0.23
Calcium load-activated calcium channel	Tmco1	*0.83±0.16	*0.80±0.18	*0.69±0.23
ER membrane protein complex subunit 6	Emc6	*0.84±0.04	0.84±0.28	*0.71±0.06
Kinesin-like protein KIF13A	Kif13a	*0.84±0.15	1.23±0.53	0.98±0.29
Growth factor receptor-bound protein 7	Grb7	*0.84±0.12	*0.91±0.07	0.98±0.26
Probable D-lactate dehydrogenase, mitochondrial	Ldhd	*0.84±0.11	*0.75±0.09	1.38±0.47
Protein disulfide isomerase Creld1	Creld1	*0.84±0.05	*0.81±0.05	1.50±0.43
Transmembrane protein 106B	Tmem106b	*0.84±0.08	0.80±0.14	*0.67±0.08
Cytochrome b5 type B	Cyb5b	*0.84±0.08	*0.71±0.17	1.36±0.63
Sulfotransferase family cytosolic 1B member 1	Sult1b1	*0.85±0.07	1.32±0.18	0.90±0.22
Atlastin-2	Atl2	*0.85±0.13	*0.81±0.08	*0.79±0.10
ADP-ribosylation factor-like protein 6-interacting protein 1	Arl6ip1	*0.85±0.09	*0.70±0.09	0.86±0.15
Ethanolamine kinase 2	Etnk2	*0.85±0.06	1.08±0.19	1.38±0.39
Long-chain fatty acid transport protein 4	Slc27a4	*0.85±0.11	*0.63±0.09	0.76±0.31
Monoacylglycerol lipase ABHD6	Abhd6	*0.86±0.05	*0.73±0.10	*0.76±0.14
39S ribosomal protein L20, mitochondrial	Mrpl20	*0.86±0.09	0.96±0.27	1.53±0.70
Methylcrotonoyl-CoA carboxylase beta chain, mitochondrial	Mccc2	*0.86±0.07	1.00±0.06	1.45±0.15
ATP synthase protein 8	ATP8	*0.86±0.12	1.01±0.06	0.95±0.27
Probable ATP-dependent RNA helicase DHX58	Dhx58	*0.86±0.06	0.84±0.23	0.67±0.24
Perilipin-2	Plin2	*0.86±0.11	*0.56±0.10	*0.18±0.01
Methylcrotonoyl-CoA carboxylase subunit alpha, mitochondrial	Mccc1	*0.86±0.09	1.02±0.13	1.30±0.28
Serine beta-lactamase-like protein LACTB, mitochondrial	Lactb	*0.87±0.07	*0.88±0.06	1.12±0.13
Emopamil-binding protein-like	Ebpl	*0.87±0.12	*0.72±0.11	*0.38±0.21
Sideroflexin-5	Sfxn5	*0.87±0.07	0.96±0.10	*0.71±0.14
Secretory carrier-associated membrane protein 3	Scamp3	*0.87±0.11	0.94±0.16	1.13±0.50
Adenosylhomocysteinase	Ahcy	*0.87±0.12	1.21±0.18	1.33±0.35
Methylmalonic aciduria type A homolog, mitochondrial	Mmaa	*0.87±0.12	1.00±0.22	0.97±0.26
Histidine triad nucleotide-binding protein 3	Hint3	*0.87±0.05	*0.87±0.13	1.02±0.06
Transmembrane protein 143	Tmem143	*0.87±0.08	0.91±0.11	*0.72±0.18
Protein jagunal homolog 1	Jagn1	*0.87±0.08	*0.86±0.06	0.91±0.20
ATP synthase subunit epsilon, mitochondrial	Atp5e	*0.87±0.07	0.90±0.13	0.89±0.11

Protein phosphatase methylesterase 1	Ppme1	*0.87±0.09	0.97±0.12	0.60±0.19
Transmembrane protein 205	Tmem205	*0.87±0.05	*0.83±0.14	1.52±0.33
Methionine aminopeptidase 1	Metap1	*0.88±0.08	1.01±0.19	*0.90±0.05
Acyl-coenzyme A synthetase ACSM5, mitochondrial	Acsm5	*0.88±0.09	1.01±0.12	1.51±0.29
Putative peptidyl-tRNA hydrolase PTRHD1	Ptrhd1	*0.88±0.07	0.95±0.20	*0.63±0.28
60S ribosomal protein L11	Rpl11	*0.88±0.10	*0.86±0.12	0.99±0.30
Dolichol-phosphate mannosyltransferase subunit 3	Dpm3	*0.88±0.08	*0.85±0.09	0.91±0.12
Syndecan-1	Sdc1	*0.88±0.11	0.92±0.17	*0.69±0.16
MICOS complex subunit Mic10	Minos1	*0.89±0.08	*0.83±0.14	1.13±0.33
Equilibrative nucleoside transporter 1	Slc29a1	*0.89±0.06	1.05±0.15	1.59±0.34
Growth hormone receptor	Ghr	*0.89±0.07	1.01±0.16	0.89±0.06
28S ribosomal protein S6, mitochondrial	Mrps6	*0.89±0.06	1.00±0.05	1.13±0.22
Glutaredoxin-related protein 5, mitochondrial	Glrx5	*0.89±0.08	0.98±0.13	0.96±0.15
Peptidyl-prolyl cis-trans isomerase F, mitochondrial	Ppif	*0.89±0.05	1.02±0.10	1.17±0.21
Complex I intermediate-associated protein 30, mitochondrial	Ndufaf1	*0.90±0.05	1.08±0.14	1.39±0.12
Protein disulfide-isomerase A4	Pdia4	*0.90±0.10	*0.78±0.03	1.33±0.12
Succinate--CoA ligase [ADP-forming] subunit beta, mitochondrial	Sucla2	*0.90±0.05	1.01±0.06	0.95±0.24
FERM, ARHGEF and pleckstrin domain-containing protein 2	Farp2	*0.90±0.04	0.95±0.11	1.04±0.21
Elongation factor G, mitochondrial	Gfm1	*0.90±0.09	1.01±0.10	0.98±0.12
Adenylosuccinate synthetase isozyme 1	Adssl1	*0.90±0.07	1.41±0.17	*0.79±0.06
Mitochondrial import inner membrane translocase subunit Tim8 B	Timm8b	*0.90±0.06	*0.77±0.10	1.09±0.14
Lipid droplet-associated hydrolase	Ldah	*0.91±0.06	*0.75±0.08	*0.75±0.03
Inositol 1,4,5-trisphosphate receptor type 2	Itpr2	*0.91±0.08	*0.87±0.06	0.88±0.25
DnaJ homolog subfamily A member 1	Dnaja1	*0.91±0.07	*0.91±0.06	1.04±0.10
Peptidyl-tRNA hydrolase 2, mitochondrial	Ptrh2	*0.91±0.05	*0.89±0.07	0.98±0.18
Serine/threonine-protein kinase TAO1	Taok1	*0.91±0.06	*0.90±0.07	*0.80±0.08

These values represent average (± standard deviation) fold-change of abundance ratios for each altered (downregulated) protein compared to the high-fat control group (MS-NASH mice on a high-fat diet) with a 1.1-fold change threshold in response to Aquamin intervention and are significant with a p-value <0.05 (*). For each upregulated protein with Aquamin, corresponding values from the other two groups are shown for comparison. These liver samples (from 5 mice in each group) were individually assessed by TMT-based differential proteomic expression and data were merged to get averages. These data are also presented in Figure 4.
