# Supplementary material for: Liver Protein Expression in NASH Mice on a High-Fat Diet: Response to Multi-Mineral Intervention
Source: Front Nutr. 2022 May 11;9:859292. doi: 10.3389/fnut.2022.859292 (PMC9130755; doi:10.3389/fnut.2022.859292)
Supplement: Supplementary Table 1 — Mineral Composition of Aquamin® Soluble. [file Data_Sheet_1.zip › SM Table 7 859292.pdf]

Supplement Table 7. Top pathways associated with significantly downregulated proteins (shown in S Table 6) altered with Aquamin

Pathway name	Entities pValue	Mapped entities
Branched-chain amino acid catabolism	1.20×10^{-5}	Acadsb;Mccc1;Mccc2;Dbt;Ivd
Metabolism of amino acids and derivatives	2.88×10^{-4}	Hal;Suox;Acadsb;Agxt;Mccc1;Slc25a21;Mccc2;Prodh2;Dbt;Cdo1;Ivd;Ahcy
Synthesis of dolichyl-phosphate mannose	8.95×10^{-4}	Dpm3;Dpm1
Metabolism	0.003	Cyp4a12a;Ces3b;Slc25a21;Slc10a1;Abcc2;Ndufs5;Acsm5;Minpp1;Acadsb;Suox;Agxt;Mccc1;Etnk2;Cdo1;Atp5e;Fech;Slc10a2;ATP8;Nt5e;Mccc2;Adssl1;Prodh2;Dbt;Mmaa;Cyb5b;Ivd;Itpr2;Ndufaf1;Cox7a2l;Cyp7b1;Ahcy;Hal;Etnppl;Sucla2;Tm7sf2;Sdc1;Glrx5;Vkorc1;Sult1b1
Sulfur amino acid metabolism	0.005	Suox;Cdo1;Ahcy
Mitochondrial translation elongation	0.008	Mrps25;Mrps6;Mrpl20;Mrps12;Gfm1
Glyoxylate metabolism and glycine degradation	0.010	Agxt;Prodh2;Dbt
Mitochondrial translation	0.010	Mrps25;Mrps6;Mrpl20;Mrps12;Gfm1
Biotin transport and metabolism	0.011	Mccc1;Mccc2
Synthesis of substrates in N-glycan biosythesis	0.012	Alg5;Dpm3;Dpm1;Gne
Synthesis of PE	0.013	Etnppl;Etnk2
Degradation of cysteine and homocysteine	0.013	Suox;Cdo1
Synthesis of IPs in the ER lumen	0.014	Minpp1
Biosynthesis of the N-glycan precursor (dolichol lipid-linked oligosaccharide, LLO) and transfer to a nascent protein	0.021	Alg5;Dpm3;Dpm1;Gne
Bile acid and bile salt metabolism	0.023	Slc10a2;Slc10a1;Cyp7b1
Recycling of bile acids and salts	0.025	Slc10a2;Slc10a1
Formation of ATP by chemiosmotic coupling	0.028	ATP8;Atp5e
Cristae formation	0.028	ATP8;Atp5e
Respiratory electron transport, ATP synthesis by chemiosmotic coupling, and heat production by uncoupling proteins	0.028	ATP8;Atp5e;Ndufaf1;Ndufs5;Cox7a2l

Transport of glycerol from adipocytes to the liver by Aquaporins	0.028	Aqp9
Synthesis of dolichyl-phosphate-glucose	0.028	Alg5
Biological oxidations	0.033	Cyp4a12a;Ces3b;Cyb5b;Cyp7b1;Acsm5;Ahcy;Sult1b1
Metabolism of vitamins and cofactors	0.035	Nt5e;Mccc1;Mccc2;Sdc1;Mmaa;Vkorc1
The citric acid (TCA) cycle and respiratory electron transport	0.037	ATP8;Sucla2;Atp5e;Ndufs5;Ndufaf1;Cox7a2l
Mitochondrial translation termination	0.039	Mrps25;Mrps6;Mrpl20;Mrps12
Metabolism of vitamin K	0.042	Vkorc1
Proline catabolism	0.042	Prodh2

The pathways listed here are altered by the significantly downregulated proteins with the intervention "Aquamin" presented in Supplement Table 6. Reactome (v78) was used to generate the pathway analysis report for species *Mus musculus.* The significance (p-value) is calculated by the overrepresentation analysis (hypergeometric distribution).
